# *Toxoplasma gondii* infection in domestic and wild felids as public health concerns: a systematic review and meta-analysis

**DOI:** 10.1038/s41598-021-89031-8

**Published:** 2021-05-04

**Authors:** Kareem Hatam-Nahavandi, Rafael Calero-Bernal, Mohammad Taghi Rahimi, Abdol Sattar Pagheh, Mehdi Zarean, Asiyeh Dezhkam, Ehsan Ahmadpour

**Affiliations:** 1 School of Medicine, Iranshahr University of Medical Sciences, Iranshahr, Iran; 2 SALUVET, Animal Health Department, Faculty of Veterinary Sciences, Complutense University of Madrid, Madrid, Spain; 3 Center for Health Related Social and Behavioral Sciences Research, Shahroud University of Medical Sciences, Shahroud, Iran; 4 Infectious Diseases Research Canter, Birjand University of Medical Sciences, Birjand, Iran; 5 Department of Parasitology and Mycology, Faculty of Medicine, Mashhad University of Medical Sciences, Mashhad, Iran; 6 Infectious and Tropical Diseases Research Center, Tabriz University of Medical Sciences, Tabriz, Iran; 7 Immunology Research Center, Tabriz University of Medical Sciences, Tabriz, Iran; 8 Department of Parasitology and Mycology, Tabriz University of Medical Sciences, Tabriz, Iran

**Keywords:** Microbiology, Diseases

## Abstract

Felidae as definitive hosts for *Toxoplasma gondii* play a major role in transmission to all warm-blooded animals trough oocysts dissemination. Therefore the current comprehensive study was performed to determine the global status of *T. gondii* infection in domestic and wild felids aiming to provide comprehensive data of interest for further intervention approaching the One Health perspective. Different databases were searched by utilizing particular key words for publications related to *T. gondii* infecting domestic and wild feline host species, worldwide, from 1970 to 2020. The review of 337 reports showed that the seroprevalence of *T. gondii* in domestic cats and wild felids was estimated in 37.5% (95% CI 34.7–40.3) (*I*^2^ = 98.3%, *P* < 0.001) and 64% (95% CI 60–67.9) (*I*^2^ = 88%, P < 0.0001), respectively. The global pooled prevalence of oocysts in the fecal examined specimens from domestic cats was estimated in 2.6% (95% CI 1.9–3.3) (*I*^2^ = 96.1%, *P* < 0.0001), and that in fecal samples from wild felids was estimated in 2.4% (95% CI 1.1–4.2) (*I*^2^ = 86.4%, *P* < 0.0001). In addition, from 13,252 examined soil samples in 14 reviewed studies, the pooled occurrence of *T. gondii* oocysts was determined in 16.2% (95% CI 7.66–27.03%). The observed high rates of anti-*T. gondii* antibodies seroprevalence levels and oocyst excretion frequency in the felids, along with soil (environmental) contamination with oocysts may constitute a potential threat to animal and public health, and data will result of interest in further prophylaxis programs.

## Introduction

*Toxoplasma gondii* is an opportunistic and successful coccidian parasite capable of infect virtually all homoeothermic vertebrates, including human beings^1,2^. Domestic cats and other Felidae constitute its specific definitive hosts^3^, and all non-feline animals are regarded as intermediate hosts; however, *T. gondii* can also undergo asexual reproduction in tissues of Felidae acting as intermediate hosts. First, tachyzoites have active multiplication in tissues, associated to rapid invasion causing harmful effects. Zoites present a special tropism to central nervous system and striated muscle, in which they remain latent confined in a cyst as bradyzoites, leading to a long-term chronic infection until another definitive host ingests the tissue. Then, released bradyzoites penetrate the epithelial cells of small intestine, giving rise to schizonts that will form gamonts and, finally, oocysts^4^. Felids excrete oocysts in their faeces, during a limited time lapse, contaminating soil and water^5–8^. In addition to the domestic cats, and under the view of the available literature, the role of wild Felidae in the epidemiology of *T. gondii* should not be neglected^5,9^. Therefore, felids constitute the key element in the epidemiology of *T. gondii* since an individual can shed millions of oocyts that can spread the infection to many other susceptible hosts^10^. Several important outbreaks of human toxoplasmosis were epidemiologically linked to oocyst contamination of drinking water^11–13^. By the way, oocysts were not detected in the samples collected from the water reservoir linked to a serious Canadian outbreak^14^, but viable oocysts were observed in contents of the intestine of a wild trapped cougar (*Felis concolor vancouverensis*) and in a fecal pile in close proximity to the reservoir^15^. It is important to highlight that the sporulated oocysts are very resistant and can remain viable and infective for more than 1 year in favourable conditions^11,16–18^. In this regard, a recent paper reviewed the environmental pathways by which *T. gondii* can infect animals and people mostly driven by water, soil or contaminated fresh produce or seafood^19^.

*Toxoplasma gondii* antibodies have been largely found in cats worldwide, and the seroprevalence degree increases with the age of the cat, suggesting postnatal transmission of *T. gondii*^20^. It is assumed that postnatal sero-conversion in cats is linked to oocysts excretion episodes. The life style of cats influences the occurrence of *T. gondii* infections since feral cats that hunt for their food will present higher rates than domestic cats with limited access to parasites^21^. Seroprevalence level varied among continents, countries and even cities, linked to many possible environmental factors influencing these variations. As an example, in an urban population of 301 domestic cats in Lyon, France^20^, the anti-*T. gondii* seroprevalence was only 18.6%, approximately half the prevalence in other surveys in Europe^22,23^. The control of rodents in the area and feeding of cats by people were considered as protective factors limiting infections. On the other hand, a low income and poor sanitation were not the determining factors for low seropositivity to *T. gondii* in cats in Durango, Mexico^24^. Since a high density of felines (specially domestic cats) increases the risk of infection and *T. gondii* prevalence in intermediate hosts, a gradient of prevalence rate of infection has been demonstrated depending on the anthropization degree of the environment^25,26^.

Nearly up to 30% of the world’s human population has had contact with the parasite evidenced by the presence of anti-*T. gondii* antibodies; while *T. gondii* infections are usually asymptomatic, they can lead to harmful effects, especially in congenital cases and immunocompromissed persons^27,28^. Humans become primarily infected mostly via oral ingestion of viable tissue cysts present in raw or undercooked meat and oocysts contaminating water or foodstuffs^6,8,29^. Nowadays, comprehensive local studies are still necessary to determine the source attribution of human infections; this constitutes an interesting challenge that should be approached under the One Health perspective.

To date, different surveys have been focused on domestic and wild felids in order to determine aspects as seroprevalence rates of anti-*T. gondii* antibodies, frequency of oocysts excretion and soil presence worldwide^30–36^, but with a certain degree of variance among studies. A systematic review recently assessed the seroprevalence of *T. gondii* in felids from 1967 to 2017 with a search strategy restricted to articles in English^37^. So that, the present investigation was aimed to determine the global frequency of *T. gondii* infections in domestic cats and wild felids, the occurrence of *T. gondii*-like oocysts shedding, and the frequency of oocysts in soil; such information will be useful to implement further measures aiming to reduce animal and human infections under a One Health perspective.

## Methods

### Search strategy

The review process exactly followed the protocol suggested by the Preferred Reporting Items for Systematic Reviews and Meta-analyses (PRISMA) guidelines (Supplementary data: PRISMA/STROBE)^38^. We retrieved published studies from the databases of MEDLINE (via PubMed) (https://www.ncbi.nlm.nih.gov/pubmed/), Scopus (https://www.scopus.com/), Web of Science (https://www.webofknowledge.com/) and CAB Abstracts (https://www.cabi.org/AHPC) with no restriction on language from Jan 1, 1970, to Dec 31, 2019. Search terms included a combination of Medical Subject Heading terms (MeSH) and free-text words in titles, abstracts and full texts.

The systematic search for PubMed accomplished using several Medical Subject Heading terms (Table S1). In addition, Scopus, Web of Science and CAB Abstracts were searched using the same strategy (Supplementary DATA). The Google Scholar search engine was used for checking the search strategy. The reference lists of all included articles and relevant reviews were hand searched for potentially eligible literature. In addition, authors and experts in the field were consulted to aid in the identification of relevant conference abstracts related to *Toxoplasma* and toxoplasmosis. Sometimes, we have had to contact the authors for raw data collection^39^, especially in old literature.

### Selection of studies

Initial screening by manuscript titles and abstracts was performed independently by two researchers (KHN and EA), that also assessed the full texts of all potentially relevant studies and applied inclusion criteria. Discrepancies when detected, were resolved after constructive discussion (AD, MZ and MTR).

The studies providing data on the seroprevalence of *T. gondii* in domestic or wild felids, frequency of oocyst excretion in felids, and those reporting soil contaminations with oocysts were included. On the other hand, studies meeting the epidemiology of *T. gondii* in non-feline hosts, studies where cat faecal samples were collected from the ground, and data from each animal was not independently retrievable, experimental studies, articles that only presented the final result and did not provide the raw data, or those without definite sample size, abstracts presented in congresses without full text, and case–control studies and clinical trials that could not report a correct estimate of prevalence were excluded. Any duplicated research was also excluded.

### Quality assessment

The standard Strengthening the Reporting of Observational Studies in Epidemiology (STROBE) checklist was used^40^. In present study, articles were evaluated as low quality: less than 16.5, moderate quality: 16.6–25.5, and high quality: 25.6–34; the articles included in the meta-analysis presented acceptable quality.

### Data extraction

After comprehensive examination of selected articles, the following data were extracted: the first author’s last name, publication year, country, feline scientific names, keeping status of felids, sample size, source of soil samples, beginning and end date of study implementation, the number of the positive and negative cases, cut-off, age groups, as well as information about diagnostic tools. Data were extracted separately if two different populations had been studied. All extracted data from each study were entered into an Excel spreadsheet.

### Meta-analysis

The collected data were entered into the StatsDirect statistical software package (version 2.7.2) (Stata Corporation, College Station, TX, USA) (http://www.statsdirect.com/). Statistical heterogeneity of the different years among studies was assessed using the Cochrane’s Q test and inconsistency *I*^2^ test. To determine whether there is a significant heterogeneity, a random effect model was used to estimate the pooled prevalence’s of cat infection^41^. In addition, potential publication bias was explored using Funnel plot and Egger’s test.

## Results

**Figure 1:**
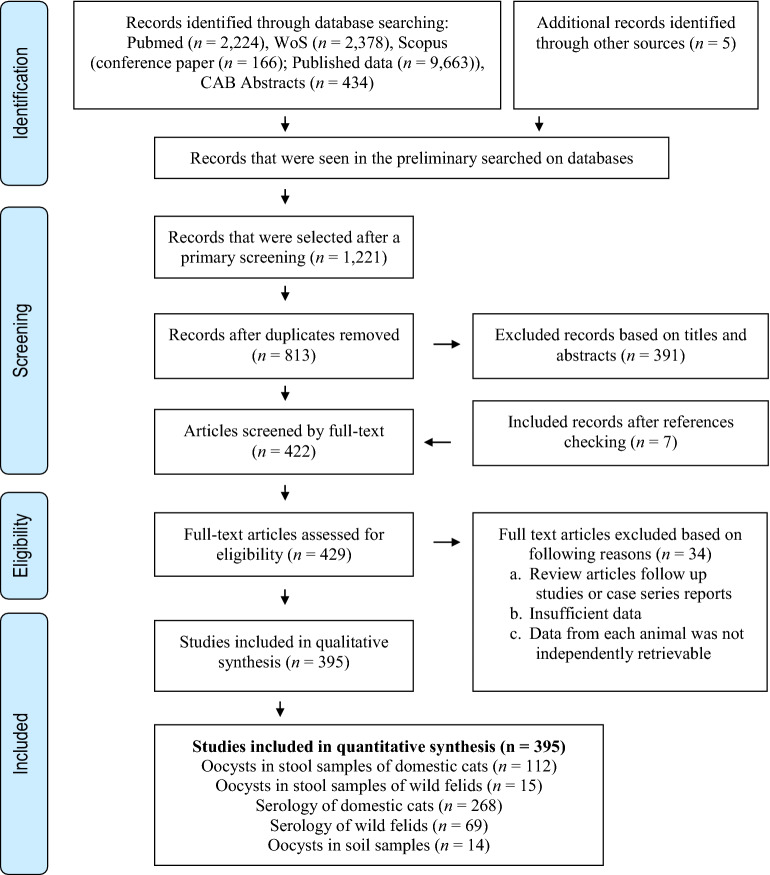
Flowchart of the study design process.

**Figure 2:**
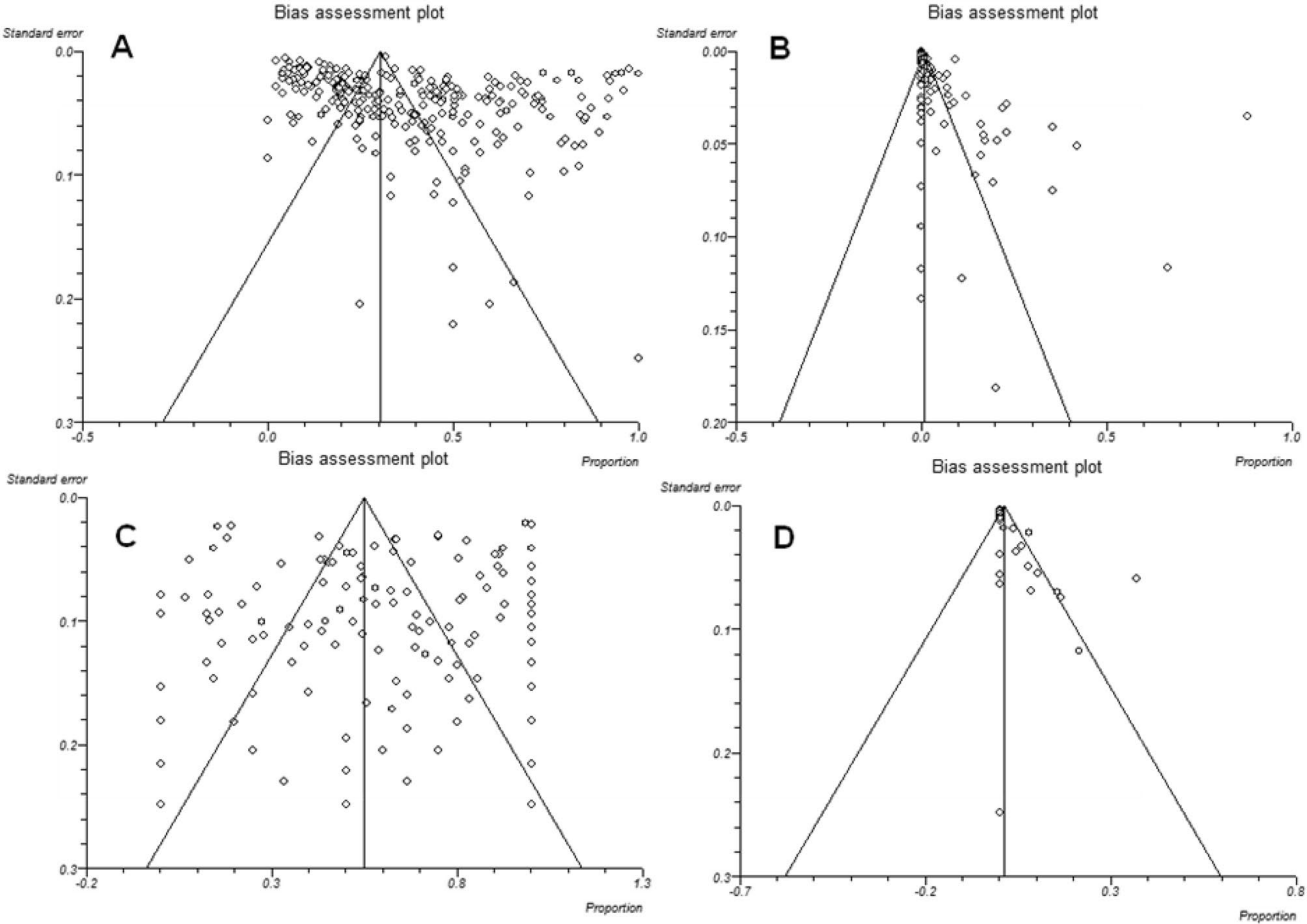
Funnel plot of standard error by logit event rate to assess publication or other types of bias across prevalence studies. (**A**) Studies based on the seroprevalence of anti-*Toxoplasma gondii* antibodies in domestic cats, (**B**) studies based on detection of *T. gondii*-like oocyst and *T. gondii* oocyst DNA in domestic cat feces, (**C**) studies based on the seroprevalence of anti-*Toxoplasma gondii* antibodies in wild felids, (**D**) studies based on detection of *T. gondii*-like oocyst and *T. gondii* oocyst DNA in wild felids feces.

The initial database search retrieved 14,870 publications. First screening enabled us to exclude 14,441 studies not meeting the inclusion criteria. Altogether, 429 studies were retained for further investigation. In a secondary assessment, another 34 documents were excluded because of one of the following reasons: review articles, follow up studies or case series reports, and papers with insufficient data, or data from each animal were not independently retrievable. Eventually, 395 studies which met our eligibility criteria and evaluated soil contamination with *T. gondii* oocysts (*n* = 14), and *T. gondii* infection in domestic cats (serology, *n* = 268; oocyst in feces, *n* = 112) and wild felids (serology, *n* = 69; oocyst in feces, *n* = 15) during five decades were retained for analysis (Fig. 1). Potential publication bias in the conducted studies regarding the prevalence of *T. gondii* infection in domestic cats and wild felids, *Toxoplasma*-like oocysts shedding, and frequency of oocysts in soil are shown using Funnel plot and Egger’s test (Fig. 2).

### Prevalence of anti-*T. gondii* antibodies in blood/serum samples

The global pooled seroprevalence of *T. gondii* in domestic cats was estimated in 37.5% (95% CI 34.7–40.3) (*I*^2^ = 98.3%, *P* < 0.001) (Table 1). The highest rate was observed in Australia (66.6%, 95% CI 62.8–70.3) (*I*^2^ = 97.2%, *P* < 0.0001), followed by Africa (55.7%, 95% CI 35.6–74.8) (*I*^2^ = 98.9%, *P* < 0.0001), Europe (45.3%, 95% CI 41.1–49.6) (*I*^2^ = 96.7%, *P* < 0.0001), Central and South America (40.3%, 95% CI 34–49.6) (*I*^2^ = 97.7%, *P* < 0.0001) and North America (31.6%, 95% CI 27–36.4) (*I*^2^ = 96.8%, *P* < 0.0001). The lowest prevalence was observed in Asia (28.3%, 95% CI 24.1–32.6) (*I*^2^ = 98.1%, *P* < 0.0001) (Table 1). In the other hand, the worldwide pooled seroprevalence of *T. gondii* in wild felids (including lion, jaguarundi, jaguar, ocelot, cougar, leopard, tiger, geoffroy's cat, oncilla, margay, caracal, snow leopard, Eurasian lynx, bobcat, cheetah, Prionailurus cats, Iberian lynx, pampas cat, serval, pallas's cat, jungle cat, European wildcat, sand cat, Asian golden cat, Canadian lynx, clouded leopard, masked palm civet, and common genet) was estimated 64% (95% CI 60–67.9) (*I*^2^ = 88%, *P* < 0.0001) (Table 2). The highest and the lowest seroprevalence rates were related to *Panthera leo* (87.6%, 95% CI 79–94.3) and *Leopardus colocolo* (19.6%, 95% CI 6.1–38.3), respectively (Table 2). Heterogeneity was, however, very low *I*^2^ = 79.9%. Retrieved information of the eligible studies based on the of anti-*Toxoplasma* antibodies prevalence in domestic cats and wild felids are summarized in Tables S2 and S3. In total, 80,087 blood samples from domestic cats (*n* = 73,980) and wild felids (*n* = 6,107) from 337 eligible studies were examined for the presence of anti*-T. gondii* antibodies and/or *T. gondii* DNA, of which 26,903 subjects were diagnosed as positive (domestic cats *n* = 23,593; wild felids *n* = 3,310) (Tables S2, S3). Different diagnostic methods to evaluate anti*-T. gondii* antibodies in the cats have been identified in the studies: MAT (132 studies), IFAT (99 studies), and ELISA (92 studies) (Tables S2, S3). Amongst the reviewed studies, just one investigation applied PCR method for detection of *T. gondii* DNA in stray cats using blood samples^42^ (Table S2).

**Table 1:** Pooled prevalence of *Toxoplasma* infection in domestic cats and subgroup analyses.

Continent	No. of studies	Detection method	Prevalence % (95% CI)	Heterogeneity			Egger’s test	
				**I**_**2**_	**Q**	***P value***	T	*P* value
Africa	6	Stool exam	9.8 (2.4–21.5)	94.1	84.2	< 0.0001	5.1	0.0434
	16	Serology	55.7 (35.6–74.8)	98.9	1408.4	< 0.0001	− 3.8	0.6571
Asia	32	Stool exam	4 (1.9–6.9)	96.9	1001.4	< 0.0001	3.2	0.0195
	90	Serology	28.3 (24.1–32.6)	98.1	4730.5	< 0.0001	7.7	< 0.0001
Australia	4	Stool exam	1.7 (0.2–4.5)	79.1	14.3	0.0025	1.3	0.3488
	6	Serology	66.6 (62.8–70.3)	97.2	176.1	< 0.0001	− 9.66	0.1256
Europe	55	Stool exam	1.21 (0.8–1.6)	89.6	517.1	< 0.0001	1.18	< 0.0001
	61	Serology	45.3 (41.1–49.6)	96.7	1840.1	< 0.0001	2.94	0.1269
North America	16	Stool exam	0.9 (0.5–1.3)	50.0	30.3	0.0108	0.94	0.0583
	37	Serology	31.6 (27–36.4)	96.8	1126.2	< 0.0001	0.49	0.7212
Central/South America	12	Stool exam	6.2 (1.8–1.3)	97.1	374.2	< 0.0001	3.4	0.0226
	61	Serology	40.3 (34.0–46.8)	97.7	2642.3	< 0.0001	4.5	0.0467

**Table 2:** Pooled prevalence of *Toxoplasma* infection in wild felids and subgroup analyses.

Host species	No. of studies	Detection method	Prevalence (95% CI)	Heterogeneity			Egger’s test	
				I_2_	Q	*P* value	T	*P* value
Asian golden cat (*Catopuma temminckii*)	3	Serology	47.1 (8.9–87.4)	72.7	7.3	0.0256	–	–
Bobcat (*Lynx rufus*)	18	Serology	60.5 (47.1–73.1)	91.0	189.8	< 0.0001	1.5	0.3255
	5	Stool exam	4.1 (0.2–12.4)	82.5	22.8	0.0001	1.0	0.1297
Canadian Lynx (*Lynx canadiensis*)	3	Serology	36.4 (10.8–67.2)	91.7	24.1	< 0.0001	–	–
Caracal (*Caracal caracal*)	7	Serology	69.9 (49.6–86.8)	0.0	5.1	0.5270	− 0.3	0.9283
Cheetah (*Acinonyx jubatus*)	8	Serology	70.4 (48.1–88.5)	81.0	36.7	< 0.0001	− 0.6	0.7904
Clouded leopard (*Neofelis nebulosa*)	4	Serology	36.3 (9.0–69.7)	65.8	8.7	0.0325	4.2	0.2131
Cougar (*Puma concolor*)	24	Serology	56.1 (43.7–68.2)	93.8	371.7	< 0.0001	2.6	0.1155
	6	Stool exam	4.7 (0.5–12.7)	61.5	12.9	0.0236	0.8	0.0955
Eurasian lynx (*Lynx lynx*)	6	Serology	42.1 (14.9–72.2)	97.4	192.1	< 0.0001	1.7	0.7428
European wildcat (*Felis silvestris*)	7	Serology	76.8 (62.6–88.5)	46.0	11.1	0.0848	0.5	0.6714
Geoffroy's cat (*Leopardus geoffroyi*)	5	Serology	60.7 (39.9–79.6)	60.8	10.1	0.0373	1.7	0.6131
Iberian Lynx (*Lynx pardinus*)	4	Serology	66.2 (50.1–80.5)	81.9	16.5	0.0009	2.9	0.6198
Jaguar (*Panthera onca*)	9	Serology	74.4 (63.5–84)	61.6	20.8	0.0076	1.7	0.1306
	3	Stool exam	3.5 (1.3–13.7)	66.9	6.0	0.0487	–	–
Jaguarundi (*Herpailurus yagouaroundi*)	10	Serology	47.7 (41.6–53.9)	0.0	8.5	0.4774	1.9	0.0053
Jungle cat (*Felis chaus*)	2	Serology	44.5 (7.5–97.1)	–	7.9	0.0047	–	–
Leopard (*Panthera pardus*)	17	Serology	68.0 (46.5–86.1)	72.5	58.1	< 0.0001	5.1	0.0010
	3	Stool exam	3.8 (0.1–17.6)	84.9	13.2	0.0013	–	–
Lion (*Panthera leo*)	20	Serology	87.6 (79–94.3)	79.9	94.6	< 0.0001	− 1.6	0.0175
	3	Stool exam	4.9 (0.3–23.8)	85.6	13.8	1.0010	–	–
Margay (*Leopardus wiedii*)	5	Serology	56.0 (46.4–65.4)	29.0	5.6	0.2282	2.3	0.2958
Ocelot (*Leopardus pardalis*)	11	Serology	66.2 (58.1–73.8)	45.6	18.3	0.0489	1.6	0.0921
	3	Stool exam	15.9 (0.2–58.6)	85.5	13.7	0.0010	–	–
Oncilla (*Leopardus tigrinus*)	9	Serology	59.0 (49.7–68)	47.9	15.3	0.0527	1.4	0.2027
Pallas's cat (*Otocolobus manul*)	10	Serology	70.6 (43.9–91.3)	89.3	84.1	< 0.0001	− 2.4	0.4067
Pampas cat (*Leopardus colocolo*)	3	Serology	19.6 (6.1–38.3)	0.0	0.7	0.6878	–	–
Prionailurus cats (*Prionailurus viverrinus*)	10	Serology	39.6 (24.3–56.1)	60.0	22.4	0.0075	2.5	0.0606
	5	Stool exam	4.0 (1.8–7.1)	0.0	1.4	0.8433	− 0.2	0.4709
Sand cat (*Felis margarita*)	4	Serology	70.5 (49.9–87.5)	66.8	9.0	0.0287	3.0	0.2817
Serval (*Leptailurus serval*)	4	Serology	64.3 (35 to88.6)	8.8	3.2	0.3493	-4.1	0.7970
Snow leopard (*Panthera uncial*)	2	Serology	52.6 (10.7–92.2)	–	1.9	0.1607	–	–
Tiger (*Panthera tigris*)	16	Serology	66.2 (51.4–79.5)	61.9	39.3	0.0006	1.3	0.3862
	4	Stool exam	7.4 (0–27.4)	80.2	15.1	0.0017	1.8	0.2827

### Occurrence of *T. gondii* oocysts in fecal samples

A total number of 137 eligible studies which examined 66,601 fecal samples from domestic cats (*n* = 63,458) and wild felids (*n* = 3,143), 1,330 were positive (domestic cats *n* = 1,254; wild felids *n* = 76) for *T. gondii* oocysts, *T. gondii*-like oocysts, and/or *T. gondii* DNA (Table S4, S5). The global pooled prevalence of oocysts in the fecal examined specimens from domestic cats was estimated in 2.6% (95% CI 1.9–3.3) (*I*^2^ = 96.1%, *P* < 0.0001) (Table 3). The highest and the lowest prevalence rates were detected in Africa (9.8%, 95% CI 2.4–21.5) (*I*^2^ = 94.1%, *P* < 0.0001), and North America (0.9%, 95% CI 0.5–1.3), respectively. Heterogeneity was, however, very low *I*^2^ = 50% (Tables 1, 3). The most used methodology for detection of oocysts was microscopy (99 studies) which was followed by molecular (16 studies) and mouse bioassay (10 studies) methods (Table S4). Some studies combined two techniques for detection of oocysts in feline feces. The highest prevalence was related to the molecular detection method (6.5%, 95% CI 3.7–10) (*I*^2^ = 92.1%, *P* < 0.0001), followed by bioassay (2.8%, 95% CI 0.6–6.4) (*I*^2^ = 95.1%, *P* < 0.0001), and microscopy (2.1%, 95% CI 1.4–2.8) (*I*^2^ = 96.3%, *P* < 0.0001) (Table 3).

**Table 3:** The global pooled prevalence of *Toxoplasma* infection in feline hosts/felids.

Group	Number of studies	Pooled prevalence (95% CI)	Heterogeneity		
			*P* value	I^2^	Cochran Q
**Domestic Cat**					
Serology	**271**	**37.5 (34.7–40.3)**	** < 0.0010**	**98.3**	**15,984.3**
**Stool exam**	**125**	**2.6 (1.9–3.3)**	** < 0.0001**	**96.1**	**3164.3**
Microscopy	99	2.1 (1.4–2.8)	< 0.0001	96.3	2664.6
Bioassay	10	2.8 (0.6– 6.4)	< 0.0001	95.1	182.7
Molecular	16	6.5 (3.7–10)	< 0.0001	92.1	189.3
**Wild Feline**					
Serology	**223**	**64.0 (60–67.9)**	** < 0.0001**	**88**	**1854.9**
Stool exam	**12**	**2.4 (1.1–4.2)**	** < 0.0001**	**86.4**	**227.5**

**Figure 3:**
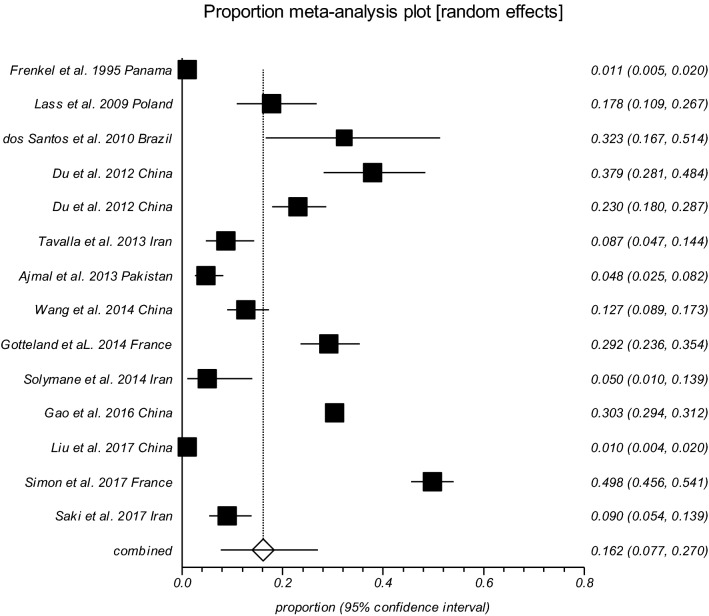
Forest plot diagram of the present systematic review and meta-analysis based on studies focused on detection of soil contamination by *Toxoplasma*-like oocysts.

The worldwide pooled prevalence of *T. gondii* oocysts, *T. gondii*-like oocysts and *T. gondii* DNA in fecal specimens from wild felids was estimated in 2.4% (95% CI 1.1–4.2) (*I*^2^ = 86.4%, *P* < 0.0001) (Table 3). The highest and the lowest prevalence of oocysts in fecal specimens was related to *Leopardus pardalis* (15.89%, 95% CI 0.2–58.6) and *Panthera onca* (3.5%, 95% CI 1.3–13.7), respectively (Table S5). The prevalence of *Toxoplasma*-like oocysts detected in domestic and wild feline stool samples in different countries are shown in Fig. 3. As well India (49%) and Colombia (33%) had the highest prevalence.

### Occurrence of *T. gondii* oocysts in soil samples

**Figure 4:**
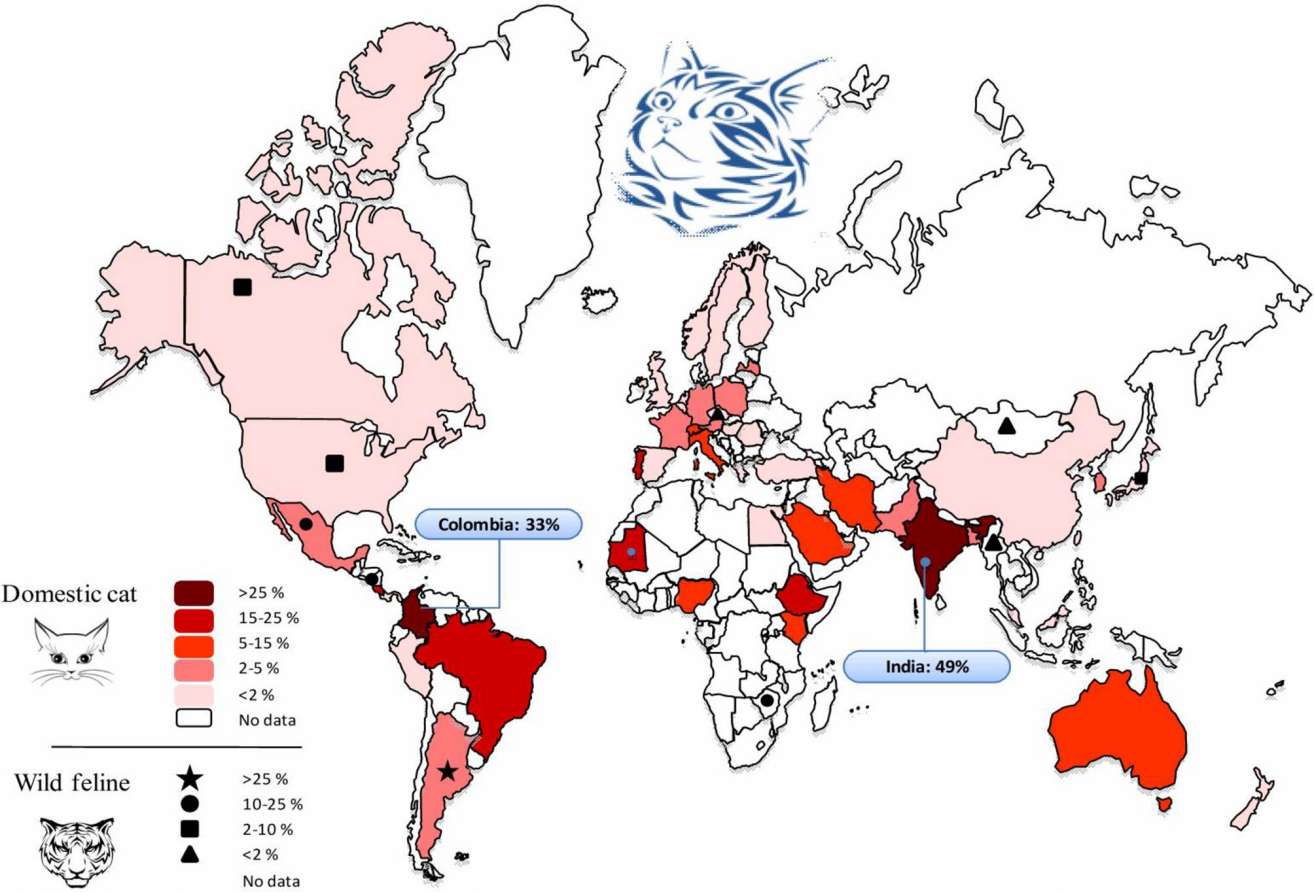
Pooled prevalence of *Toxoplasma*-like oocysts detected in domestic and wild feline stool samples in different countries (Map created by PowerPoint Microsoft office, source of image: https://commons.wikimedia.org/wiki/File:BlankMap-World.svg).

Up to 14 studies reported the examination of 13,252 soil specimens resulting in 3,421 (25.8%) samples positive for *T. gondii* oocysts (or *T. gondii*-DNA) using mouse bioassay and different PCR procedures. Table S6 shows the conducted studies to detect *T. gondii* oocysts in soil samples; the pooled prevalence of *T. gondii* oocysts in those samples was estimated 16.2% (95% CI 7.66–27.03%) (Q = 1628.10, *I*^*2*^ = 99.2%, *P* < 0.0001) (Egger’s bias = 5.44, *P* = 0.3733) (Fig. 4).

## Discussion

Felids as final host play an irreplaceable role for *T. gondii* life cycle that exclusively yield and excrete oocysts in their faeces, contaminating soil, water and food^6–8,10^. According to our findings, 37.5% of domestic cats showed exposure to *T. gondii* and 2.6% were actively shedding *T. gondii* or *T. gondii*-like oocysts. Similarly, the worldwide seroprevalence of *Toxoplasma* in domestic cats had been previously estimated at levels of 30–40%^1,43^.

Based on the results, the highest value of seroprevalence in domestic cats was observed in Australia, followed by Africa, Europe, Central/South America, North America and Asia; although the number of studies for Australia (*n* = 6) and Africa (*n* = 15) were relatively low, whereas only one study in USA included 12,628 animals. The lowest prevalence was observed in Asia (28.3%), nevertheless, most studies have been conducted in Asian countries (91 studies). The number of surveys was higher in USA (North America; 34 studies), followed by Brazil (South America; 30 studies). Our investigation identified a number of countries without any data on *T. gondii* infection in cats, emphasizing the need for further studies in this field. According to our findings, 64% of nondomestic cats showed evidence of exposure to *T. gondii* and 2.4% were actively shedding *T. gondii* oocysts. Accordingly, the highest sero-prevalence of *T. gondii* in different wild felids were in the following order: lion (*Panthera leo*), European wildcat (*Felis silvestris*), jaguar (*Panthera onca*), Pallas's cat (*Otocolobus manul*), sand cat (*Felis margarita*), cheetah (*Acinonyx jubatus*), caracal (*Caracal caracal*), leopard (*Panthera pardus*), Iberian lynx (*Lynx pardinus*), ocelot (*Leopardus pardalis*), tiger (*Panthera tigris*), serval (*Leptailurus serval*), Geoffroy's cat (*Leopardus geoffroyi*), bobcat (*Lynx rufus*), oncilla (*Leopardus tigrinus*), cougar (*Puma concolor*), margay (*Leopardus wiedii*), snow leopard (*Panthera uncial*), jaguarundi (*Herpailurus yagouaroundi*), Asian golden cat (*Catopuma temminckii*), Eurasian lynx (*Lynx lynx*), jungle cat (*Felis chaus*), Prionailurus cat (*Prionailurus viverrinus*), Canadian lynx (*Lynx canadiensis*), clouded leopard (*Neofelis nebulosa*), and Pampas cat (*Leopardus colocolo*). Although in general it should be considered that the number of studied nondomestic cats were lower compared to domestic cats, and keeping status of wilds cats, in captive (*n* = 2,492) or free ranging (*n* = 2,949), is probably associated with how they are fed and how they become infected. Albeit such findings also highlights the importance of serological and isolation studies on *T. gondii* infecting their prey (ungulates, birds, etc.) using validated methodologies for bias reducing.

The considerable gap between the prevalence of oocysts in feces and positive serum antibodies can be explained by the fact that infected felids can shed *T. gondii* oocysts for a short period (10–15 days), shortly after primoinfection, and then they become seropositive indefinitely. As, one important point, the activation of humoral immunity and antibodies production prevent from re-shedding of oocysts; new excretion episodes can occur when severe immunosuppression appears^29,44^. The short period of oocyst shedding and the low prevalence rate of felids which actively excrete oocysts, have led some authors to discuss that direct contact with felids should not be considered as a risk factor for human infection^7,29,45^. A systematic review in Iran showed that humans with history of close contact with cats presented a higher *T. gondii* seroprevalence rate compared to those without contact^46^. In the study conducted by Jones and colleagues^29^ in the USA, exposure to kittens was statistically linked to *T. gondii* infection. In the study conducted in different European centers^45^, infections in pregnant women were attributed to the consumption of undercooked or cured meat products and soil contact in the 30–63% and 6–17% of cases respectively, but contact with cats was not identified as a risk factor. Similarly, another study showed that contact with cats is not related to infections, while the ingestion of raw or undercooked meat highly increased the risk of infection^47^.

Based on the results, 16.2% of soil samples contained *T. gondii* oocysts (or *T. gondii*-DNA), those when sporulated can survive for several months under tough conditions and are resistant to common disinfectants^48^. Contaminated soil has been demonstrated as an important source for infection for humans and animals^13,19^. It has been shown that gardening and occupations in contact with soil increases the risk of *T. gondii* infection^49^, as previously seen^50^. In a follow-up study of the toxoplasmosis outbreak during 1977 in Georgia^51^, after 25 years, among 37 individual (exposure to an indoor horse arena), 14 equestrian were tested, that three (21%) were found to have toxoplasmic retinochoroiditis lesions. Based on the observations is possible that cat feces containing the organism were most likely stirred up when horses ran on the dirt floor, and were inhaled or ingested by riders and observers. Based upon number of studies conducted in different European centers, contact with soil or vegetables or fruit presumably contaminated with soil were highly associated to *T. gondii* infection in pregnant women^45,52–54^. Investigation on sentinels (i.e., molluscs) for environmental contamination^55^ and also the infection source attribution by using specific tests^56^ will be of great interest for integration with data compilation in definitive and intermediate susceptible hosts.

In the present investigation, the prevalence of soil contamination was highly variable in the selected studies, which might be influenced by the soil characteristics and the number of infected animals in the area^57^. The included studies also reported highly heterogeneous results regarding the prevalence of cat infections, which could be due to the different risk factors, to note: sex, age, climates, study periods, cat breeds, living conditions and diagnostic methods as well as other unrecognized confounding factors. Based on our results, *T. gondii* seroprevalence in cats (*Felis domesticus*) in different countries oscillated from less than 10% in Thailand, Taiwan and Angola to more than 70% in Qatar, and Ethiopia. This can partly be explained by the different environmental conditions among the countries^58^. It has been shown that cat infections present higher occurrence in warm, moist and low altitude regions, maybe linked to oocysts sporulation and survival of *T. gondii* oocysts in such latitudes^34^. Similarly, *T. gondii* seroprevalence in pigs was associated with lower geographical latitude and higher mean annual temperature^59^, fact that may suggest high environmental contamination with *T. gondii* sporulated oocysts. It seems to be clear that oocysts shedding by cats constitute the essential element for sustainment of the parasite in the environment, this was demonstrated when extremely low seroprevalence of *T. gondii* (0.9%) was detected in feral pigs from a remote island lacking cats in the USA^60^. Furthermore, the time period of study might influence the results, as the infection rate is higher in autumn, winter^22^, and rainy years^20^. One may consider breed as a variable factor for cat *T. gondii* infection. It has been shown that *Toxoplasma* seroprevalence is highly variable in different cat breeds from 18.8% in Burmese cats to 60% in Persian cats^35^. Even though, a high occurrence rate of *T. gondii* infection in cat may be attributed to some important factors including: uncontrolled food and access to contaminated sources, wandering outdoor, humid and temperate climate; and cat abundance. Furthermore, stray cats have been shown to have a higher seroprevalence compared to pet cats, which can be explained by more access to contaminated source and outdoor living^31,34,61^. Additionally, pet cats with an outdoor access are also at an increased risk of infection compared to those kept indoor^32,35^. It has been reported that rural cats show a higher seroprevalence rate of *T. gondii* compared to urban ones^62^.

Furthermore, the different diagnostic methods used to detect *T. gondii* antibodies and oocysts could influence the results. While the different techniques used for anti-*T. gondii* antibodies detection showed comparably good diagnostic performance, most of the studies aiming to detect *T. gondii* oocysts employed less reliable microscopic methods, which might result in false positives, as oocysts and sporocysts of some other coccidia (e.g. *Hammondia hammondi*, *Besnoitia darlingi*) may resemble those of *T. gondii*^48^. It shows the necessity of testing environmental and fecal samples by using specific-PCR aided with amplicon sequencing for identity confirmation^63^.

Felids as key elements in the epidemiology of toxoplasmosis should be considered as a potential threat to animal and public health, due potential oocysts contamination of the environment; such information is still missing in several worldwide locations, so further epidemiological investigations on final hosts would be of special interest for evaluating the status of *T. gondii* infection and risk assessment implementations. Further investigations based on QMRA approaches^64,65^ combining raw data in Felidae with those from the environmental side and those from susceptible hosts will complement the One Health puzzle in defined areas.

In present meta-analysis, it is shown that about one-third of domestic and non-domestic cats have been exposed to *T. gondii*, and globally about 1 in 50 cats are actively shedding *T. gondii* or *T. gondii*-like oocysts. In addition, 16.2% of the soil samples examined were contaminated with *T. gondii*-like oocysts informing on a broad environmental distribution. Felids are the only final host of *T. gondii* and play a major role in its life cycle, therefore measures aiming to reduce environmental contamination with *T. gondii* oocysts will be of major interest, and a One Health perspective covering human, animal and environmental health should be taken into account.

## Supplementary Information

Supplementary Information 1.

Supplementary Information 2.

Supplementary Information 3.

Supplementary Information 4.

Supplementary Information 5.

Supplementary Information 6.

Supplementary Information 7.

Supplementary Information 8.

## Data Availability

The data that supports the findings of this study are available in the supplementary material of this article.
