# Supplementary material for: Toxoplasma gondii infection in domestic and wild felids as public health concerns: a systematic review and meta-analysis
Source: Sci Rep. 2021 May 4;11:9509. doi: 10.1038/s41598-021-89031-8 (PMC8097069; doi:10.1038/s41598-021-89031-8)
Supplement: Supplementary file 3 — Supplementary Information 3. [file 41598_2021_89031_MOESM3_ESM.doc]

**Supplementary file: Table S1**

| Syntax for PubMed  (Toxoplasm* OR *Toxoplasma* OR "*Toxoplasma gondii*" OR "*Toxoplasma gondius*" OR (*Toxoplasma* AND *gondii*) OR toxoplasmosis OR toxoplasmoses OR "*Toxoplasma* *gondii* infection" OR (*"Toxoplasma* *gondii*" AND infection) OR "*Toxoplasma gondii*-like oocysts" OR "*Toxoplasma gondii* oocyst" OR ("*Toxoplasma gondii*" AND oocyst)) AND (cat OR "domestic cat" OR (cat AND domestic) OR *Felis* OR "*Felis sylvestris catus*" OR "*Felis sylvestris gordoni*" OR "Gordon’s cat" OR "wild cat" OR "*Felis domesticus*" OR "*Felis domestica*" OR caracal OR "*Caracal caracal*" OR *Uncia* OR "*Uncia uncia*" OR leopard OR "snow leopard" OR (snow AND leopard) OR "panthera snow" OR "Panthera uncia" OR "clouded leopard" OR (clouded AND leopard) OR "*Neofelis nebulosa*" OR ocelot OR "Leopardus pardalis" OR cheetah OR "Acinonyx *jubatus*" OR bobcat OR "*Lynx* *rufus*" OR "*Lynx* *canadiensis*" OR "*Lynx* *lynx*" OR "*Lynx* *pardinus*" OR "Iberian lynx" OR polecat OR jaguar OR "Panthera onca" OR "*Panthera pardus*" OR "black panther" OR lion OR "*Panthera leo*" OR tiger OR "Panthera tigris" OR "*Panthera tigris altaica*" OR puma OR "Florida panther" OR "*Puma concolor coryi*" OR cougar OR "*Puma concolor*" OR "mountain lion" OR "*Felis concolor*" OR "*Felis concolor vancouverensis*" OR "*Felis chaus*" OR "*Felis euptilurus*" OR "Amur leopard cat" OR "*Felis margarita*" OR "sand cat" OR "*Felis manul*" OR "*Otocolobus manul*" OR "Pallas cat" OR "*Felis lynx*" OR "*Felis serval*" OR "*Leptailurus serval*" OR "*Felis temmincki*" OR "Asian golden cat" OR "golden cat" OR "*Felis* *viverrinus*" OR "fishing cat" OR "*Oncifelis* geoffroyi" OR "Geoffroy’s cat" OR "*Oncifelis colocolo*" OR "Pampas cat" OR "*Leopardus* *tigrinus*" OR oncilla OR "*Leopardus wiedii*" OR margay OR "*Herpailurus yagouaroundi*" OR jaguarundi OR "anti-*toxoplasma* antibody" OR soil) AND 1970/01/01:2019/12/31[dp]  Syntax for Web of Science  (ALL=(Toxoplasm*) OR ALL=(*Toxoplasma*) OR ALL=("*Toxoplasma gondii*") OR ALL=("*Toxoplasma gondius*") OR (ALL=(*Toxoplasma*) AND ALL=(*gondii*)) OR ALL=(toxoplasmosis) OR ALL=(toxoplasmoses) OR ALL=("*Toxoplasma* *gondii* infection") OR (ALL=(*"Toxoplasma* *gondii*") AND ALL=(infection)) OR ALL=("*Toxoplasma gondii*-like oocysts") OR ALL=("*Toxoplasma gondii* oocyst") OR (ALL=("*Toxoplasma gondii*") AND ALL=(oocyst))) AND (ALL=(cat) OR ALL=("domestic cat") OR (ALL=(cat) AND ALL=(domestic)) OR ALL=(*Felis*) OR ALL=("*Felis sylvestris catus*") OR ALL=("*Felis sylvestris gordoni*") OR ALL=("Gordon’s cat") OR ALL=("wild cat") OR ALL=("*Felis domesticus*") OR ALL=("*Felis domestica*") OR ALL=(caracal) OR ALL=("*Caracal caracal*") OR ALL=(*Uncia*) OR ALL=("*Uncia uncia*") OR ALL=(leopard) OR ALL=("snow leopard") OR (ALL=(snow) AND ALL=(leopard)) OR ALL=("panthera snow") OR ALL=("Panthera uncia") OR ALL=("clouded leopard") OR (ALL=(clouded) AND ALL=(leopard)) OR ALL=("*Neofelis nebulosa*") OR ALL=(ocelot) OR ALL=("Leopardus pardalis") OR ALL=(cheetah) OR ALL=("Acinonyx *jubatus*") OR ALL=(bobcat) OR ALL=("*Lynx* *rufus*") OR ALL=("*Lynx* *canadiensis*") OR ALL=("*Lynx* *lynx*") OR ALL=("*Lynx* *pardinus*") OR ALL=("Iberian lynx") OR ALL=(polecat) OR ALL=(jaguar) OR ALL=("Panthera onca") OR ALL=("*Panthera pardus*") OR ALL=("black panther") OR ALL=(lion) OR ALL=("*Panthera leo*") OR ALL=(tiger) OR ALL=("Panthera tigris") OR ALL=("*Panthera tigris altaica*") OR ALL=(puma) OR ALL=("Florida panther") OR ALL=("*Puma concolor coryi*") OR ALL=(cougar) OR ALL=("*Puma concolor*") OR ALL=("mountain lion") OR ALL=("*Felis concolor*") OR ALL=("*Felis concolor vancouverensis*") OR ALL=("*Felis chaus*") OR ALL=("*Felis euptilurus*") OR ALL=("Amur leopard cat") OR ALL=("*Felis margarita*") OR ALL=("sand cat") OR ALL=("*Felis manul*") OR ALL=("*Otocolobus manul*") OR ALL=("Pallas cat") OR ALL=("*Felis lynx*") OR ALL=("*Felis serval*") OR ALL=("*Leptailurus serval*") OR ALL=("*Felis temmincki*") OR ALL=("Asian golden cat") OR ALL=("golden cat") OR ALL=("*Felis* *viverrinus*") OR ALL=("fishing cat") OR ALL=("*Oncifelis* geoffroyi") OR ALL=("Geoffroy’s cat") OR ALL=("*Oncifelis colocolo*") OR ALL=("Pampas cat") OR ALL=("*Leopardus* *tigrinus*") OR ALL=(oncilla) OR ALL=("*Leopardus wiedii*") OR ALL=(margay) OR ALL=("*Herpailurus yagouaroundi*") OR ALL=(jaguarundi) OR ALL=("anti-*toxoplasma* antibody") OR ALL=(soil)) AND PY=1970-2019  Syntax for Scopus  (ALL(Toxoplasm*) OR ALL(*Toxoplasma*) OR ALL("*Toxoplasma gondii*") OR ALL("*Toxoplasma gondius*") OR (ALL(*Toxoplasma*) AND ALL(*gondii*)) OR ALL(toxoplasmosis) OR ALL(toxoplasmoses) OR ALL("*Toxoplasma* *gondii* infection") OR (ALL("*Toxoplasma* *gondii*") AND ALL(infection)) OR ALL("*Toxoplasma gondii*-like oocysts") OR ALL("*Toxoplasma gondii* oocyst") OR (ALL("*Toxoplasma gondii*") AND ALL(oocyst))) AND (ALL(cat) OR ALL("domestic cat") OR (ALL(cat) AND ALL(domestic)) OR ALL(*Felis*) OR ALL("*Felis sylvestris catus*") OR ALL("*Felis sylvestris gordoni*") OR ALL("Gordon’s cat") OR ALL("wild cat") OR ALL("*Felis domesticus*") OR ALL("*Felis domestica*") OR ALL(caracal) OR ALL("*Caracal caracal*") OR ALL(*Uncia*) OR ALL("*Uncia uncia*") OR ALL(leopard) OR ALL("snow leopard") OR (ALL(snow) AND ALL(leopard)) OR ALL("panthera snow") OR ALL("Panthera uncia") OR ALL("clouded leopard") OR (ALL(clouded) AND ALL(leopard)) OR ALL("*Neofelis nebulosa*") OR ALL(ocelot) OR ALL("Leopardus pardalis") OR ALL(cheetah) OR ALL("Acinonyx *jubatus*") OR ALL(bobcat) OR ALL("*Lynx* *rufus*") OR ALL("*Lynx* *canadiensis*") OR ALL("*Lynx* *lynx*") OR ALL("*Lynx* *pardinus*") OR ALL("Iberian lynx") OR ALL(polecat) OR ALL(jaguar) OR ALL("Panthera onca") OR ALL("*Panthera pardus*") OR ALL("black panther") OR ALL(lion) OR ALL("*Panthera leo*") OR ALL(tiger) OR ALL("Panthera tigris") OR ALL("*Panthera tigris altaica*") OR ALL(puma) OR ALL("Florida panther") OR ALL("*Puma concolor coryi*") OR ALL(cougar) OR ALL("*Puma concolor*") OR ALL("mountain lion") OR ALL("*Felis concolor*") OR ALL("*Felis concolor vancouverensis*") OR ALL("*Felis chaus*") OR ALL("*Felis euptilurus*") OR ALL("Amur leopard cat") OR ALL("*Felis margarita*") OR ALL("sand cat") OR ALL("*Felis manul*") OR ALL("*Otocolobus manul*") OR ALL("Pallas cat") OR ALL("*Felis lynx*") OR ALL("*Felis serval*") OR ALL("*Leptailurus serval*") OR ALL("*Felis temmincki*") OR ALL("Asian golden cat") OR ALL("golden cat") OR ALL("*Felis* *viverrinus*") OR ALL("fishing cat") OR ALL("*Oncifelis* geoffroyi") OR ALL("Geoffroy’s cat") OR ALL("*Oncifelis colocolo*") OR ALL("Pampas cat") OR ALL("*Leopardus* *tigrinus*") OR ALL(oncilla) OR ALL("*Leopardus wiedii*") OR ALL(margay) OR ALL("*Herpailurus yagouaroundi*") OR ALL(jaguarundi) OR ALL("anti-*toxoplasma* antibody") OR ALL(soil)) AND (PUBYEAR > 1969 AND PUBYEAR < 2020)  Syntax for **CABI**  (Cat) AND (Toxoplasma) AND yr:[1970 TO 2019] |
| --- |
