# Supplementary material for: Toxoplasma gondii infection in domestic and wild felids as public health concerns: a systematic review and meta-analysis
Source: Sci Rep. 2021 May 4;11:9509. doi: 10.1038/s41598-021-89031-8 (PMC8097069; doi:10.1038/s41598-021-89031-8)
Supplement: Supplementary file 4 — Supplementary Information 4. [file 41598_2021_89031_MOESM4_ESM.doc]

**Table S2.** Characteristics of the eligible studies based on the seroprevalence of anti-*Toxoplasma gondii* antibodies in domestic cats (*Felis domesticus*) (sorted by continent and publication date)

| **Location** | **Period** | **Stray / pet** | **Age groups** | **Test** | **Cut-off** | **Sample size** | **Positive (%)** | **Author and publication date** |
| --- | --- | --- | --- | --- | --- | --- | --- | --- |
| **AFRICA** |  |  |  |  |  |  |  |  |
| Egypt (Giza) | 1975 | Stray | ns | DT | ns | 376 | 149 (39.6) | Rifaat et al. 1976^e^ |
| Nigeria (Niger Delta) | 1983 | Pet† | >1y; <1y | DT | 1:10 | 200 | 188 (94.2) | Arene 1984 |
| Lebanon (Beirut) | 1980-1983 | Stray & pet | ns | IFA | ns | 324 | 253 (78.1) | Deeb et al. 1985 |
| Egypt (Cairo) | 1987 | Stray | ns | IFAT | 1:64 | 177 | 104 (58.8) | Aboul-Magd et al. 1988^a^ |
| Egypt (Gharbia) | 1988 | Stray & pet | ns | IHA | 1:64 | 114 | 21 (18.4) | Abu-Zakham et al. 1989^a^ |
| Guatemala (Peten) | 2004 | ns | ns | MAT | 1:32 | 30 | 16 (53.0) | Lickey et al. 2005 |
| Egypt (Giza) | 2008-2009 | Stray | ns | MAT | ≥1:25 | 158 | 154 (97.4) | Al-Kappany et al. 2010 |
| Nigeria (Maiduguri) | 2009 | Stray | ≤1; 1-5; >5y | LAT | ≥1:64 | 105 | 38 (36.2) | Kamani et al. 2010 |
| Egypt (Cairo) | 2010 | ns | ns | MAT | ≥1:5 | 180 | 172 (95.5) | Al-Kappany et al. 2011 |
| New Caledonia | 2009 | ns | Juveniles; adults | ELISA | ns | 8 | 4 (50.0) | Roqueplo et al. 2011 |
| South Africa (Johannesburg) | 2013 | Pet^e^ | 5-216 m | ELISA | ns | 102 | 18 (17.6) | Lobetti and Lappin 2012 |
| Ethiopia (Addis Ababa) | 2011 | Stray | ns | MAT | 1:25 | 36 | 33 (91.6) | Dubey et al. 2013 |
| Ethiopia (Addis-Ababa) | 2011 | Stray | ≥6m | MAT | ≥1:25 | 48 | 41 (85.4) | Tiao et al. 2013 |
| Angola (Luanda) | 2014-2016 | Pet | 2.5-143 m | MAT | ≥1:20 | 102 | 4 (3.9) | Lopes et al. 2017 |
| Nigeria (Ibadan) | 2014 | Pet | 1-36 m | MAT | 1:20 | 226 | 10 (4.4) | Ayinmode et al., 2017 |
| Algeria (Algiers) | 2016 | Stray | ns | MAT | 1:6 | 96 | 48 (50.0) | Yekkour et al. 2017 |
| **ASIA** |  |  |  |  |  |  |  |  |
| Japan (Kanto) | 1971 | Stray | ns | IFAT | ns | 90 | 40 (44.0) | Werner and Walton 1973 |
| Thailand (Bangkok) | 1980 | ns | ns | ns | 1:256 | 40 | 23 (57.5) | Sriwaranard et al. 1981^a^ |
| Japan (Kanto) | 1973-1981 | Stray | ns | IHA | 1:64 | 171 | 81 (47.1) | Fujinami et al. 1983^a^ |
| Japan (various areas) | 1988-1990 | Stray | ns | IFAT | 1:4 | 335 | 86 (25.7) | Oikawa et al. 1990^a^ |
| Taiwan | 1989 | Stray & pet^b^ | ns | KELA | ns | 117 | 9 (7.7) | Lin et al. 1990 |
| China (Guangdong) | 1988 | Stray | ns | IHA | 1:64 | 47 | 1 (2.1) | Lin et al. 1990 |
| China (Guangdong) | 1989 | ns | ns | IHA | 1:64 | 47 | 1 (2.1) | Shen et al. 1990 |
| Japan (Saitama) | 1992 | ns | ns | LAT | 1:32 | 726 | 76 (10.5) | Furuya et al. 1993^a^ |
| Singapore | ns | Stray | ns | LAT | 1:64 | 706 | 217 (30.7) | Chong et al. 1993^a^ |
| China (Shandong) | 1994 | ns | ns | IHA | 1:64 | 200 | 92 (46.0) | Fu et al. 1995^a^ |
| Turkey (Ankara) | 1995 | ns | ns | DA | 1:16 | 65 | 28 (43.0) | Inci et al. 1996^a^ |
| Japan (Hyogo) | 1990-1991 | Stray | ns | LAT | 1:64 | 231 | 44 (19.0) | Khin-Sane-Win et al. 1997^a^ |
| Bangladesh (Mymensingh) | 1995 | Stray | ns | LAT | 1:64 | 24 | 8 (33.3) | Samad et al. 1997 |
| China (Shanghai) | 1996 | ns | ns | IHA | 1:64 | 142 | 131 (92.2) | Lu et al. 1997^a^ |
| Taiwan | 1996 | ns | ns | LAT | 1:32 | 202 | 11 (5.5) | Tsai et al. 1997^a^ |
| Japan (various areas) | 1994-1995 | Pet^b^ | ns | LAT | 1:64 | 471 | 41 (8.7) | Maruyama et al. 1998 |

**Table 3. Continued**

| **Location** | **Period** | **Stray / pet** | **Age groups** | **Test** | **Cut-off** | **Sample size** | **Positive (%)** | **Author and publication date** |
| --- | --- | --- | --- | --- | --- | --- | --- | --- |
| Japan (various areas) | 1997 | Pet^b^ | ns | LAT | 1:32 | 800 | 48 (6.0) | Nogami et al. 1998 |
| South Korea (Chinju) | 1996-1998 | Stray | ns | WB | 1:200 | 198 | 26 (13.1) | Sohn and Nam 1999 |
| Japan (Chiba) | 1998-1999 | Stray | ns | LAT | 1:34 | 97 | 13 (13.4) | Hata et al. 2000 |
| China (Hubei) | 2000 | ns | ns | ELISA | ns | 105 | 33 (31.4) | Chen 2001^a^ |
| Japan (Saporo, Tokyo) | 2000 | ns | ns | ELISA | ns | 193 | 40 (20.7) | Kimbita et al. 2001 |
| Japan (Saitama) | 2001 | ns | ns | ELISA | 1:64 | 192 | 42 (21.9) | Huang et al. 2002 |
| Japan (Horraido, Okinawa) | 1994-1999 | Pet^b^ | 2m -15y | LAT | 1:64 | 1,447 | 78 (5.4) | Maruyama et al. 2003 |
| Thailand (Bangkok) | 2002 | Pet | ns | DA | 1:16 | 315 | 23 (7.3) | Sukthana et al. 2003 |
| Turkey (Van) | 2002 | Pet | ns | IHA | 1:64 | 62 | 57 (8.0) | Tutuncu et al. 2003 |
| China (Hebei) | 2004 | ns | ns | ELISA | ns | 75 | 43 (57.3) | Yuan et al. 2004^a^ |
| Israel (Jerusalem) | 1999-2000 | Stray | >5 y | ELISA | 1:80 | 1,062 | 178 (16.8) | Salant and Spira 2004 |
| Japan (Saitama) | 2003 | ns | ns | LAT | 1:64 | 179 | 33 (18.4) | Huang et al. 2004 |
| Mongolia | 2000-2001 | Stray | ns | ELISA | 1:64 | 15 | 0 (0.0) | Brown et al. 2005 |
| China (Guangzhou) | 2004 | ns | ns | ELISA | ns | 114 | 27 (23.7) | Chen et al. 2005^a^ |
| Iran (Tehran) | 2002 | Stray & pet | <1; 2; 3; >3y | IFAT | 1:32 | 100 | 63 (63.0) | Haddadzadeh et al. 2006 |
| China (Beijing) | 2005 | ns | ≤1; 1-3; 3-6; >6y | ELISA | ns | 128 | 18 (14.1) | Yu et al. 2006^a^ |
| Pakistan (Lahore) | 2005 | Stray & pet | ≤0.6; 0.7-1; 1-6; ≥7y | LAT | 1:16 | 50 | 28 (56.0) | Shahzad et al. 2006 |
| Thailand (Bangkok) | 2001-2002 | Stray | ns | LAT | 1:64 | 592 | 65 (11.0) | Jittapalapong et al. 2007 |
| Iran (Kashan) | 2004-2005 | Stray | ns | IFAT | 1:20 | 50 | 43 (86.0) | Hooshyar et al. 2007 |
| Iran (Tehran) | 2005 | Stray | ns | LAT | 1:8 | 4 | 2 (50.0) | Zia-Ali et al. 2007 |
| China (Guangzhou) | 2006 | Pet^f^ | ns | MAT | 1:20 | 34 | 27 (79.4) | Dubey et al. 2007 |
| China (Beijing) | 1999-2005 | Pet | <1; 1-8y | ELISA | ns | 335 | 50 (14.9) | Yu et al. 2008 |
| Turkey (Nigde) | 2003 | Stray | ns | DT | ≥1:16 | 72 | 55 (76.4) | Karatepe et al. 2008 |
| South Korea (various areas) | 2005-2006 | Stray | Unknown | PCR | - | 106 | 50 (47.2) | Lee et al. 2008 |
| Malaysia (Perak) | 2007 | Pet^b^ | ns | IFAT | 1:200 | 55 | 8 (14.5) | Chandrawathani et al. 2008 |
| South Korea (various areas) | 2007 | Stray | ns | LAT | 1:32 | 174 | 14 (8.1) | Kim et al. 2008 |
| Turkey (Ankara) | 2007 | Pet^b^ | <2; 1-2; >2y | DT | 1:4 | 99 | 40 (40.3) | Ozkan et al. 2008 |
| China (Guangzhou) | 2007 | Stray & pet | ns | ELISA | ns | 206 | 52 (25.2) | Zhang et al. 2009 |
| Iran (Sari) | 2004 | Stray | ns | LAT | ≥1:64 | 100 | 40 (40.0) | Sharif et al. 2009 |
| Vietnam (Thua Thien Hue) | 2007 | Stray | ns | LAT | 1:64 | 155 | 112 (72.3) | Hosono et al. 2009 |
| Iran (Kerman) | 2008 | Stray | ns | MAT | ≥1:25 | 70 | 31 (44.2) | Akhtardanesh et al. 2010 |
| Iran (Kerman) | 2008 | Pet | ns | MAT | ≥1:25 | 70 | 24 (34.2) | Akhtardanesh et al. 2010 |
| Thailand (Bangkok) | 2006 | Stray | <3; 3-5; >5y | DT | ≥1:16 | 1,490 | 72 (4.8) | Jittapalapong et al. 2010 |
| South Korea (Seoul) | 2008 | Stray & pet | ns | ELISA | ns | 152 | 11 (7.2) | Lee et al. 2010 |

**Table 3. Continued**

| **Location** | **Period** | **Stray / pet** | **Age groups** | **Test** | **Cut-off** | **Sample size** | **Positive (%)** | **Author and publication date** |
| --- | --- | --- | --- | --- | --- | --- | --- | --- |
| United Arab Emirates (Sharjah) | 2009 | Stray | ns | MAT | 1:25 | 4 | 1 (25.0) | Dubey et al. 2010 |
| South Korea (Seoul) | 2008 | Stray | ns | ELISA | ns | 456 | 69 (15.8) | Lee et al. 2011 |
| Iran (Urmia) | 2008-2010 | Stray & pet | ns | MAT | ≥1:20 | 130 | 46 (35.3) | Raeghi et al. 2011 |
| Iiran (Ahvaz) | 2006-2009 | Pet† | <6m; 6m-3y; >3y | TT | ns | 198 | 49 (24.7) | Mosallanejad et al. 2011 |
| Iran (Ahvaz) | 2009 | Stray | ns | MAT | ≥1:25 | 100 | 54 (54.0) | Hamidinejat et al. 2011 |
| Sri Lanka (Colombo) | 2010 | Stray & pet | <1; 1-2; 2-4; >16y | MAT | ≥1:25 | 86 | 26 (30.2) | Kulasena et al. 2011 |
| China (Lanzhou) | 2010-2011 | Stray & pet | <1; 2-3; ≥3 y | MAT | 1:25 | 221 | 47 (21.3) | Wu et al. 2011 |
| Thailand (Bangkok) | 2009 | Pet | ns | MAT | ≥1:25 | 348 | 35 (10.1) | Sukhumavasi et al. 2012 |
| China (Beijing) | 2009-2010 | Stray | ns | MAT | ≥1:20 | 60 | 37 (57.8) | Qian et al. 2012 |
| China (Shanghai) | 2010-2011 | Stray | ≤1; >1y | ELISA | ns | 145 | 17 (11.7) | Wang et al. 2012 |
| Iran (Garmsar) | 2007-2008 | Stray | ns | ELISA | ns | 107 | 69 (64.5) | Tehrani-Sharif et al. 2013 |
| Iraq (various areas) | 2008 | Stray | ns | LAT | ≥1:16 | 207 | 63 (30.4) | Switzer et al. 2013 |
| South Korea (Seoul) | 2009-2011 | Pet | <6m; 6m-5y; >5y | ELISA | ns | 437 | 10 (2.2) | Hong et al. 2013 |
| Kuwait | 2012 | Stray | <6; ≥6m | IHA | ns | 240 | 47 (19.6) | Abdou et al. 2013^a^ |
| Taiwan (Taipei) | 2008-2010 | Pet† | ns | ELISA | ns | 100 | 10 (10.0) | Fuh et al. 2013 |
| Thailand | 2007-2008 | Stray | ns | LAT | 1:256 | 36 | 3 (8.3) | Arunvipas et al. 2013 |
| Korea (Daejeon) | 2012 | Stray | ns | ELISA | 1:50 | 118 | 7 (5.93) | Park et al. 2014 |
| Turkey (Izmir) | 2010 | Pet | ns | IFAT | ≥1:16 | 1,121 | 384 (34.2) | Can et al. 2014 |
| Pakistan (various areas) | 2012 | Pet | <1; >1y | ELISA | ns | 420 | 111 (26.4) | Ahmad et al. 2014 |
| China (Zhenjiang) | 2013 | Stray & pet | >6y; <6y | ELISA |  | 116 | 24 (20.7) | Liu et al. 2014 |
| China (Guizhou) | 2011-2012 | Stray | ns | ELISA | ns | 19 | 16 (84.2) | Li et al. 2015 |
| China (Henan) | 2013-2014 | Pet | ns | MAT | ≥1:25 | 42 | 21 (50.0) | Yang et al. 2015 |
| Japan (Tokyo) | 1999-2011 | Pet | ns | LAT | 1:64 | 337 | 20 (5.9) | Oi et al. 2015 |
| China (various areas) | 2012-2015 | Stray & pet | <1; 1-3; >3y | IHA | ≥1:64 | 1,141 | 176 (15.4) | Kang et al. 2016 |
| China (Gansu) | 2014-2015 | Stray & pet | ns | MAT | ≥1:25 | 362 | 70 (19.3) | Cong et al. 2016 |
| India (Various area) | 2012 | Pet | 0-1y; 1-2y; 2-10y | ELISA | ns | 24 | 11 (45.83) | Shah et al. 2016 |
| Japan (Amami Oshima) | 2013-2017 | Stray | ns | GLIPS | - | 1,363 | 123 (9.0) | Matsuu et al. 2017 |
| China (Henan) | 2015-2016 | Pet | ≤1; 1-6; ≥6y | ELISA | ns | 843 | 178 (21.0) | Wang et al. 2017 |
| China (Henan) | 2015-2017 | Pet | ns | MAT | 1:25 | 28 | 2 (7.1) | Yang et al., 2017 |
| Korea (nine major cities) | 2016 | Stray & pet | 1-12 y | ELISA | ns | 150 | 6 (4.0) | Kim et al. 2017 |
| Korea (Various areas) | 2013-2015 | Feral | ns | ELISA | ns | 112 | 56 (50.0) | Hwang et al. 2017 |
| Qatar | 2014-2015 | Stray | ns | MAT | ≥1:25 | 495 | 406 (82.0) | Boughattas et al. 2017 |
| Iran (Ahvaz) | 2013 | Stray | <1y; 1-6y; >6y | MAT | 1:25 | 100 | 39 (39.0) | Mosallanejad et al. 2017 |
| China (Jiangsu) | 2011-2016 | Stray | ns | ELISA | ≥1:5 | 64 | 16 (25.0) | Hou et al. 2018 |

**Table 3. Continued**

| **Location** | **Period** | **Stray / pet** | **Age groups** | **Test** | **Cut-off** | **Sample size** | **Positive (%)** | **Author and publication date** |
| --- | --- | --- | --- | --- | --- | --- | --- | --- |
| Iran (Shiraz) | 2017 | Stray | ns | MAT | 1:20 | 29 | 24 (82.8) | Asgari et al. 2018 |
| Japan (Tokachi) | 2013-2014 | Pet | <1; 1-2; 2-3; >3 y | ELISA | ≥1:32 | 353 | 57 (16.1) | Salman et al. 2018 |
| Thailand (Bangkok) | 2015 | Stray | ≤1; 1-5; >5y | IFAT | ns | 458 | 41 (0.9) | Kengradomkij et al. 2018 |
| China (Shandong) | 2016-2017 | Pet | ≤1; 2; 3; >3y | IHA | ≥1:64 | 180 | 39 (21.6) | Cong et al. 2018 |
| Iran (Khorasan Razavi) | 2016 | Stray | <6m; 6m-3y; >3y | ELISA | ns | 159 | 94 (59.1) | Khodaverdi and Razmi 2019 |
| Saudi Arabia (Riyadh) | 2017 | Stray & pet | ns | ELISA | 1:40 | 200 | 52 (26.0) | Mohammed et al. 2019 |
| **AUSTRALIA** |  |  |  |  |  |  |  |  |
| Australia (Tasmania) | 1974 | Stray | ns | ns | ns | 53 | 51 (96.2) | Gregory and Munday 1976 |
| Australia (South-eastern) | 1980 | Feral | Adults | IHA | ns | 75 | 19 (20.0) | Coman et al. 1981 |
| Australia (Sydney) | 1980 | Pet^b^ | Kittens; adults | IHA | 1:64 | 80 | 42 (52.5) | Watson et al. 1982 |
| Australia (Tasmania) | 1996 | Feral | ns | LAT | 1:64 | 18 | 9 (50.0) | Milstein and Goldsmid 1997^a^ |
| Australia (Melbourne) | 1998 | Stray | ns | ELISA | ns | 103 | 40 (39.0) | Sumner and Ackland 1999^a^ |
| Australia (Tasmania) | 2012 | Stray | ns | MAT | ns | 266 | 224 (84.2) | Fancourt and Jackson 2014 |
| **EUROPE** |  |  |  |  |  |  |  |  |
| Germany (South) | 1978-1979 | ns | ns | IFAT | ns | 694 | 516 (74.4) | Boch and Walter 1979 |
| Scotland | 1878 | Stray & Pet | ns | DT | ns | 136 | 35 (25.7) | McColm et al. 1981 |
| Belgium (Antwerp) | 1984 | Pet | 1m-14y | DT | ns | 150 | 70 (47.0) | Beeck et al. 1985 |
| Scotland (Glasgow) | 1986 | Stray & Pet | <6m; >6m | DT | ns | 158 | 30 (19.0) | Jackson et al. 1987 |
| UK (South-west) | 1986 | ns | ns | ns | ns | 51 | 24 (47.0) | Gethings et al. 1987 |
| Czech Republic | 1987 | ns | ns | DT | 1:4 | 620 | 302 (48.7) | Svoboda et al. 1988^a^ |
| Germany | 1988 | ns | ns | DT | 1:16 | 267 | 160 (59.9) | Knaus and Fehler 1989^a^ |
| Sweden | 1989 | ns | ns | ELISA | ns | 244 | 102 (42.0) | Uggla et al. 1990^a^ |
| Germany (Lubeck) | 1989 | ns | ns | IFAT | ns | 318 | 158 (49.6) | Unbehauen 1991 |
| Sweden | 1993 | ns | ns | IFAT | 1:25 | 60 | 28 (46.6) | Ljungstrom et al. 1994 |
| Germany (Lubeck) | 1991 | ns | ns | ELISA | ns | 306 | 137 (45.0) | Tenter et al. 1994 |
| Austria | 1995 | ns | ns | IFA | 1:40 | 456 | 220 (48.2) | Edelhofer and Aspock 1996 |
| UK (Oxfordshire) | 1994 | FRFC | Adults | ELISA | 1:16 | 45 | 28 (62.0) | Yamaguchi et al. 1996 |
| Italy (Verona) | 1996 | Stray | 1<; 1-4; 5-8; >8y | MAT | 1:64 | 490 | 163 (33.3) | D’Amore et al. 1997 |
| Czech Republic (Brno) | 1995-1997 | Pet^b^ | 3m-16y | IFAT | ≥1:10 | 390 | 240 (61.5) | Svobodova et al. 1998 |
| Germany | 2000 | ns | ns | ELISA | ns | 300 | 197 (65.6) | Hecking-Veltman et al. 2001^a^ |
| Belgium (Ghent) | 1998-2000 | Stray | ns | MAT | 1:40 | 346 | 243 (70.2) | Dorny et al. 2002^a^ |
| Poland (South-western) | 2001 | Stray & pet | ns | LAT | 1:64 | 200 | 105 (52.5) | Smielewska-Los and Pacon 2002^a^ |
| Spain (Barcelona) | 2002 | Stray & pet | <1; ≥1y | MAT | 1:25 | 220 | 99 (45.0) | Gauss et al. 2003 |
| Poland (Olsztyn) | 2003 | Pet^b^ | ns | MAT | 1:20 | 17 | 12 (70.6) | Michalski and Platt-Samoraj 2004^a^ |

**Table 3. Continued**

| **Location** | **Period** | **Stray / pet** | **Age groups** | **Test** | **Cut-off** | **Sample size** | **Positive (%)** | **Author and publication date** |
| --- | --- | --- | --- | --- | --- | --- | --- | --- |
| Spain (various areas) | 2003 | Stray & pet | >6m; <6m | IFAT | ≥1:80 | 585 | 189 (32.3) | Miro et al. 2004 |
| France | 2005 | ns | ns | DAT | 1:64 | 50 | 20 (40.0) | Meunier et al. 2006 |
| France (Lyon) | 1993-2004 | Stray^c^ | ns | MAT | 1:40 | 301 | 56 (18.6) | Afonso et al. 2006 |
| Italy (Tuscany) | 2003-2004 | Stray | 6 m to 10 y | DAT | 1:4 | 573 | 233 (40.7) | Papini et al. 2006 |
| Czech Republic (Bohemia) | 2002-2006 | Pet^e^ | ns | IFAT | ≥1:40 | 286 | 126 (44.1) | Sedlak and Bartova 2006 |
| Slovak Republic | 2006 | ns | ns | ELISA | ns | 164 | 31 (18.9) | Ondrejka et al. 2007 |
| Italy (Veneto) | 2005-2006 | Stray | ns | IHA | 1:200 | 189 | 131 (69.3) | Natale et al. 2007 |
| Portugal (northeastern) | 2004-2005 | Pet | 2-11; 12-35; 36-180m | MAT | 1:20 | 204 | 73 (35.8) | Lopes et al. 2008 |
| Belgium (Brussels) | 2004-2006 | Pet | 3m-7y | IFAT | 1:50 | 567 | 141 (24.9) | de Craeye et al. 2008 |
| Belgium (Brussels) | 2004-2007 | Pet | 3m-8y | IFAT | 1:50 | 410 | 110 (26.8) | de Craeye et al. 2008 |
| Hungary (various areas) | 2007 | Stray | 2 - 13y | IFAT | 1:20 | 330 | 157 (47.6) | Hornok et al. 2008 |
| Spain (various areas) | 2007 | Stray & pet | ns | IFAT | 1:80 | 592 | 103 (17.3) | Montoya et al. 2008 |
| Spain (Andalusia) | 2004-2006 | Stray | ns | MAT | 1:25 | 25 | 13 (50.2) | Millan et al. 2009 |
| Italy (Florence) | 2009 | Pet | 6-24 m | MAT | ≥1:20 | 50 | 22 (44.0) | Mancianti et al. 2010 |
| Ireland | 2009 | Stray & pet | ns | ELISA | ns | 83 | 28 (33.7) | Juvet et al. 2010 |
| Poland (Olsztyn) | 2009 | Pet^b^ | ns | DAT | 1:40 | 135 | 89 (65.9) | Michalski et al. 2010^a^ |
| Romania (Cluj-Napoca) | 2007-2010 | Pet | 1m-17y | ELISA | ns | 236 | 111 (47.0) | Gyorke et al. 2011 |
| Romania (Arad) | 2010 | ns | ns | ELISA | ns | 36 | 29 (80.5) | Hotea et al. 2011 |
| Romania (Timişoara Zoo) | 2009-2010 | Captive | ns | ELISA | ns | 1 | 1 (100) | Darabus et al. 2011 |
| Saudi Arabia (Al-Ahsa) | 2010 | Stray & pet | ns | ELISA | ns | 156 | 98 (62.8) | Al-Mohammed 2011 |
| Scotland (Edinburg) | 2009 | Pet^b^ | <1; 1-12; ≥13y | ELISA | ≥1:64 | 52 | 10 (19.2) | Bennett et al. 2011 |
| Italy (Lombardy) | 2008-2010 | Stray | ns | IFAT | ns | 203 | 62 (30.5) | Spada et al. 2012 |
| Netherland | 2005-2010 | Pet^b^ | ≤1.5; 1.5-10; >10y | ELISA | ns | 450 | 91 (20.2) | Opsteegh et al. 2012 |
| Finland (Helsinki) | 2008-2009 | Pet | ns | DAT | ≥1:40 | 490 | 237 (48.4) | Jokelainen et al. 2012 |
| Portugal (Lisbon) | 2009-2010 | Stray | ns | DAT | ≥1:20 | 423 | 187 (44.2) | Waap et al. 2012 |
| Italy (Milan) | 2011 | Stray | ns | IFAT | ns | 139 | 70 (50.3) | Spada et al., 2013 |
| Romania (Western) | 2009 | Stray & pet | ns | ELISA | ns | 605 | 361 (42.5) | Hotea et al. 2013 |
| Lativa (various areas) | 2011-2012 | Pet^b^ | <1; 1-6; >7y | ELISA | ns | 242 | 125 (51.6) | Deksne et al. 2013 |
| Albania (Tirana) | 2008-2010 | Pet | ns | ELISA | ≥1:100 | 146 | 91 (62.3) | Silaghi et al. 2014 |
| Portugal (Lisbon) | 2007-2008 | Pet | 6-12; 12-24m | MAT | ≥1:40 | 215 | 44 (20.5) | Esteves et al. 2014 |
| Albania (Southwest) | 2012-2014 | Pet† | ns | MAT | 1:25 | 138 | 58 (42.0) | Lamaj et al. 2015 |
| Estonia | 2013 | Pet | <1; ≥1 | DAT | 1:40 | 306 | 193 (63.1) | Must et al. 2015 |
| Estonia | 2013 | Shelter | <1; ≥1 | DAT | 1:40 | 184 | 105 (57.1) | Must et al. 2015 |
| Portugal (Southern) | 2014 | Stray | ns | MAT | 1:20 | 79 | 31 (39.2) | Waap et al. 2016 |

**Table 3. Continued**

| **Location** | **Period** | **Stray / pet** | **Age groups** | **Test** | **Cut-off** | **Sample size** | **Positive (%)** | **Author and publication date** |
| --- | --- | --- | --- | --- | --- | --- | --- | --- |
| Italy (Perugia) | 2014-2015 | Pet | ns | IFAT | 1:64 | 78 | 33 (42.3) | Veronesi et al. 2017 |
| Finland | 2015 | Pet | Adult; kitten | DAT | ≥1:40 | 1,121 | 461 (41.1) | Must et al. 2017 |
| Romania (Timis) | 2015 | Pet | ns | ELISA | ns | 144 | 106 (73.6) | Hotea et al. 2017 |
| Russia (Kazan) | 2013 | Pet^b^ | ns | ELISA | ≥1:16 | 99 | 39 (39.4) | Shuralev et al. 2018 |
| Spain (Central) | 2014-2017 | Stray | ≤1; >1y | DAT | ≥1:80 | 356 | 86 (24.2) | Montoya et al. 2018 |
| France (North eastern) | 2014-2016 | Stray | ns | MAT | ≥1:25 | 130 | 38 (29.2) | Simon et al. 2018 |
| Poland (Silesian) | 2017 | ns | 0.5-12y | IFAT | 1:128 | 208 | 143 (66.3) | Sroka et al. 2018 |
| **NORTH AMERICA** |  |  |  |  |  |  |  |  |
| USA (New Mexico) | 1975 | Pet | ns | DT | ≥1:32 | 91 | 7 (8.0) | Marchiondo et al. 1976 |
| USA (California) | 1975 | Pet | ns | IHA | ns | 47 | 18 (38.0) | Franti et al. 1976 |
| USA (Ohio) | 1975 | Stray | ns | IFAT | ns | 1,000 | 390 (39.0) | Claus et al. 1977^a^ |
| Canada (Ontario) | 1976 | Pet | ns | DT | ns | 152 | 30 (19.7) | Tizard et al. 1978 |
| USA (California) | 1973 | Pet | ns | IHA | 1:64 | 86 | 17 (20.0) | Riemann et al. 1978 |
| USA (Washington) | 1981 | Stray & pet | ns | ELISA | ns | 87 | 27 (31.0) | Ladiges et al. 1982^a^ |
| USA (Maryland) | 1984 | Stray | ns | IFAT | ns | 650 | 109 (14.5) | Childs and Seegar 1986 |
| USA (Maryland) | 1980-1981 | ns | ns | IFAT | 1:32 | 585 | 89 (15.2) | Witt et al. 1989^a^ |
| USA (Georgia) | 1988 | Pet^c^ | ns | ELISA | ≥1:64 | 188 | 114 (60.7) | Lappin et al. 1989 |
| USA (Oklahoma) | 1987-1988 | Pet^e^ | <1; 1-5; ≥6y | LAT | 1:16 | 618 | 136 (22.0) | Rodgers and Baldwin 1990 |
| USA (Florida, Georgia, Ohio) | 1991 | ns | ns | ELISA | ns | 124 | 92 (74.2) | Lappin et al. 1992^a^ |
| USA (Iowa) | 1991 | Stray | ns | MAT | 1:32 | 74 | 31 (41.9) | Smith et al. 1992 |
| USA (Illinois) | 1992-1993 | Stray | ns | MAT | ≥1:25 | 391 | 267 (68.3) | Dubey et al. 1995 |
| Canada (Ontario) | 1991-1993 | Pet^b^ | ns | IHA | 1:64 | 25 | 3 (12.0) | Quesnel et al. 1997^a^ |
| USA (Iowa) | 1984-1988 | Stray | ns | MAT | 1:32 | 20 | 16 (80.0) | Hill et al. 1998 |
| Canada (Victoria) | 1997 | Pet^†^ | ~ 8.7 y | MAT | 1:25 | 73 | 16 (21.9) | Aramini et al., 1999 |
| USA (Colorado) | 1993-1995 | Pet | <1; 1-10; >10y | ELISA | 1:64 | 206 | 49 (23.6) | Hill et al. 2000 |
| USA (Rhode Island) | 2001 | Stray | ns | MAT | 1:25 | 200 | 84 (42.0) | DeFeo et al. 2002^a^ |
| USA (Ohio) | 2001 | Stray | ns | MAT | ≥1:25 | 275 | 133 (48.0) | Dubey et al. 2002 |
| USA (Florida) | 1999-2000 | Stray | ns | ELISA | ns | 553 | 60 (10.8) | Luria et al. 2004 |
| USA (North Carolina) | 2003 | Stray & pet | 3m-19y | MAT | 1:25 | 176 | 89 (50.6) | Nutter et al. 2004 |
| USA (Nationwide) | 2004 | Pet^b^ | ns | ELISA | ns | 12,628 | 3991 (31.6) | Vollaire et al. 2005^a^ |
| USA (Hawaii) | 2002-2004 | Stray | ns | ELISA | 1:64 | 67 | 25 (37.3) | Danner et al. 2007 |
| USA (California) | 2003-2005 | Pet | <6; 6-12; >12m | ELISA | ≥1:64 | 194 | 47 (24.2) | Dabritz et al. 2007 |
| USA (California) | 2003-2005 | Pet | <6; 6-12; >12m | IFA | 1:16 | 194 | 70 (36.1) | Dabritz et al. 2007 |
| USA (California) | 2000-2005 | ns | ns | IFA | 1:320 | 5 | 3 (60.0) | Miller et al. 2008 |

**Table 3. Continued**

| **Location** | **Period** | **Stray/Pet** | **Age groups** | **Test** | **Cut-off** | **Sample size** | **Positive (%)** | **Author and publication date** |
| --- | --- | --- | --- | --- | --- | --- | --- | --- |
| USA (Midwestern zoos) | 2003-2005 | Stray | ns | MAT | 1:25 | 34 | 10 (29.4) | de Camps et al. 2008 |
| USA (Pennsylvania) | 2008 | Stray | 3w-8y | MAT | ≥1:25 | 210 | 41 (19.5) | Dubey et al. 2009 |
| Canada (Prince Edward) | 2009 | Stray | Juveniles; adults | ELISA | 1:256 | 94 | 28 (29.8) | Stojanovic and Foley 2011 |
| USA (Ilinois) | 2008-2009 | Feral | ns | IFAT | 1:25 | 18 | 6 (33.0) | Fredebaugh et al. 2011 |
| USA (Virginia) | 2007 | ns | ns | IFAT | ns | 232 | 63 (27.1) | Hsu et al. 2011 |
| USA (California, Colorado) | 2000-2010 | Stray | ns | ELISA | 1:52 | 272 | 27 (1.0) | Bevins et al. 2012 |
| USA (California) | 2006-2009 | Feral | Juvenile vs. adults | IFAT | ns | 736 | 137 (18.6) | VanWormer et al. 2013 |
| USA (Ohio) | 2010 | Stray | ns | MAT | 1:25 | 200 | 103 (51.5) | Ballash et al. 2014 |
| USA (Minnesota) | 2014-2015 | Stray | Juveniles; adults | MAT | 1:25 | 20 | 9 (45.0) | Verna et al. 2016 |
| USA (South Dakota) | 2015 | ns | ns | ELISA | ns | 32 | 2 (6.0) | Scorza and Lappin 2017 |
| USA (Iowa) | 2015-2016 | Stray | <6; 7-72; >72 m | ELISA | ns | 140 | 42 (30.0) | Palerme et al. 2018 |
| **CENTRAL/SOUTH AME.** |  |  |  |  |  |  |  |  |
| Hawaii | 1972 | Stray | ns | ns | ≥1:4 | 1,568 | 220 (14.0) | Wallace 1973^e^ |
| Costa Rica (various areas) | 1979 | ns | ns | ns | ns | 237 | 109 (46.0) | Ruiz and Frenkel 1980^e^ |
| Brazil (São Paulo) | 1984 | Stray | ns | IFA | ≥1:8 | 9 | 0 (0.0) | Salata et al. 1985 |
| Chile | 1988 | ns | ns | IHA | 1:16 | 27 | 23 (85.2) | Stutzin et al. 1989 |
| Argentina (Buenos Aires) | 1994 | Pet | 14 - 126 m | IHA | 1:32 | 169 | 33 (19.5) | Fernandez et al. 1995 |
| Argentina | 1994 | ns | ns | IFA | 1:8 | 68 | 17 (25.0) | Venturini et al. 1995^a^ |
| Panama (Panama City) | 1994 | ns | ns | MAT | 1:1 | 241 | 110 (45.6) | Frenkel et al. 1995^a^ |
| Argentina | 1996 | ns | ns | IFA | 1:8 | 100 | 27 (27.0) | Venturini et al. 1997^a^ |
| Colombia | 1997 | ns | ns | IFA | ns | 28 | 25 (89.3) | Londono et al. 1998 |
| Brazil (Rio de Janeiro) | 1996-1997 | Pet^b^ | <6m; 7m-12y; >12y | IFAT | 1:16 | 248 | 44 (17.7) | Lucas et al. 1999 |
| Brazil (Amazon) | 1998 | ns | ns | IFAT | 1:16 | 163 | 119 (73.0) | Garcia et al. 1999^a^ |
| Mexico (Guadalajara) | 1998 | Pet | ns | ELISA | ns | 24 | 17 (70.8) | Galvan Ramirez et al. 1999 |
| Chile (Valdivia) | 1999 | Stray | ns | IFA | >1:4 | 97 | 32 (33.0) | Ovalle et al. 2000^a^ |
| Brazil (São Paulo) | 2000 | Stray | ns | IFAT | ≥1:16 | 191 | 37 (19.4) | Langoni et al. 2001 |
| Brazil (São Paulo) | 2000 | ns | ns | MAT | 1:20 | 502 | 132 (26.3) | da Silva et al. 2002 |
| Brazil (São Paulo) | 2001 | ns | ns | MAT | 1:16 | 100 | 19 (19.0) | da Silva et al. 2002 |
| Brazil (Parana) | 2002 | ns | ns | IHA | ns | 41 | 10 (24.4) | Netto et al. 2003 |
| Brazil (Parana) | 2003 | ns | ns | MAT | 1:20 | 58 | 49 (84.4) | Dubey et al. 2004 |
| Brazil (São Paulo) | 2003 | ns | ns | ELISA | ns | 100 | 40 (40.0) | Meireles et al. 2004 |
| Brazil (São Paulo) | 2004 | Stray | ns | IFAT | ns | 28 | 15 (53.6) | Ortolani et al. 2005 |
| Mexico (Mexico city) | 1996-1997 | Feral | ns | ns | ns | 6 | 4 (66.6) | Suzan and Ceballos 2005 |
| Brazil (São Paulo) | 2005 | ns | ns | MAT | 1:25 | 237 | 85 (35.5) | Pena et al. 2006 |

**Table 3. Continued**

| **Location** | **Period** | **Stray / pet** | **Age groups** | **Test** | **Cut-off** | **Sample size** | **Positive (%)** | **Author and publication date** |
| --- | --- | --- | --- | --- | --- | --- | --- | --- |
| Colombia | 2005 | Stray | ns | MAT | ≥1:5 | 170 | 77 (45.2) | Dubey et al. 2006 |
| Brazil (Rondônia) | 2005 | Stray | ns | MAT | 1:25 | 63 | 55 (87.3) | Cavalcante et al. 2006^a^ |
| Grenada | 2005 | Pet | ns | MAT | 1:25 | 40 | 16 (35.0) | Asthana et al. 2006 |
| Kerguelen Islands | 2005 | ns | ns | MAT | 1:40 | 276 | 141 (51.1) | Afonso et al. 2007 |
| Puerto Rico (Mona Island) | 2004-2005 | Stray | Adult; kitten | MAT | ≥1:10 | 19 | 16 (84.2) | Dubey et al. 2007 |
| Mexico (Colima) | 2006 | Pet | ns | ELISA | ns | 80 | 23 (28.8) | Garcia-Marquez et al. 2007 |
| Mexico (Durango) | 2006 | Stray | <0.5; 0.5-1; >1y | MAT | 1:25 | 105 | 22 (21.0) | Alvarado-Esquivel et al. 2007 |
| West Indies (St. Kitts) | 2006 | Pet | ns | MAT | ≥1:25 | 106 | 90 (84.9) | Moura et al. 2007^a^ |
| Brazil (Rio de Janeiro) | 2002-2004 | Stray | ns | IHA | 1:16 | 118 | 85 (72.0) | Mendes-de-Almeida et al. 2007 |
| Galapagos (Isabela Island) | 2004 | Stray & pet | >12w | ELISA | 1:64 | 52 | 33 (63.0) | Levy et al. 2008 |
| Mexico (Mexico City) | 2007 | Pet | <1; 1-9; >9y | ELISA | ns | 169 | 37 (21.8) | Besne-Merida et al. 2008 |
| West Indies (Grenada) | 2004-2007 | Stray & pet | ns | MAT | ≥1:25 | 176 | 51 (28.9) | Dubey et al. 2009 |
| Mexico (Durango) | 2007 | Stray & pet | 0.3-10 y | MAT | 1:25 | 150 | 14 (9.3) | Dubey et al. 2009 |
| West Indies (St. Kitts) | 2008 | Stray | <6 m; adults | MAT | ≥1:10 | 96 | 71 (73.9) | Dubey et al. 2009 |
| Brazil (Santa Catarina) | 2008-2009 | Pet | ns | IFAT | ≥1:64 | 300 | 43 (14.3) | Rosa et al. 2010 |
| Brazil (Curitiba) | 2007 | Pet^b^ | Young; adult; old | IFAT | ≥1:16 | 282 | 46 (16.3) | Cruz et al. 2011 |
| Brazil (São Paulo) | 2010 | ns | Young; adult | IFAT | ≥1:64 | 70 | 11 (15.7) | Coelho et al. 2011 |
| USA (Various area) | 2005 | ns | <7m; ≥7m | ns | ns | 55 | 13 (23.6) | Levy et al. 2011 |
| Brazil (Maranhão) | 2008-2009 | Stray | ns | IFAT | ≥1:40 | 200 | 101 (50.5) | Braga et al. 2012 |
| Brazil (São Paulo) | 2011 | Stray | ns | IFAT | 1:16 | 251 | 51 (20.3) | Sobrinho et al. 2012 |
| Mexico (Yucatan) | 2011 | Pet | ≤1y; 1-7y | ELISA | ns | 220 | 202 (91.8) | Castillo-Morales et al. 2012 |
| Mexico (Yucatan) | 2011 | Stray | ns | ELISA | 1:1024 | 50 | 50 (100) | Jimenez-Coello et al. 2013 |
| Brazil (São Paulo) | 2010 | Stray | 1m-1y | IFAT | ≥1:64 | 386 | 63 (16.3) | Cardia et al. 2013 |
| Brazil (Mato Grosso do Sul) | 2007 | ns | ns | IFAT | ≥1:16 | 50 | 2 (4.0) | Braga et al. 2014 |
| Brazil (Mato Grosso do Sul) | 2013 | Stray & pet | ns | IFAT | ≥1:40 | 151 | 49 (32.5) | Sousa et al. 2014 |
| Brazil (Patos) | 2011 | Stray & pet | ns | IFAT | 1:16 | 201 | 88 (43.8) | Feitosa et al. 2014 |
| Peru (Lima) | 2013 | Pet | <1; 1-7; >7y | IHA | ns | 154 | 17 (11.0) | Cerro et al. 2014 |
| Mexico (various areas) | 2008 | Stray & pet | ns | ELISA | ns | 48 | 14 (29.2) | Rico-Torres et al. 2015 |
| Brazil (various areas) | 2008-2010 | Pet | ns | IFAT | ≥1:25 | 29 | 24 (82.7) | Furtado et al. 2015 |
| Brazil (Parana) | 2013 | ns ^b^ | ns | IFAT | ns | 171 | 47 (28.0) | Andrade et al. 2015^a^ |
| Brazil (Parana) | 2004-2012 | ns | ns | IFAT | ≥1:100 | 415 | 87 (20.9) | Caldart et al. 2015 |
| Brazil (Acre) | 2014 | ns | ns | IIF | ns | 89 | 22 (24.7) | Souza et al. 2015^a^ |
| Brazil (Northeastern) | 2015-2017 | Stray | 6-12m; 1-3y; >3y | IFAT | 1:64 | 35 | 9 (25.7) | Arraes-Santos et al. 2016 |
|  |  |  |  |  |  |  |  |  |

**Table 3. Continued**

| **Location** | **Period** | **Stray / pet** | **Age groups** | **Test** | **Cut-off** | **Sample size** | **Positive (%)** | **Author and publication date** |
| --- | --- | --- | --- | --- | --- | --- | --- | --- |
| Brazil (Fernando de Noronha) | 2015 | Stray & pet | ns | IFAT | 1:16 | 595 | 398 (66.9) | Magalhaes et al. 2017 |
| Brazil (Rio de Janeiro) | 2014-2015 | Stray & pet | Adult; juvenile | MAT | ≥1:20 | 372 | 36 (9.7) | Bolais et al. 2017 |
| Brazil (Parana) | 2016 | Pet | ns | IIF | ns | 100 | 29 (29.0) | Souza et al. 2017^a^ |
| Panama (Panama city) | 2015-2016 | Pet | ns | ELISA | ns | 120 | 30 (25.0) | Rengifo-Herrera et al. 2017 |
| Chile (Southern) | 2009-2013 | Stray | ns | MAT | ≥1:25 | 65 | 44 (67.7) | Barros et al. 2018 |

^a^ Abstract; DT, Dye test; ELISA, Enzyme-linked immunosorbent assay; IFAT, Indirect fluorescent antibody; IHA, Indirect hemagglutination; LAT, Latex agglutination test; MAT, Modified agglutination test; DAT, Direct Agglutination Test; WB, Western blot; ^b^ Samples from small animal hospitals and clinics; ^d^ A private small animal practice and at the local animal shelter of the town of Lubeck; ^e^ Samples submitted to a private veterinary laboratory; ^f^ Bought Changban Free Market, Guangzhou, Guangdong Province; KELA, Kinetics-based enzyme-linked immunosorbent; GLIPS, Gaussia luciferase immunoprecipitation system; † Cats brought to a veterinary clinic; ns, Not specified; ○ Breeding Centre for Endangered Arabian Wildlife; TT, Toxo Ab Test Kit; IIF, Indirect Immunofluorescence; FRFC, Free ranging farm cats

**References**

1. Barros M, Cabezon O, Dubey JP, Almeroa S, Ribas MP, Escobar LE, Ramos B, Medina-Vogel G. *Toxoplasma gondii* infection in wild mustelids and cats across an urban-rural gradient. PLoS ONE 13(6):e0199085
2. Bolais PF, Vignoles P, Pereira PF, Keim R, Aroussi A, Ismail K, Dardé ML, Amendoeira MR, Mercier A. *Toxoplasma gondii* survey in cats from two environments of the city of Rio de Janeiro, Brazil by Modified Agglutination Test on sera and filter-paper. Parasites & Vectors 2017;10:88
3. Rengifo-Herrera C, Pile E, García A, Pérez A, Pérez D, Nguyen FK, de la Guardia V, Mcleod R, Caballero Z. Seroprevalence of *Toxoplasma gondii* in domestic pets from metropolitan regions of Panama. Parasite 2017, 24, 9
4. Furtado MM, Gennari SM, Ikuta CY, de Almeida Jácomo AT, de Morais ZM, de Jesus Pena HF, de Oliveira Porfírio GE, Silveira L, Sollmann R, de Souza GO, Tôrres NM, Neto JSF. Serosurvey of Smooth Brucella, *Leptospira* spp. and *Toxoplasma gondii* in Free-Ranging Jaguars (*Panthera onca*) and Domestic Animals from Brazil. PLoS ONE 10(11): e0143816
5. Magalhaes FJR, Ribeiro-Andrade M, Souza FM, Filho CDFL, Biondo AW, Vidotto O, Navarro IT, Mota RA. Seroprevalence and spatial distribution of *Toxoplasma gondii* infection in cats, dogs, pigs and equines of the Fernando de Noronha Island, Brazil. Parasitol Int. 2017;66(2):43-46.
6. Rico-Torres CP, Del Viento-Camacho A, Caballero- Ortega H, Besné-Mérida A, Luna-Pastén H, Correa D, Palma-García JM. First isolation of *Toxoplasma gondii* from cats of Colima, Mexico: tissue distribution and genetic characterization. Vet Parasitol. 2015; 209(1-2):125-8.
7. Cerro L, Alicia Rubio A, Pinedo R, Mendes-de-Almeida F, Brener B, Labarthe N. Seroprevalence of *Toxoplasma gondii* in cats (*Felis catus*, Linnaeus 1758) living in Lima, Peru. Braz. J. Vet. Parasitol. 2014;23(1):90-93
8. de Sousa KCM, Herrera HM, Domingos IH, Campos JBV, dos Santos IMC, Neves HH, Machado RZ, Andre MR. Serological detection of *Toxoplasma gondii, Leishmania infantum* and *Neospora caninum* in cats from an area endemic for leishmaniasis in Brazil. Braz. J. Vet. Parasitol. 2014;23(4):449-455
9. Cardia DFF, Camossi LG, Neto LS, Langoni H, Bresciani KDS. Prevalence of *Toxoplasma gondii* and *Leishmania* spp. infection in cats from Brazil. Veterinary Parasitology 2013;197:634-637
10. Braga ARC, Corrêa APFL, Camossi LG, da Silva RC, Helio Langoni H, Lucheis SB . Coinfection by *Toxoplasma gondii* and *Leishmania* spp. in domestic cats (*Felis catus*) in State of Mato Grosso do Sul. Revista da Sociedade Brasileira de Medicina Tropical 2014;47(6):796-797
11. Castillo-Morales VJ, Viana KYA, Guzman-Marın ES, Jimenez-Coello M, Segura Correa JC, Aguilar-Caballero AJ, Ortega-Pacheco A. Prevalence and Risk Factors of *Toxoplasma gondii* Infection in Domestic Cats from the Tropics of Mexico Using Serological and Molecular Tests. Interdisciplinary Perspectives on Infectious Diseases 2012; Article ID 529108
12. Sobrinho LSV, Rossi CN, Vides JP, Braga ET, Gomes AAD, de Lima VMF, Perri SHV, Generoso D, Langoni H, Leutenegger C, Biondo AW, Laurenti MD, Marcondes M. Coinfection of *Leishmania chagasi* with *Toxoplasma gondii*, Feline Immunodeficiency Virus (FIV) and Feline Leukemia Virus (FeLV) in cats from an endemic area of zoonotic visceral leishmaniasis. Veterinary Parasitology 2012;187:302-306
13. Braga M, André M, Jusi M, Freschi CR, Teixeira M, Machado R. Occurrence of anti-*Toxoplasma gondii* and anti-*Neospora caninum* antibodies in cats with outdoor access in São Luís, Maranhão, Brazil. Rev. Bras. Parasitol. Vet. 2012; 21(2): 107-111
14. Rosa LD, Moura AB, Trevisani N, Medeiros AP, Sartor AA, de Souza AP, Bellato V. *Toxoplasma gondii* antibodies on domiciled cats from Lages municipality, Santa Catarina State, Brazil. Rev. Bras. Parasitol. Vet 2010; 19(4):268-269
15. Coelho WMD, do Amarante AFT, Apolinário JDC, Coelho NMD, de Lima VMF, Perri SHV, Bresciani KDS. Seroepidemiology of *Toxoplasma gondii*, *Neospora caninum*, and *Leishmania* spp. infections and risk factors for cats from Brazil. Parasitol Res 2011;109:1009-1013
16. Cruz MA, Ullmann LS, Montaño PY, Hoffmann JL, Langoni H, Biondo AW. Seroprevalence of *Toxoplasma gondii* infection in cats from Curitiba, Paraná, Brazil. Rev. Bras. Parasitol. Vet. 2011; 20(3):256-258
17. Palerme JS, Lamperelli E, Gagne J, Cazlan C, Zhang M, Olds JE. Seroprevalence of *Leptospira* spp., *Toxoplasma gondii*, and *Dirofilaria immitis* in Free-Roaming Cats in Iowa. Vector Borne and Zoonotic Diseases. 2018; 19(3):193-8.
18. Rifaat, M.A., Arafa, M.S., Sadek, M.S., Nasr, N.T., Azab, M.E., Mahmoud Khalil, M.S., 1976. *Toxoplasma* infection of stray cats in Egypt. J. Trop. Med. Hyg. 79, 67–70.
19. Ballash GA, Dubey JP, Kwok OCH., Shoben AB, Robison TL, Kraft TJ, Dennis PM. Seroprevalence of *Toxoplasma gondii* in White-Tailed Deer (*Odocoileus virginianus*) and Free-Roaming Cats (*Felis catus*) Across a Suburban to Urban Gradient in Northeastern Ohio. EcoHealth 2014
20. Hsu V, Grant DC, Zajac AM, Witonsky SG, Lindsay DS. Prevalence of IgG antibodies to *Encephalitozoon cuniculi* and *Toxoplasma gondii* in cats with and without chronic kidney disease from Virginia. Veterinary Parasitology 2011;176:23-26
21. Sævik BK, Krontveit RI, Eggen KP, Malmberg N, Thoresen SI, Prestrud KW. *Toxoplasma gondii* seroprevalence in pet cats in Norway and risk factors for seropositivity. J Feline Med Surg. 2015;17(12):1049-56.
22. Shuralev EA, Shamaev ND, Mukminov MN, Nagamune K, Taniguchi Y, Saito T, Kitoh K, Arleevskaya MI, Fedotova AY, Abdulmanova DR, Aleksandrova NM, Efimova MA, Yarullin AI, Valeeva AR, Khaertynov KS, Takashima Y*. Toxoplasma gondii* seroprevalence in goats, cats and humans in Russia. Parasitol Int. 2018;67(2):112-114
23. Montoya A, García M, Gálvez R, Checa R, Marino V, Sarquis J, Barrera JP, Rupérez C, Caballero L, Chicharro C, Cruz I, Miró G. Implications of zoonotic and vector-borne parasites to free-roaming cats in central Spain. Vet Parasitol. 2018;15;251:125-130
24. Simon JA, Pradel R, Aubert D, Geers R, Villena I, Poulle ML. A multi-event capture-recapture analysis of *Toxoplasma gondii* seroconversion dynamics in farm cats. Parasit Vectors. 2018;8;11(1):339
25. Sroka J, Karamon J, Dutkiewicz J, Wójcik Fatla A, Zając V, Cencek T. Prevalence of *Toxoplasma gondii* infection in cats in southwestern Poland. Ann Agric Environ Med. 2018 25;25(3):576-580
26. Must K, Lassen B, Jokelainen P. Seroprevalence of and Risk Factors for *Toxoplasma gondii* Infection in Cats in Estonia. Vector Borne Zoonotic Dis. 2015;15(10):597-601
27. Esteves F, Aguiar D, Rosado J, Costa ML, de Sousa B, Antunes F, Matos O*. Toxoplasma gondii* prevalence in cats from Lisbon and in pigs from centre and south of Portugal. Vet Parasitol. 2014;24;200(1-2):8-12
28. Veronesi F, Santoro A, Milardi GL, Diaferia M, Morganti G, Ranucci D, Gabrielli S. Detection of *Toxoplasma gondii* in faeces of privately owned cats using two PCR assays targeting the B1 gene and the 529-bp repetitive element. Parasitol Res. 2017;116(3):1063-1069
29. Must K, Hytönen MK, Orro T, Lohi H, Jokelainen P*. Toxoplasma gondii* seroprevalence varies by cat breed. PLoS One. 2017;8;12(9):e0184659
30. Deksne G, Petrusēviča A, Kirjušina M. Seroprevalence and Factors Associated with *Toxoplasma gondii* Infection in Domestic Cats from Urban Areas in Latvia. J Parasitol. 2013;99(1):48-50
31. De Craeye S, Francart A, Chabauty J, Van Gucht S, Leroux I, Jongert E. Toxoplasmosis in Belgian pet cats: recommendations for owners. Vlaams Diergeneeskundig Tijdschrift 2008;77(5):325-330
32. Jokelainen P, Simola O, Rantanen E, Näreaho A, Lohi H, Sukura A. Feline toxoplasmosis in Finland: cross-sectional epidemiological study and case series study. J Vet Diagn Invest. 2012;24(6):1115-24.
33. Waap H, Cardoso R, Leitao A, Nunes T, Vilares A, Gargaté MA, Meireles J, Cortes H, Angelo H. In vitro isolation and seroprevalence of *Toxoplasma gondii* in stray cats and pigeons in Lisbon, Portugal. Veterinary Parasitology 2012;187:542– 547
34. Michalski M, Platt-Samoraj A, Mikulska-Skupień E*. Toxoplasma gondii* antibodies in domestic cats in Olsztyn urban area, Poland. Wiadomooeci Parazytologiczne 2010, 56(3), 277–279
35. Spada E, Proverbio D, della Pepa A, Perego R, Baggiani L, DeGiorgi GB, Domenichini G, Ferro E, Cremonesi F. Seroprevalence of feline immunodeficiency virus, feline leukaemia virus and *Toxoplasma gondii* in stray cat colonies in northern Italy and correlation with clinical and laboratory data. J Feline Med Surg. 2012;14(6):369-77
36. Bennett AD, Gunn-Moore DA, Brewer M, Lappin MR. Prevalence of Bartonella species, haemoplasmas and *Toxoplasma* *gondii* in cats in Scotland. J Feline Med Surg. 2011;13(8):553-7
37. Györke A, Opsteegh M, Mircean V, Iovu A, Cozma V. *Toxoplasma gondii* in Romanian household cats: Evaluation of serological tests, epidemiology and risk factors. Prev Vet Med. 2011;15;102(4):321-8
38. Cong W, Elsheikha HM, Zhou N, Peng P, Qin SY, Meng QF, Qian AD. Prevalence of antibodies against *Toxoplasma gondii* in pets and their owners in Shandong province, Eastern China. BMC Infect Dis. 2018;29;18(1):430
39. Millán J, Candela MG, Palomares F, Cubero MJ, Rodríguez A, Barral M, de la Fuente J, Almería S, León-Vizcaíno L. Disease threats to the endangered Iberian lynx (*Lynx pardinus*). Vet J. 2009;182(1):114-24
40. Mancianti F, Nardoni S, Ariti G, Parlanti D, Giuliani G, Papini RA. Cross-sectional survey of *Toxoplasma gondii* infection in colony cats from urban Florence (Italy). J Feline Med Surg. 2010;12(4):351-4
41. Juvet F, Lappin MR, Brennan S, Mooney CT. Prevalence of selected infectious agents in cats in Ireland. J Feline Med Surg. 2010;12(6):476-82
42. Matsuu A, Yokota SI, Ito K, Masatani T. Seroprevalence of *Toxoplasma gondii* in free-ranging and feral cats on Amami Oshima Island, Japan. J Vet Med Sci. 2017;79(11):1853-1856
43. Salman D, Pumidonming W, Oohashi E, Igarashi M. Prevalence of *Toxoplasma gondii* and other intestinal parasites in cats in Tokachi sub-prefecture, Japan. J Vet Med Sci. 2018;29;80(6):960-967
44. Kengradomkij C, Kamyingkird K, Pinyopanuwat N, Chimnoi W, Jittapalapong S, Inpankaew T. Seroprevalence of *Toxoplasma gondii* from stray cats residing in temples, Bangkok, Thailand. J Trop Med Parasitol. 2018;41:8-14.
45. Wang S, Zhou Y, Niu J, Xie Q, Xiao T, Chen Y, Li H, Ma C, Zhang H, Liu S, Zhang Z. Seroprevalence of *Toxoplasma gondii* infection in domestic cats in central China. Parasite 2017;24:10
46. Kim SE, Choi R, Kang SW, Hyun C. Prevalence of *Toxoplasma gondii* infection in household and feral cats in Korea. J Parasit Dis. 2017;41(3):823-825
47. Boughattas S, Behnke J, Sharma A, Abu-Madi M. Seroprevalence of *Toxoplasma gondii* infection in feral cats in Qatar. BMC Veterinary Research 2017;13:26
48. Hou ZF, Su SJ, Liu DD, Wang LL, Jia CL, Zhao ZX, Ma YF, Li QQ, Xu JJ, Tao JP. Prevalence, risk factors and genetic characterization of *Toxoplasma* *gondii* in sick pigs and stray cats in Jiangsu Province, eastern China. Infect Genet Evol. 2018;60:17-25
49. Switzer AD, McMillan-Cole AC, Kasten RW, Stuckey MJ, Kass PH, Chomel BB*. Bartonella* and *Toxoplasma* infections in stray cats from Iraq. Am J Trop Med Hyg. 2013;89(6):1219-24
50. Hong SH, Jeong YI, Kim JY, Cho SH, Lee WJ, Lee SE. Prevalence of *Toxoplasma gondii* Infection in Household Cats in Korea and Risk Factors. Korean J Parasitol. 2013; 51(3): 357–361
51. Li YN, Nie X, Peng QY, Mu XQ, Zhang M, Tian MY, Min SJ. Seroprevalence and genotype of *Toxoplasma gondii* in pigs, dogs and cats from Guizhou province, Southwest China. Parasit Vectors. 2015;8:214
52. Yang Y, Ying Y, Verma SK, Cassinelli AB, Kwok OC, Liang H, Pradhan AK, Zhu XQ, Su C, Dubey JP. Isolation and genetic characterization of viable *Toxoplasma gondii* from tissues and feces of cats from the central region of China. Vet Parasitol. 2015;211(3-4):283-8
53. Kang YH, Cong W, Qin SY, Shan XF, Gao YH, Wang CF, Qian AD. First Report of *Toxoplasma gondii*, Dirofilaria immitis, and Chlamydia felis Infection in Stray and Companion Cats in Northeastern and Eastern China. Vector Borne Zoonotic Dis. 2016;16(10):654-8
54. Cong W, Meng QF, Blaga R, Villena I, Zhu XQ, Qian AD*. Toxoplasma gondii*, *Dirofilaria immitis*, feline immunodeficiency virus (FIV), and feline leukemia virus (FeLV) infections in stray and pet cats (Felis catus) in northwest China: co-infections and risk factors. Parasitol Res. 2016;115(1):217-23
55. Wang Q, Jiang W, Chen YJ, Liu CY, Shi JL, Li XT. Prevalence of *Toxoplasma gondii* antibodies, circulating antigens and DNA in stray cats in Shanghai, China. Parasit Vectors 2012;5:190
56. Can H, Döşkaya M, Ajzenberg D, Özdemir HG, Caner A, İz SG, Döşkaya AD, Atalay E, Çetinkaya Ç, Ürgen S, Karaçalı S, Ün C, Dardé ML, Gürüz Y. Genetic characterization of *Toxoplasma gondii* isolates and toxoplasmosis seroprevalence in stray cats of İzmir, Turkey. PLoS One. 2014;15;9(8):e104930
57. Ahmad N, Ahmed H, Irum S, Qayyum M. Seroprevalence of IgG and IgM antibodies and associated risk factors for toxoplasmosis in cats and dogs from subtropical arid parts of Pakistan. Tropical Biomedicine 2014;31(4):777-784
58. Tehrani-Sharif M, Jahan S, Alavi SM, Khodami M. Seroprevalence of *Toxoplasma gondii* antibodies of stray cats in Garmsar, Iran. J Parasit Dis. 2015;39(2):306-8
59. Wu SM, Zhu XQ, Zhou ,DH Fu BQ, Chen J, Yang JF, Song HQ, Weng YB, Ye DH. Seroprevalence of *Toxoplasma gondii* infection in household and stray cats in Lanzhou, northwest China. Parasites & Vectors 2011, 4:214
60. Sukhumavasi W, Bellosa ML, Lucio-Forster A, Liotta JL, Lee ACY, Pornmingmas P, Chungpivat S, Mohammed HO, Lorentzen L, Dubey JP, Bowman DD. Serological survey of *Toxoplasma gondii*, *Dirofilaria immitis*, Feline Immunodeficiency Virus (FIV) and Feline Leukemia Virus (FeLV) infections in pet cats in Bangkok and vicinities, Thailand. Veterinary Parasitology 2012;188:25-30
61. Kulasena VA, Rajapakse RPVJ, Dubey JP, Dayawansa PN, Premawansa S. Seroprevalence of *Toxoplasma gondii* in Cats from Colombo, Sri Lanka. Journal of Parasitology, 2011;97(1):152-152
62. Mosallanejad B, Avizeh, R, Razi Jalali MH, Pourmehdi M. A study on seroprevalence and coproantigen detection of *Toxoplasma gondii* in companion cats in Ahvaz area, southwestern Iran. Iranian Journal of Veterinary Research, Shiraz University 2011;12(2):139-144
63. Lee SE, Kim NH, Chae HS, Cho HS, Nam HW, Lee WJ, Kim SH, Lee JH. Prevalence of *Toxoplasma gondii* Infection in Feral Cats in Seoul, Korea. Journal of Parasitology, 2011;97(1):153-155
64. Hamidinejat H, Mosalanejad B, Avizeh R, Razi Jalali MH, Ghorbanpour M, Namavari M. *Neospora caninum* and *Toxoplasma gondii* antibody prevalence in Ahvaz feral cats, Iran. Jundishapur J Microbiol. 2011; 4(4): 217-222.
65. Akhtardanesh B, Ziaali N, Sharifi H, Rezaei S. Feline immunodeficiency virus, feline leukemia virus and *Toxoplasma gondii* in stray and household cats in Kerman–Iran: Seroprevalence and correlation with clinical and laboratory findings. Research in Veterinary Science 2010;89:306–310
66. Lee SE, Kim JY, Kim YA, Cho SH, Ahn HJ, Woo HM, Lee WJ, Nam HW. Prevalence of *Toxoplasma gondii* Infection in Stray and Household Cats in Regions of Seoul, Korea. Korean J Parasitol. 2010;48(3):267-270
67. Jittapalapong S, Inpankaew T, Pinyopanuwat N, Chimnoi W, Kengradomkij C, Wongnarkpet S, Maruyama S, Lekkla A, Sukthana Y. Epidemiology of *Toxoplasma gondii* infection of stray cats in Bangkok, Thailand. Southeast Asian J Trop Med Public Health 2010; 41(1):13-18
68. Hata H, Aosai F, Norose K, Kobayashi M, Mun HS, Chen M, Ito I, Isegawa N, Ishikawa M, Mori S, Yano A. Prevalence of *Toxoplasma gondii* and other intestinal parasites in cats in Chiba Preference Japan. Jpn. J. Trop. Med. Hyg., 2000; 28(4):365-368
69. Dubey JP, Darrington C, Tiao N, Ferreira LR, Choudhary S, Molla B, Saville WJA, Tilahun G, Kwok OCH, WA. Gebreyes Isolation of viable *Toxoplasma gondii* from tissues and feces of cats from Addis Ababa, Ethiopia. J Parasitol. 2013;99(1):56-58
70. Lobetti R, Lappin MR. Prevalence of *Toxoplasma gondii, Bartonella* species and haemoplasma infection in cats in South Africa. Journal of Feline Medicine and Surgery 2012 14: 857
71. Al-Kappany YM, Rajendran C, Ferreira LR, Kwok OCH, Abu-Elwafa SA, Hilali M, Dubey JP. High Prevalence of Toxoplasmosis in Cats from Egypt: Isolation of Viable *Toxoplasma gondii*, Tissue Distribution, and Isolate Designation. Journal of Parasitology, 2010;96(6):1115-1118
72. Kamani J, Mani AU, Hussaini A. Kumshe HA, Yidawi JP, Egwu GO. Prevalence of *Toxoplasma gondii* antibodies in cats in Maiduguri, Northeastern Nigeria. Acta Parasitologica, 2010, 55(1), 94-95
73. Aboul-Magd, L.A., Tawfik, M.S., Arafa, M.S., El-Ridi, A.M.S., 1988. *Toxoplasma* infection of cats in Cairo area as revealed by IFAT. Journal of the Egyptian Society of Parasitology 18, 403-409.
74. Abu-Zakham, A.A., El-Shazly, A.M., Yossef, M.E., Romela, S.A., Handoussa, A.E., 1989. The prevalence of *Toxoplasma gondii* antibodies among cats from Mahalla El- Kobra, Gharbia Governorate. Journal of the Egyptian Society of Parasitology 19, 225-229.
75. Lickey, A.L.A., Kennedy, M., Patton, S., Ramsay, E.C., 2005. Serologic survey of domestic felids in the Peten region of Guatemala. Journal of Zoo and Wildlife Medicine 36, 121-123
76. Sriwaranard, P., Jansawan, W., Satayapunt, C., 1981. Serologic diagnosis of toxoplasmosis in domestic cats. Kasetsart Veterinarians 2, 20-26.
77. Fujinami, F., Tanaka, H., Ohshima, S., 1983. Prevalence of protozoan and helminth parasites in cats for experimental use obtained from Kanto Area, Japan. Experimental Animals 32, 133-137.
78. Oikawa, H., Omata, Y., Kanda, M., Mikazuki, K., Yano, K., Nakabayashi, T., 1990. Survey on *Toxoplasma* infection in stray cats in western area of Japan during a two-year period. Japanese Journal of Parasitology 39, 462-467.
79. Lin, D.S., Lai, S.S., Bowman, D.D., Jacobson, R.H., Barr, M.C., Giovengo, S.L., 1990. Feline immunodeficiency virus, feline leukemia virus, *Toxoplasma gondii*, and intestinal parasitic infections in Taiwanese cats. British Veterinary Journal 146, 468-475.
80. Shen, L., Zhichung, L., Biaucheng, Z., Huayuan, Y., 1990. Prevalence of *Toxoplasma gondii* infection in man and animals in Guangdong, People’s Republic of China. Veterinary Parasitology 34, 357-360.
81. Furuya, H., Nogami, S., Inoue, I., Takahashi, D., Mizusawa, H., Tohyama, K., 1993. Seroepidemiological survey on *Toxoplasma gondii* infection in kittens. Japanese Journal of Parasitology 42, 388-391
82. Chong, L.H., Singh, M., Chua, S.B., Fong, W.E., 1993. Feline toxoplasmosis in Singapore. Singapore Veterinary Journal 17, 79-87
83. Fu, B., Zhao, Y.Q., Yang, B.J., Han, G.D., Shao, Q.F., Li, J.M., Zhang, Y.L., Wang, Z.M., Sun, D.J., Yu, G.Z., Wang, X.J., 1995. Epidemiological investigation on toxoplasmosis in Shandong province. Chinese Journal of Parasitic Disease Control 8, 205-207.
84. Inci, A., Budak, C., Dincer, S., 1996. Investigation of anti-*Toxoplasma gondii* in cats using Sabin–Feldman dye test in Ankara. Turkiye Parazitoloji Dergisi 20, 407-411.
85. Samad, A., Islam, R., Dey, B.C., Alam, M., 1997. Effects of corticosteroids in stray cats with natural antibodies to *Toxoplasma gondii*. Journal of Protozoological Research 7, 1–8.
86. Lu, Q., Huang, A.M., Yang, Y.M., Yang, H.Z., Yang, J.H., 1997. Serological investigation of *Toxoplasma gondii* infection in pet cats in Shanghai. Chinese Journal of Zoonoses 13, 69–78.
87. Tsai, Y.J., Fan, C.K., Chung, W.C., Chao, P.H., 1997. Seroepidemiology of *Toxoplasma gondii* infection among cats in the middle and northern Taiwan. Chinese Journal of Parasitology and Parasitic Diseases 10, 11-16.
88. Maruyama, S., Hiraga, S., Yokoyama, E., Naoi, M., Tsuruoka, Y., Ogura, Y., Tamura, K., Namba, S., Kameyama, Y., Nakamura, S., Katsube, Y., 1998. Seroprevalence of Bartonella henselae and Toxoplasma gondii infections among pet cats in Kanagawa and Saitama Prefectures. Journal of Veterinary Medical Science 60, 997-1000.
89. Nogami, S., Moritomo, T., Kamata, H., Tamura, Y., Sakai, T., Nakagaki, K., Motoyoshi, S., 1998. Seroprevalence against *Toxoplasma gondii* in domiciled cats in Japan. Journal of Veterinary Medical Science 60, 1001-1004
90. Sohn, W.M., Nam, H.W., 1999. Western blot analysis of stray cat sera against *Toxoplasma gondii* and the diagnostic availability of monoclonal antibodies in sandwich-ELISA. Korean Journal of Parasitology 37, 249-256
91. Chen, C.Y., 2001. The study of individuals having pet tend to *Toxoplasma* infection. Chinese Journal of Zoonoses 17, 76-77.
92. Kimbita, E.N., Xuan, X., Huang, X., Miyazawa, T., Fukumoto, S., Mishima, M., Suzuki, H., Sugimoto, C., Nagasawa, H., Fujisaki, K., Suzuki, N., Mikami, T., Igarashi, I., 2001. Serodiagnosis of *Toxoplasma gondii* infection in cats by enzyme-linked immunosorbent assay using recombinant SAG1. Veterinary Parasitology 102, 35-44.
93. Huang, X., Kimbita, E.N., Battur, B., Miyazawa, T., Fukumoto, S., Mishima, M., Makala, L.H., Suzuki, H., Sugimoto, C., Nagasawa, H., Fujisaki, K., Mikami, T., Igarashi, I., 2002. Development and evaluation of an enzyme-linked immunosorbent assay with recombinant SAG2 for diagnosis of *Toxoplasma gondii* infection in cats. Journal of Parasitology 88, 804-807.
94. Maruyama, S., Kabeya, H., Nakao, R., Tanaka, S., Sakai, T., Xuan, X., Katsube, Y., Mikami, T., 2003. Seroprevalence of Bartonella henselae, *Toxoplasma gondii*, FIV and FeLV infections in domestic cats in Japan. Microbiology and Immunology 47, 147-153.
95. Sukthana, Y., Kaewkungwal, J., Jantanavivat, C., Lekkla, A., Chiabchalard, R., Aumarm, W., 2003. *Toxoplasma gondii* antibody in Thai cats and their owners. Southeast Asian Journal of Tropical Medicine and Public Health 34, 733-738
96. Yuan, W.Y., Ma, K., Yang, H.L., 2004. Survey of *Toxoplasma* infection in animals in Hebei Province. Chinese Journal of Schistosomiasis Control 16, 72-76
97. Salant, H., Spira, D.T., 2004. A cross-sectional survey of anti-*Toxoplasma gondii* antibodies in Jerusalem cats. Veterinary Parasitology 124, 167–177
98. Huang, X., Xuan, X., Hirata, H., Yokoyama, N., Xu, L., Suzuki, N., Igarashi, I., 2004. Rapid immunochromatographic test using recombinant SAG2 for detection of antibodies against *Toxoplasma gondii* in cats. Journal of Clinical Microbiology 42, 351–353.
99. Brown, AS, Lappin MR, Brown JL, Munkhtsog B, Swanson WF. Exploring the ecologic basis for extreme susceptibility of Pallas cats (*Otocolobus manul*) to fatal toxoplasmosis. Journal of Wildlife Diseases, 41(4):691-700
100. Chen, D.X., Tan, J.Q., Hu, Y., Shen, H.X., Li, X.M., Huo, J., Liu, J.N., Zhang, Y.C., Cai, Y.Q., 2005. Epidemiological investigation of *Toxoplasma* *gondii* infection in cats and dogs in Guangzhou. Journal of Tropical Medicine 5, 639–641.
101. Haddadzadeh, H.R., Khazraiinia, P., Aslani, M., Rezaeian, M., Jamshidi, S., Taheri, M., Bahonar, A., 2006. Seroprevalence of *Toxoplasma gondii* infection in stray and household cats in Tehran. Veterinary Parasitology 138, 211-216
102. Yu, Y.L., Fu, L.J., Wang, M., 2006. Serological survey of *Toxoplasma gondii* infection in dogs and cats in Beijing. Chinese Journal of Veterinary Medicine 42, 72-76.
103. Shahzad, A., Khan, M.S., Ashraf, K., Avais, M., Pervez, K., Khan, J.A., 2006. Seroepidemiological and haematological studies on toxoplasmosis in cats, dogs and their owners in Lahore, Pakistan. Journal of Protozoological Research 16, 60–73.
104. Jittapalapong, S., Nimsupan, B., Pinyopanuwat, N., Chimnoi, W., Kabeya, H., Maruyama, S., 2007. Seroprevalence of *Toxoplasma gondii* antibodies in stray cats and dogs in the Bangkok metropolitan area, Thailand. Veterinary Parasitology 145, 138–141.
105. Hooshyar, H., Rostamkhani, P., Talari, S., Arbabi, M., 2007. *Toxoplasma gondii* infection in stray cats. Iranian Journal of Parasitology 2, 18–22.
106. Dubey, J.P., Zhu, X.Q., Sundar, N., Zhang, H., Kwok, O.C.H., Su, C., 2007. Genetic and biologic characterization of *Toxoplasma gondii* isolates of cats from China. Veterinary Parasitology 145, 352–356
107. Yu, J., Ding, J., Xia, Z., Lin, D., Li, Y., Jia, J., Liu, Q., 2008. Seroepidemiology of *Toxoplasma gondii* in pet dogs and cats in Beijing, China. Acta Parasitologica 53, 317–319.
108. Karatepe, B., Babur, C., Karatepe, M., Kilic, S., Dundar, B., 2008. Prevalence of *Toxoplasma gondii* and intestinal parasites in stray cats from Nigde, Turkey. Italian Journal of Animal Science 7, 113–118.
109. Lee, J.Y., Lee, S.E., Lee, E.G., Song, K.H., 2008. Nested PCR-based detection of *Toxoplasma gondii* in German shepherd dogs and stray cats in South Korea. Research in Veterinary Science 85, 125–127.
110. Chandrawathani, P., Nurulaini, R., Zaini, C.M., Premmaalatha, B., Adnan, M., Jamnah, O., Khor, S.K., Khadijah, S., Lai, S.Z., Shaik, M.A.B., Seah, T.C., Zatil, S.A., 2008. Seroprevalence of *Toxoplasma gondii* antibodies in pigs, goats, cattle, dogs and cats in peninsular Malaysia. Tropical Biomedicine 25, 257–258.
111. Kim, H.Y., Kim, Y.A., Kang, S., Lee, H.S., Rhie, H.G., Ahn, H.J., Nam, H.W., Lee, S.E., 2008. Prevalence of *Toxoplasma gondii* in stray cats of Gyeonggi-do, Korea. Korean Journal of Parasitology 46, 199–201.
112. Ozkan, A.T., Celebi, B., Babur, C., Lucio-Forster, A., Bowman, D.D., Lindsay, D.S., 2008. Investigation of anti-*Toxoplasma gondii* antibodies in cats of the Ankara region of Turkey using the Sabin–Feldman dye test and an indirect fluorescent antibody test. Journal of Parasitology 94, 817–820.
113. Sharif, M., Daryani, A., Nasrolahei, M., Ziapour, S.P., 2009. Prevalence of *Toxoplasma gondii* antibodies in stray cats in Sari, northern Iran. Tropical Animal Health and Production 41, 183–187.
114. Milstein, T.C., Goldsmid, J.M., 1997. Parasites of feral cats from southern Tasmania and their potential significance. Australian Veterinary Journal 75, 218–219.
115. Sumner, B., Ackland, M.L., 1999. *Toxoplasma gondii* antibody in domestic cats in Melbourne. Australian Veterinary Journal 77, 447-449.
116. Svoboda, M., Svobodova, V., Konrad, J., 1988. Vztahy mezi vyskytem protilatek proti *Toxoplasma gondii* a klinickymi nalezy vysetrenych kocek. Veterinarni Medicina 33, 119–126.
117. Knaus, B.U., Fehler, K., 1989. *Toxoplasma gondii*-Infektionen und Oozystenausscheidung bei Hauskatzen und ihre Bedeutung fur die Epidemiologie und Epizootiologie der Toxoplasmose. Angewandte Parastiologie 30, 155–160.
118. Uggla, A., Mattson, S., Juntti, N., 1990. Prevalence of antibodies to *Toxoplasma gondii* in cats, dogs and horses in Sweden. Acta Veterinaria Scandinavica 31, 219–222
119. Ljungstrom, B.L., Lunden, A., Hoglund, J., Zakrisson, G., 1994. Evaluation of a direct agglutination test for detection of antibodies against *Toxoplasma gondii* in cat, pig and sheep sera. Acta Veterinaria Scandinavica 35, 213–216.
120. Tenter, A.M., Vietmeyer, C., Johnson, A.M., Janitschke, K., Rommel, M., Lehmacher, W., 1994. ELISAs based on recombinant antigens for Seroepidemiological studies on *Toxoplasma gondii* infections in cats. Parasitology 109, 29–36.
121. Edelhofer, R., Aspock, H., 1996. Infektionsquellen und Infektionswege aus der Sicht des *Toxoplasmose*-Screenings der Schwangeren in Osterreich. Mitteilungen Osterreichischen Gesellschaft fur Tropenmedizin und Parasitolologie 18, 59–70.
122. D’Amore, E., Falcone, E., Busani, L., Tollis, M., 1997. A serological survey of feline immunodeficiency virus and *Toxoplasma gondii* in stray cats. Veterinary Research Communications 21, 355–359.
123. Svobodova, V., Knotek, Z., Svoboda, M., 1998. Prevalence of IgG and IgM antibodies specific to *Toxoplasma gondii* in cats. Veterinary Parasitology 80, 173–176.
124. Hecking-Veltman, J., Tenter, A.M., Daugschies, A., 2001. Studien zur parasitenfauna bei streunenden katzen im raum monchengladbach. Der Praktische Tierarzt 82, 563–569.
125. Dorny, P., Speybroeck, N., Verstraete, S., Baeke, M., de Becker, A., Berkvens, D., Vercruysse, J., 2002. Serological survey of *Toxoplasma gondii*, feline immunodeficiency virus and feline leukaemia virus in urban stray cats in Belgium. Veterinary Record 151, 626–629.
126. Gauss, C.B.L., Almeria, S., Ortuno, A., Garcia, F., Dubey, J.P., 2003. Seroprevalence of *Toxoplasma gondii* antibodies in domestic cats from Barcelona, Spain. Journal of Parasitology 89, 1067–1068
127. Michalski, M., Platt-Samoraj, A., 2004. Prevalence of *Toxoplasma gondii* infection in cats from Olsztyn city. Wiadomosci Parazytologicze 50, 303–305
128. Miro, G., Montoya, A., Jimenez, S., Frisuelos, C., Mateo, M., Fuentes, I., 2004. Prevalence of antibodies to *Toxoplasma gondii* and intestinal parasites in stray, farm and household cats in Spain. Veterinary Parasitology 126, 249–255
129. Afonso, E., Thulliez, P., Gilot-Fromont, E., 2006. Transmission of Toxoplasma gondii in an urban population of domestic cats (Felis catus). International Journal for Parasitology 36, 1373–1382.
130. Sedlak, K., Bartova, E., 2006. The prevalence of *Toxoplasma gondii* IgM and IgG antibodies in dogs and cats from the Czech Republic. Veterinari Medicina 51, 555–558.
131. Ondrejka, R., Ondrejkova, A., Suli, J., Benisek, Z., Posivakova, S., Svrcek, S., 2007. AIDes, leukaemias es toxoplasmosisos fertozesek javanytani es szerologiai felmerese a hazimacskak szlovakiai populaciojaban. Magyar Allatorvosok Lapja 129, 628–631.
132. Natale, A., Frangipane di Regalbono, A., Zanellato, G., Cavalletto, M., Danesi, P., Capelli, G., Pietrobelli, M., 2007. Parasitological survey on stray cat colonies from the Veneto region. Veterinary Research Communications 31 (Suppl. 1), 241–244.
133. Lopes, A.P., Cardoso, L., Rodrigues, M., 2008. Serological survey of *Toxoplasma gondii* infection in domestic cats from northeastern Portugal. Veterinary Parasitology 155, 184–189.
134. De Craeye, S., Francart, A., Chabauty, J., De Vriendt, V., Van Gucht, S., Leroux, I., Jongert, E., 2008. Prevalence of *Toxoplasma gondii* infection in Belgian house cats. Veterinary Parasitology 157, 128–132.
135. Hornok, S., Edelhofer, R., Joachim, A., Farkas, R., Berta, K., Repasi, A., Lakatos, B., 2008. Seroprevalence of *Toxoplasma gondii* and *Neospora caninum* infection of cats in Hungary. Acta Veterinaria Hungarica 56, 81–88.
136. Montoya, A., Miro, G., Mateo, M., Ramirez, C., Fuentes, I., 2008. Molecular characterization of *Toxoplasma gondii* isolates from cats from Spain. Journal of Parasitology 94, 1044–1046.
137. Marchiondo, A.A., Duszynski, D.W., Maupin, G.O., 1976. Prevalence of antibodies to *Toxoplasma gondii* in wild and domestic animals of New Mexico, Arizona and Colorado. Journal of Wildlife Diseases 12, 226–232.
138. Witt, C.J., Moench, T.R., Gittelsohn, A.M., Bishop, B.D., Childs, J.E., 1989. Epidemiologic observations on feline immunodeficiency virus and *Toxoplasma gondii* coinfection in cats in Baltimore, MD. Journal of the American Veterinary Medical Association 194, 229–233.
139. Lappin, M.R., Greene, C.E., Prestwood, A.K., Dawe, D.L., Marks, A., 1989. Prevalence of *Toxoplasma gondii* infection in cats in Georgia using enzyme-linked immunosorbent assays for IgM, IgG, and antigens. Veterinary Parasitology 33, 225–230.
140. Rodgers, S.J., Baldwin, C.A., 1990. A serologic survey of Oklahoma cats for antibodies to feline immunodeficiency virus, coronavirus, and *Toxoplasma gondii* and for antigen to feline leukemia virus. Journal of Veterinary Diagnostic Investigation 2, 180–183
141. Lappin, M.R., Marks, A., Greene, C.E., Collins, J.K., Carman, J., Reif, J.S., Powell, C.C., 1992. Serologic prevalence of selected infectious diseases in cats with uveitis. Journal of the American Veterinary Medical Association 201, 1005–1009.
142. Smith, K.E., Zimmerman, J.J., Patton, S., Beran, G.W., Hill, J.T., 1992. The epidemiology of toxoplasmosis on Iowa swine farms with an emphasis on the roles of free-living mammals. Veterinary Parasitology 42, 199–211.
143. Dubey, J.P., Weigel, R.M., Siegel, A.M., Thulliez, P., Kitron, U.D., Mitchell, M.A., Mannelli, A., Mateus-Pinilla, N.E., Shen, S.K., Kwok, O.C.H., Todd, K.S., 1995. Sources and reservoirs of *Toxoplasma gondii* infection on 47 swine farms in Illinois. Journal of Parasitology 81, 723–729.
144. Quesnel, A.D., Parent, J.M., McDonell, W., Percy, D., Lumsden, J.H., 1997. Diagnostic evaluation of cats with seizure disorders: 30 cases (1991–1993). Journal of the American Veterinary Medical Association 210, 65–71.
145. Hill, R.E., Zimmerman, J.J., Willis, R.W., Patton, S., Clark, W.R., 1998. Seroprevalence of antibodies against *Toxoplasma gondii* in free-ranging mammals in Iowa. Journal of Wildlife Diseases 34, 811–815.
146. Aramini, J.J., Stephen, C., Dubey, J.P., Engelstoft, C., Schwantje, H., Ribble, C.S., 1999. Potential contamination of drinking water with *Toxoplasma* *gondii* oocysts. Epidemiology and Infection 122, 305–315.
147. Hill, S.L., Cheney, J.M., Taton-Allen, G.F., Reif, J.S., Bruns, C., Lappin, M.R., 2000. Prevalence of enteric zoonotic organisms in cats. Journal of the American Veterinary Medical Association 216, 687–692.
148. DeFeo, M.L., Dubey, J.P., Mather, T.N., Rhodes, R.C., 2002. Epidemiologic investigation of seroprevalence of antibodies to *Toxoplasma gondii* in cats and rodents. American Journal of Veterinary Research 63, 1714–1717.
149. Dubey, J.P., Saville, W.J.A., Stanek, J.F., Reed, S.M., 2002. Prevalence of *Toxoplasma gondii* antibodies in domestic cats from rural Ohio. Journal of Parasitology 88, 802–803.
150. Luria, B.J., Levy, J.K., Lappin, M.R., Breitschwerdt, E.B., Legendre, A.M., Hernandez, J.A., Gorman, S.P., Lee, I.T., 2004. Prevalence of infectious diseases in feral cats in Northern Florida. Journal of Feline Medicine Surgery 6, 287–296.
151. Nutter, F.B., Dubey, J.P., Levine, J.F., Breitschwerdt, E.B., Ford, R.B., Stoskopf, M.K., 2004. Seroprevalence of antibodies against *Bartonella henselae* and *Toxoplasma gondii* and fecal shedding of *Cryptosporidium* spp., and *Toxocara cati* in feral and pet domestic cats. Journal of the American Veterinary Medical Association 225, 1394–1398.
152. Vollaire, M.R., Radecki, S.V., Lappin, M.R., 2005. Seroprevalence of *Toxoplasma gondii* antibodies in clinically ill cats in the United States. American Journal of Veterinary Research 66, 874–877.
153. Danner, R.M., Goltz, D.M., Hess, S.C., Banko, P.C., 2007. Evidence of feline immunodeficiency virus, feline leukemia virus, and *Toxoplasma gondii* in feral cats on Mauna Kea, Hawaii. Journal of Wildlife Diseases 43, 315–318.
154. Dabritz, H.A., Gardner, I.A., Miller, M.A., Lappin, M.R., Atwill, E.R., Packham, A.E., Melli, A.C., Conrad, P.A., 2007a. Evaluation of two *Toxoplasma gondii* serologic tests used in a serosurvey of domestic cats in California. Journal of Parasitology 93, 806–816.
155. Miller, M.A., Miller, W.A., Conrad, P.A., James, E.R., Melli, A.C., Leutenegger, C.M., Dabritz, H.A., Packham, A.E., Paradies, D., Harris, M., Ames, J., Jessup, D.A., Worcester, K., Grigg, M.E., 2008. Type X Toxoplasma gondii in a wild mussel and terrestrial carnivores from coastal California: new linkages between terrestrial mammals, runoff and toxoplasmosis of sea otters. International Journal for Parasitology 38, 1319–1328.
156. de Camps, S., Dubey, J.P., Saville, W.J.A., 2008. Seroepidemiology of *Toxoplasma gondii* in zoo animals in selected zoos in the midwestern United States. Journal of Parasitology 94, 648–653
157. Dubey, J.P., Bhatia, C.R., Lappin, M.R., Ferreira, L.R., Thorn, A., Kwok, O.C.H., 2009b. Seroprevalence of *Toxoplasma gondii* and *Bartonella* spp. antibodies in cats from Pennsylvania. Journal of Parasitology. 2009; 95(3):578-80.
158. Stutzin, M., Contreras, M.C., Schenone, H. [Epidemiology of *Toxoplasma gondii* in Chile. V. Prevalence of infection in humans and in wild and domestic mammals, assessed using the indirect haemagglutination reaction, in Juan Fernandez Archipelago, Region 5, Chile. [Spanish].](http://ovidsp.tx.ovid.com/sp-3.31.1b/ovidweb.cgi?&S=IIPAFPDDHLDDEIPFNCEKOBGCCILLAA00&Complete+Reference=S.sh.69|513|1)  Boletin Chileno de Parasitologia 1989;44(1/2):37-48.
159. Fernandez, F., Ouvina, G., Clot, E., Fernandes Guido, R., Codoni, C. Prevalence of *Toxoplasma gondii* antibodies in cats in the western part of Great Buenos Aires, Argentina, 1993. Veterinary Parasitology 1995;59(1):75–79.
160. Venturini, M.C., Di Lorenzo, C., Castellano, M.C., Unzaga, J.M., Venturini, L., 1995. Deteccion de anticuerpos anti-*Toxoplasma gondii* en gatos mediante las pruebas de immunofluorescencia y de aglutinacion de latex. Veterinaria Argentina 12, 48–50.
161. Frenkel, J.K., Hassanein, K.M., Hassanein, R.S., Brown, E., Thulliez, P., Quintero- Nunez, R., 1995. Transmission of *Toxoplasma gondii* in Panama City, Panama: a five-year prospective cohort study of children, cats, rodents, birds, and soil. American Journal of Tropical Medicine and Hygiene 53, 458–468.
162. Venturini, L., Venturini, M.C., Omata, Y., Di Lorenzo, C., De Carolls, G., 1997a. Toxoplasma gondii: La respuesta inmune por IgG durante el periodo patente en un gato domestico infectado naturalmente. Revista de Medicina Veterinaria 78, 258–260.
163. Londono, M.T.M., Chamorro, N.L., Infante, M.S., Carlos, J., Osorio, C., 1998. Infeccion por *Toxoplasma gondii* en gatos de dos barrios del sur de Armenia y su importancia en la toxoplasmosis humana. Colbaquin Actualidades Clinicas y Biotecnologicas 12, 18–23.
164. Lucas, S.R.R., Hagiwara, M.K., Loureiro, V.d.S., Ikesaki, J.Y.H., Birgel, E.H., 1999. *Toxoplasma gondii* infection in Brazilian domestic outpatient cats. Revista do Instituto de Medicina Tropical de Sao Paulo 41, 221–224.
165. Garcia, J.L., Navarro, I.T., Ogawa, L., de Oliveira, R.C., 1999. Soroepidemiologia da toxoplasmose em gatos e caes de propriedades rurais do municipio de Jaguapita, Estado do Parana, Brasil. Ciencia Rural 29, 99–104.
166. Galvan Ramirez, M.L., Sanchez Vargas, G., Vielma Sandoval, M., Soto Mancilla, J.L., 1999. Presence of anti-*Toxoplasma* antibodies in humans and their cats in the urban zone of Guadalajara. Revista da Sociedade Brasileira de Medicina Tropical 32, 483–488.
167. Ovalle, F., Garcia, A., Thibauth, J., Lorca, M., 2000. Frecuencia de anticuerpos anti-*Toxoplasma* *gondii* en gatos de la ciudad de Valdivia, Chile. Boletin Chileno de Parasitologia 55, 94–99.
168. da Silva, A.V., Cutolo, A.A., Langoni, H., 2002. Comparacao da reacao de imunofluorescencia indireta e do metodo de aglutinacao direta na deteccao de anticorpos anti-*Toxoplasma* em soros de ovinos, caprinos, caninos e felinos. Arquivos do Instituto Biologico (Sao Paulo) 69, 7–11.
169. Netto, E.G., Munhoz, A.D., Albuquerque, G.R., Lopes, C.W.G., Ferreira, A.M.R., 2003. Ocorrencia de gatos soropositivos para *Toxoplasma gondii* Nicolle e Manceaux, 1909 (Apicomplexa: Toxoplasmatinae) na Cidade de Niteroi, Rio de Janeiro. Revista Brasileira de Parasitologia Veterinaria 12, 145–149.
170. Dubey, J.P., Navarro, I.T., Sreekumar, C., Dahl, E., Freire, R.L., Kawabata, H.H., Vianna, M.C.B., Kwok, O.C.H., Shen, S.K., Thulliez, P., Lehmann, T., 2004b. *Toxoplasma* *gondii* infections in cats from Parana, Brazil: seroprevalence, tissue distribution, and biologic and genetic characterization of isolates. Journal of Parasitology 90, 721–726.
171. Meireles, L.R., Galisteo, A.J., Pompeu, E., Andrade, H.F., 2004. *Toxoplasma gondii* spreading in an urban area evaluated by seroprevalence in free-living cats and dogs. Tropical Medicine and International Health 9, 876–881.
172. Pena, H.F.J., Soares, R.M., Amaku, M., Dubey, J.P., Gennari, S.M., 2006. *Toxoplasma gondii* infection in cats from Sao Paulo state, Brazil: seroprevalence, oocyst shedding, isolation in mice, and biologic and molecular characterization. Research in Veterinary Science 81, 58–67.
173. Dubey, J.P., Su, C., Cortes, J.A., Sundar, N., Gomez-Marin, J.E., Polo, L.J., Zambrano, L., Mora, L.E., Lora, F., Jimenez, J., Kwok, O.C.H., Shen, S.K., Zhang, X., Nieto, A., Thulliez, P., 2006. Prevalence of *Toxoplasma gondii* in cats from Colombia, South America and genetic characterization of *T. gondii* isolates. Veterinary Parasitology 141, 42–47.
174. Cavalcante, G.T., Aguiar, D.M., Chiebao, D., Dubey, J.P., Ruiz, V.L.A., Dias, R.A., Camargo, L.M.A., Labruna, M.B., Gennari, S.M., 2006b. Seroprevalence of *Toxoplasma gondii* antibodies in cats and pigs from rural western Amazon, Brazil. Journal of Parasitology 92, 863–864.
175. Afonso, E., Thulliez, P., Pontier, D., Gilot-Fromont, E., 2007. Toxoplasmosis in prey species and consequences for prevalence in feral cats: not all prey species are equal. Parasitology 134, 1963–1971.
176. Dubey, J.P., Lopez-Torres, H.Y., Sundar, N., Velmurugan, G.V., Ajzenberg, D., Kwok, O.C.H., Hill, R., Darde, M.L., Su, C., 2007b. Mouse-virulent *Toxoplasma gondii* isolated from feral cats on Mona Island, Puerto Rico. Journal of Parasitology 93, 1365–1369.
177. Garcia-Marquez, L.J., Gutierrez-Diaz, M.A., Correa, D., Luna-Pasten, H., Palma, J.M., 2007. Prevalence of *Toxoplasma gondii* antibodies and the relation to risk factors in cats of Colima, Mexico. Journal of Parasitology 93, 1527–1528.
178. Alvarado-Esquivel, C., Liesenfeld, O., Herrera-Flores, R.G., Ramirez-Sanchez, B.E., Gonzalez-Herrera, A., Martinez-Garcia, S.A., Dubey, J.P., 2007. Seroprevalence of *Toxoplasma gondii* antibodies in cats from Durango City, Mexico. Journal of Parasitology 93, 1214–1216.
179. Moura, L., Kelly, P., Krecek, R.C., Dubey, J.P., 2007. Seroprevalence of *Toxoplasma gondii* in cats from St. Kitts, West Indies. Journal of Parasitology 93, 952–953.
180. Mendes-de-Almeida, F., Labarthe, N., Guerrero, J., Faria, M.C.F., Branco, A.S., Pereira, C.D., Barreira, J.D., Pereira, M.J.S., 2007. Follow-up of the health conditions of an urban colony of free-roaming cats (Felis catus Linnaeus, 1758) in the city of Rio de Janeiro, Brazil. Veterinary Parasitology 147, 9–15.
181. Levy, J.K., Crawford, P.C., Lappin, M.R., Dubovi, E.J., Levy, M.G., Alleman, R., Tucker, S.J., Clifford, E.L., 2008. Infectious diseases of dogs and cats on Isabela, Island, Galapagos. Journal of Veterinary Internal Medicine 22, 60–65.
182. Besne-Merida, A., Figueroa-Castillo, J.A., Martinez-Maya, J.J., Pasten, H.L., Calderon- Segura, E., Correa, D., 2008. Prevalence of antibodies against *Toxoplasma gondii* in domestic cats from Mexico City. Veterinary Parasitology 157, 310–313.
183. Dubey, J.P., Velmurugan, G.V., Alvarado-Esquivel, C., Alvarado-Esquivel, D., Rodgriguez-Pena, S., Martinez-Garcia, S., Gonzalez-Herrera, A., Ferreira, L.R., Kwok, O.C.H., Su, C., 2009a. Isolation of Toxoplasma gondii from animals in Durango, Mexico. Journal of Parasitology 95, 319–322.
184. Dubey, J.P., Lappin, M.R., Kwok, O.C.H., Mofya, S., Chikweto, A., Baffa, A., Doherty, D., Shakier, J., Macpherson, C.L., Sharma, R.N., 2009. Seroprevalence of *Toxoplasma gondii* and concurrent *Bartonella* spp., feline immunodeficiency virus, and feline leukemia infections in cats from Grenada, West Indies. Journal of Parasitology, 2009; 1:1129-33.
185. Dubey, J.P., Moura, L., Majumdar, D., Sundar, N., Velmurugan, G.V., Kwok, O.C.H., Kelly, P., Krecek, R.C., Su, C., 2009. Isolation and characterization of viable *Toxoplasma gondii* isolates revealed possible high frequency of mixed infection in feral cats (*Felis domesticus*) from St Kitts, West Indies. Parasitology 136, 589–594.
186. Dubey JP, Pas A, Rajendran C, Kwok OCH, Ferreira LR, Martins J, Hebel C, Hammer S, Su C. Toxoplasmosis in Sand cats (Felis margarita) and other animals in the Breeding Centre for Endangered Arabian Wildlife in the United Arab Emirates and Al Wabra Wildlife Preservation, the State of Qatar. Veterinary Parasitology 2010;172:195-203.
187. Bevins SN, Carver S, Boydston EE, Lyren LM, Alldredge M, Logan KA, Riley SPD, Fisher RN, Vickers TW, Boyce W, Salman M, Lappin MR, Crooks KR, VandeWoude S. Three Pathogens in Sympatric Populations of Pumas, Bobcats, and Domestic Cats: Implications for Infectious Disease Transmission. PLoS ONE 2012;7(2): e31403
188. Ladiges WC. et al. Prevalence of *Toxoplasma gondii* antibodies and oocysts in pound-source cats. [J Am Vet Med Assoc.](https://www.ncbi.nlm.nih.gov/pubmed/7096176) 1982;180(11):1334-5
189. Da Silva JCR, Gennari S, M. Ragozo AMA, Amajones VR, Magnabosco C, Yai LEO, Ferreira Neto JS, Dubey JP. [Prevalence of *Toxoplasma gondii* antibodies in sera of domestic cats from Guarulhos and Sao Paulo, Brazil.](http://ovidsp.tx.ovid.com/sp-3.31.1b/ovidweb.cgi?&S=IIPAFPDDHLDDEIPFNCEKOBGCCILLAA00&Complete+Reference=S.sh.69|396|1)  Journal of Parasitology 2002;88(2):419-420
190. Tiao N, Darrington C, Molla B, Saville WJA, Tilahun G, Kwok OCH, Gebreyes WA, Lappin MR, Jones JL, Dubey JP. An investigation into the seroprevalence of *Toxoplasma* *gondii*, *Bartonella* spp. feline immunodeficiency virus (FIV), and feline leukemia virus (FelV) cats from Addis Ababa, Ethiopia. Epidemiol. Infect. 2013;141(5):1029-33
191. Lopes AP, Oliveira AC, Granada S, Rodrigues FT, Papadopoulos E, Schallig H, Dubey JP, Cardoso L. Antibodies to *Toxoplasma gondii* and *Leishmania* spp. in domestic cats from Luanda, Angola. Vet Parasitol. 2017;239:15-18
192. Yekkour F, Aubert D, Mercier A, Murat JB, Khames M, Nguewa P, Ait-Oudhia K, Villena I, Bouchene Z. First genetic characterization of *Toxoplasma gondii* in stray cats from Algeria. Vet Parasitol. 2017;239:31-36.
193. Khin-Sane-Win, Matsumura T, Kumagai S, Uga S, Konishi E. Prevalence of antibody to *Toxoplasma gondii* in Hyogo Prefecture, Japan: comparison at a 10-year interval. Kobe J Med Sci. 1997;43(5):159-68
194. Raeghi S, Sedighi S, Sedighi S. Prevalence of *Toxoplasma gondii* antibodies in cats in Urmia, Northwest of Iran. The Journal of Animal & Plant Sciences, 2011;21(2):132-134
195. Qian W, Wang H, Su C, Shan D, Cui X, Yang N, Lv C, Liu Q. Isolation and characterization of *Toxoplasma gondii* strains from stray cats revealed a single genotype in Beijing, China. Vet Parasitol. 2012;187(3-4):408-13
196. Yang YR, Feng YJ, Lu YY, Dong H, Li TY, Jiang YB, Zhu XQ, Zhang LX. Antibody Detection, Isolation, Genotyping, and Virulence of *Toxoplasma gondii* in Captive Felids from China. Front Microbiol. 2017;8:1414
197. Abdou NE, Al-Batel MK, El-Azazy OM, Sami AM, Majeed QA. Enteric protozoan parasites in stray cats in Kuwait with special references to toxoplasmosis and risk factors affecting its occurrence. J Egypt Soc Parasitol. 2013;43(2):303-14.
198. Smielewska-Loś E, Pacoń J*. Toxoplasma gondii* infection of cats in epizootiological and clinical aspects. Pol J Vet Sci. 2002;5(4):227-30.
199. Opsteegh M, Haveman R, Swart AN, Mensink-Beerepoot ME, Hofhuis A, Langelaar MF, van der Giessen JW. Seroprevalence and risk factors for *Toxoplasma gondii* infection in domestic cats in The Netherlands. Prev Vet Med. 2012;104(3-4):317-26
200. Claus GE, Christie E, Dubey JP. Prevalence of *Toxoplasma* antibody in feline sera. J. Parasitol. 1977;63:266–266.
201. Wallace GD. The role of the cat in the natural history of *Toxoplasma gondii*. Am J Trop Med Hyg. 1973;22(3):313-22
202. Ruiz A, Frenkel JK*. Toxoplasma gondii* in Costa Rican cats. Am J Trop Med Hyg. 1980;29(6):1150-60
203. Salata E, Yoshida ELA.; Pereira EA, Correa FMA. Toxoplasmose em animais silvestres e domésticos da região de Botucatu, Estado de São Paulo, Brasil. **Rev. Inst. Med. trop. S. Paulo,** 1985;**27**: 20-22.
204. Stojanovic V, Foley P. Infectious disease prevalence in a feral cat population on Prince Edward Island, Canada. Can Vet J 2011;52:979-982
205. Tutuncu M, Akkan HA, Karaca M, Agaoglu Z, Berktas M. Prevalence of toxoplasmosis in Van cats in Turkey. The Indian veterinary Journal 80(8):730-732
206. Jackson MH, Hutchison WM, Siim JC. Prevalence of *Toxoplasma gondii* in meat animals, cats and dogs in central Scotland. Br Vet J. 1987;143(2):159-65.
207. McColm AA, Hutchison WM, Siim JC. The prevalence of *Toxoplasma gondii* in meat animals and cats in central Scotland. Annals of Tropical Medicine & Parasitology. 1981; 75(2):157-164
208. Werner JK, Walton BC. [Prevalence of naturally occurring *Toxoplasma gondii* infections in cats from U.S. military installations in Japan.](http://ovidsp.tx.ovid.com/sp-3.31.1b/ovidweb.cgi?&S=IIPAFPDDHLDDEIPFNCEKOBGCCILLAA00&Complete+Reference=S.sh.69|35|1)  J Parasitol. 1972;58(6):1148-1150.
209. Beeck LV, Henry MC, Dorny P, Meirvenne NV. [Prevalence of *Toxoplasma* *gondii* and *Toxocara* *cati* infections in cats in the Antwerp urban area.](http://ovidsp.tx.ovid.com/sp-3.31.1b/ovidweb.cgi?&S=IIPAFPDDHLDDEIPFNCEKOBGCCILLAA00&Complete+Reference=S.sh.69|37|1)  Annales de Medecine Veterinaire; 1985;129(6):433-440
210. Arene FOI. The prevalence and public health significance of *Toxoplasma gondii* in domestic cats in the Niger Delta. Public Health. 1984;98(6):333-5.
211. Ayinmode AB, Oluwayelu DO, Babalola ET, Lawani MA. Serologic survey of *Toxoplasma gondii* antibodies in cats (*Felis catus*) sold at live animal markets in Southwestern Nigeria. Bulgarian Journal of Veterinary Medicine 2017;20(1):58-64.
212. Lamaj S, Dhamo G, Dova, I. [*Toxoplasma gondii* infection in cats from southwest areas of Albania.](http://ovidsp.tx.ovid.com/sp-3.31.1b/ovidweb.cgi?&S=IIPAFPDDHLDDEIPFNCEKOBGCCILLAA00&Complete+Reference=S.sh.69|40|1)  Albanian Journal of Agricultural Sciences 2015;14(4):408-413
213. Fuh YB, Liao AT, Pong YM, Tung MMY, Fei CY, Lin DS. Survey of *Toxoplasma gondii* in Taipei: Livestock Meats, Internal Organs, Cat and Dog Sera. Thai J Vet Med. 2013;43(1): 15-21
214. Millan J, Cabezon O, Pabon M, Dubey JP, Almeria S. [Seroprevalence of *Toxoplasma gondii* and *Neospora caninum* in feral cats (*Felis silvestris catus*) in Majorca, Balearic Islands, Spain.](http://ovidsp.tx.ovid.com/sp-3.31.1b/ovidweb.cgi?&S=IIPAFPDDHLDDEIPFNCEKOBGCCILLAA00&Complete+Reference=S.sh.69|48|1) Vet Parasitol. 2009;165(3/4):323-326
215. Vanwormer E, Conrad PA, Miller MA, Melli AC, Carpenter TE, Mazet JA*. Toxoplasma gondii*, source to sea: higher contribution of domestic felids to terrestrial parasite loading despite lower infection prevalence. Ecohealth. 2013;10(3):277-89
216. Spada E, Proverbio D, Pepa A, della Domenichini G, Giorgi GB, de Traldi G, Ferro E. [Prevalence of faecal-borne parasites in colony stray cats in northern Italy.](http://ovidsp.tx.ovid.com/sp-3.31.1b/ovidweb.cgi?&S=IIPAFPDDHLDDEIPFNCEKOBGCCILLAA00&Complete+Reference=S.sh.69|64|1)  Journal of Feline Medicine and Surgery 2013;15(8):672-677
217. Hotea I, Colibar O, Ilie MS, Imre K, Imre M, Tirziu E, Seres M, Darabus GH. [*Toxoplasma gondii* infection in swine and cats from Timis County rural areas.](http://ovidsp.tx.ovid.com/sp-3.31.1b/ovidweb.cgi?&S=IIPAFPDDHLDDEIPFNCEKOBGCCILLAA00&Complete+Reference=S.sh.69|71|1)  Medicina Veterinara 2017;50(2):115-122
218. Verma SK, Minicucci L, Murphy D, Carstensen M, Humpal C, Wolf P, Calero-Bernal R, Cerqueira-Cézar CK, Kwok OC, Su C, Hill D, Dubey JP. Antibody Detection and Molecular Characterization of *Toxoplasma gondii* from Bobcats (*Lynx rufus*), Domestic Cats (*Felis catus*), and Wildlife from Minnesota, USA. J Eukaryot Microbiol. 2016;63(5):567-71
219. Fancourt BA, Jackson RB. Regional seroprevalence of Toxoplasma gondii antibodies in feral and stray cats (*Felis catus*) from Tasmania. Australian Journal of Zoology 2014;62(4):272-283
220. Hotea I, Oprescu I, Ilie MS, Imre K, Imre M, Darabus G. [Seroprevalence of *Toxoplasma gondii* infection in cats and sheep in Arad County.](http://ovidsp.tx.ovid.com/sp-3.31.1b/ovidweb.cgi?&S=IIPAFPDDHLDDEIPFNCEKOBGCCILLAA00&Complete+Reference=S.sh.69|80|1)  Medicina Veterinara 2011;44(1):63-68
221. Deeb BJ, Sufan MM, DiGiacomo RF. [*Toxoplasma gondii* infection of cats in Beirut, Lebanon.](http://ovidsp.tx.ovid.com/sp-3.31.1b/ovidweb.cgi?&S=IIPAFPDDHLDDEIPFNCEKOBGCCILLAA00&Complete+Reference=S.sh.69|84|1) Journal of Tropical Medicine and Hygiene 1985;88(5):301-306
222. Boch J, Walter D. [Four species of coccidia (*Isospora, Toxoplasma, Sarcocystis*) of cats in South Germany.](http://ovidsp.tx.ovid.com/sp-3.31.1b/ovidweb.cgi?&S=IIPAFPDDHLDDEIPFNCEKOBGCCILLAA00&Complete+Reference=S.sh.69|85|1) Tierarztliche Umschau 1979;34(11):749-752.
223. Unbehauen I. [Prevalence of intestinal parasites in cats in the Lubeck area.](http://ovidsp.tx.ovid.com/sp-3.31.1b/ovidweb.cgi?&S=IIPAFPDDHLDDEIPFNCEKOBGCCILLAA00&Complete+Reference=S.sh.69|90|1) [Germany] 1991:106 pp. 28 pp. of ref. [Thesis]
224. Andrade AC, de S, Bittencourt LHF, de B, Godoi NFC, Libardi KA, Weschenfelder DRS, Picolotto G, de CGP. [Prevalence of antibodies to *Toxoplasma gondii* in felines that go into veterinary hospitals and clinics in Cascavel, Parana, Brazil.](http://ovidsp.tx.ovid.com/sp-3.31.1b/ovidweb.cgi?&S=IIPAFPDDHLDDEIPFNCEKOBGCCILLAA00&Complete+Reference=S.sh.69|94|1) Arquivos de Ciencias Veterinarias e Zoologia da UNIPAR 2015;18(4):221-224
225. Childs JE, Seegar WS. [Epidemiologic observations on infection with *Toxoplasma gondii* in three species of urban mammals from Baltimore, Maryland, USA.](http://ovidsp.tx.ovid.com/sp-3.31.1b/ovidweb.cgi?&S=IIPAFPDDHLDDEIPFNCEKOBGCCILLAA00&Complete+Reference=S.sh.69|154|1)  International Journal of Zoonoses 1986;13(4):249-261.
226. Coman BJ, Jones EH, Westbury HA. [Protozoan and viral infections of feral cats.](http://ovidsp.tx.ovid.com/sp-3.31.1b/ovidweb.cgi?&S=IIPAFPDDHLDDEIPFNCEKOBGCCILLAA00&Complete+Reference=S.sh.69|159|1)  Australian Veterinary Journal; 1981. 57(7):319-323.
227. Hotea I, Ilie MS, Imre M, Sorescu D, Colibar O, Tirziu E, Seres M, Darabus G. [Prevalence of *Toxoplasma gondii* and intestinal parasites in stray and household cats in Western Romania.](http://ovidsp.tx.ovid.com/sp-3.31.1b/ovidweb.cgi?&S=IIPAFPDDHLDDEIPFNCEKOBGCCILLAA00&Complete+Reference=S.sh.69|374|1)  Medicina Veterinara 2013;46(3):85-90.
228. Meunier V, Jourda S, Deville M, Guillot J. Prevalence of anti-*Toxoplasma* *gondii* antibodies in serum and aqueous humor samples from cats with uveitis or systemic diseases in France. Vet Parasitol. 2006;138(3-4):362-5
229. Gethings PM, Stephens GL, Wills JM, Howard P, Balfour AH, Wright AI, Morgan KL. [Prevalence of *Chlamydia, Toxoplasma, Toxocara* and ringworm in farm cats in south-west England.](http://ovidsp.tx.ovid.com/sp-3.31.1b/ovidweb.cgi?&S=IIPAFPDDHLDDEIPFNCEKOBGCCILLAA00&Complete+Reference=S.sh.69|390|1)  Veterinary Record 1987;121(10):213-216.
230. Riemann, H. P. Kaneko, J. J. Haghighi, S. Behymer, D. E. Franti, C. E. Ruppanner, R. [The prevalence of antibodies against *Toxoplasma gondii* among hospitalized [cat, dog, horse, cattle] animals and stray dogs. Canadian Journal of Comparative Medicine 1978;42(4):407-413.](http://ovidsp.tx.ovid.com/sp-3.31.1b/ovidweb.cgi?&S=IIPAFPDDHLDDEIPFNCEKOBGCCILLAA00&Complete+Reference=S.sh.69|402|1)
231. Khodaverdi M, Razmi Gh. A serological and parasitological study of *Toxoplasma gondii* infection in stray cats of Mashhad, Khorasan Razavi province, Iran. Veterinary Research Forum 2019;10(2):119-123
232. Mohammed OB, Omar OI, Elamin EA, Bushara HO, Omer SA, Alagaili AN. Seroprevalence of *Toxoplasma gondii* in household and stray cats of Riyadh, Saudi Arabia. Vet Ital. 2019;55(3):241-245.
233. Watson AD, Farrow BR, McDonald PJ. Prevalence of *Toxoplasma gondii* antibodies in pet dogs and cats. Aust Vet J. 1982;58(5):213-4
234. Souza LZ, Rodrigues RGA, Oliveira DAD, de Roman JL, Zabott MV, Pinto SB, Bittencourt LHF, Oyafuso MK. [*Toxoplasma gondii* seroprevalence in cats in the city of Palotina, Parana, Brazil. [Portuguese].](http://ovidsp.tx.ovid.com/sp-3.31.1b/ovidweb.cgi?&S=IIPAFPDDHLDDEIPFNCEKOBGCCILLAA00&Complete+Reference=S.sh.69|409|1) Arquivos de Ciencias Veterinarias e Zoologia da UNIPAR 2017;20(3):123-126.
235. Mosallanejad B, Hamidinejat H, Shapouri MRS, Ghaleh FR. [A comparison between serological and molecular tests in diagnosis of *Toxoplasma gondii* infection among stray cats in Ahvaz, southwestern Iran.](http://ovidsp.tx.ovid.com/sp-3.31.1b/ovidweb.cgi?&S=IIPAFPDDHLDDEIPFNCEKOBGCCILLAA00&Complete+Reference=S.sh.69|411|1)  Archives of Razi Institute 2017;72(2):105-112.
236. Souza SF, de Medeiros L dos S, Belfort A de S, Cordeiro ALL, Federle M, de Souza AP, de Moura AB*.* [*Toxoplasma gondii* antibodies in domiciled cats from Rio Branco municipality, Acre state, Brazil.](http://ovidsp.tx.ovid.com/sp-3.31.1b/ovidweb.cgi?&S=IIPAFPDDHLDDEIPFNCEKOBGCCILLAA00&Complete+Reference=S.sh.69|418|1)  Semina: Ciencias Agrarias (Londrina) 2015;36(6):3757-3762
237. Liu QX, Wang Sh, Wang LQ, Xing J, Geo WJ, Liu GF, Zhao B, Zhang HB, Gao LH. Seroprevalence of *Toxoplasma gondii* infection in dogs and cats in Zhenjiang City, Eastern China. Asian Pacific Journal of Tropical Biomedicine 2014;4(9):725-728
238. Caldart ET, Constantino C, Pasquali AKS, Benitez AN, Hamada FN, Dias RCF, Rorato-Nascimento AM, Marana ERM, Navarro IT, Mascarenhas NMF, Freitas JC, Freire RL. [Zoonosis in dogs and cats attended by the Birth Control Project: *Toxoplasma gondii*, *Leishmania* spp. and *Leptospira* spp., serodiagnosis and epidemiology.](http://ovidsp.tx.ovid.com/sp-3.31.1b/ovidweb.cgi?&S=IIPAFPDDHLDDEIPFNCEKOBGCCILLAA00&Complete+Reference=S.sh.69|421|1)  Semina: Ciencias Agrarias (Londrina) 2015;36(1):253-265
239. Silaghi C, Knaus M, Rapti D, Kusi I, Shukullari E, Hamel D, Pfister K, Rehbein S. Survey of *Toxoplasma gondii* and *Neospora caninum*, haemotropic mycoplasmas and other arthropod-borne pathogens in cats from Albania. Parasit Vectors. 2014;7:62
240. Park HJ, Lee SE, Hong SH, Lee WJ, Seo KW, Song KH. Seroprevalence of *Toxoplasma gondii* and *Bartonella henselase* infection in stray cats of the Daejeon City, Korea. Korean J Vet Res. 2014;54(2):87-89
241. Arunvipas P, Jittapalapong S, Inpankaew T, Pinyopanuwat N, Chimnoi W, Maruyama S. Seroprevalence and risk factors influenced transmission of *Toxoplasma gondii* in dogs and cats in dairy farms in Western Thailand. African Journal of Agricultural Research 2013;8(7):591-595
242. Al-Kappany YM, Lappin MR, Kwok OC, Abu-Elwafa SA, Hilali M, Dubey JP. Seroprevalence of *Toxoplasma gondii* and concurrent *Bartonella* spp., feline immunodeficiency virus, feline leukemia virus, and *Dirofilaria immitis* infections in Egyptian cats. J Parasitol. 2011;97(2):256-8
243. Al-Mohammed HI. Seroprevalence of *Toxoplasma gondii* Infection in Cats, Dogs and Ruminant Animals in Al-Ahsa Area in Saudi Arabia. Research Journal of Medical Sciences 2011;5(4):190-192.
244. Hosono H, Iito Sh, Kono H, Xuan X. Seroprevalence of *Toxoplasma gondii* in Cats and Pigs from Thua Thien Hue Province in Vietnam. Journal of Veterinary Epidemiology 2009;13(2):100-106
245. Zhang H, Zhou DH, Zhou P, Lun ZR, Chen XG, Lin RQ, Yuan ZG, Zhu XQ. Seroprevalence of *Toxoplasma gondii* infection in stray and household cats in Guangzhou, China. Zoonoses Public Health. 2009;56(9-10):502-5
246. Oi M, Yoshikawa S, Maruyama S, Nogami S. Comparison of *Toxoplasma gondii* Seroprevalence in Shelter Cats and Dogs during 1999-2001 and 2009-2011 in Tokyo, Japan. PLoS One. 2015;10(8):e0135956
247. Fredebaugh SL, Mateus-Pinilla NE, McAllister M, Warner RE, Weng HY. Prevalence of antibody to *Toxoplasma gondii* in terrestrial wildlife in a natural area. J Wildl Dis. 2011;47(2):381-92.
248. Tizard I R, Harmeson J, Lai C H. The prevalence of serum antibodies to *Toxoplasma gondii* in Ontario mammals. Can J Comp Med. 1978; 42(2): 177–183.
249. Franti CE, Riemann HP, Behymer DE, Suther D, Howarth JA, Ruppanner R. Prevalence of *Toxoplasma gondii* antibodies in wild and domestic animals in northern California. J Am Vet Med Assoc. 1976;169(9):901-6
250. Scorza AV, Lappin MR. Prevalence of Selected Zoonotic and Vector-Borne Agents in Dogs and Cats on the Pine Ridge Reservation. Vet Sci. 2017; 4(3): 43.
251. Levy JK, Lappin MR, Glaser AL, Birkenheuer AJ, Anderson, TC, Edinboro CH. [Prevalence of infectious diseases in cats and dogs rescued following Hurricane Katrina.](http://ovidsp.tx.ovid.com/sp-3.31.1b/ovidweb.cgi?&S=IIPAFPDDHLDDEIPFNCEKOBGCCILLAA00&Complete+Reference=S.sh.69|485|1)  Journal of the American Veterinary Medical Association 2011;238(3):311-317.
252. Yamaguchi N, Macdonald DW, Passanisi WC, Harbour DA, Hopper CD. Parasite prevalence in free-ranging farm cats, *Felis silvestris catus*. Epidemiol Infect. 1996;116(2):217–223
253. Lin Sh, Zhichung L, Biaucheng Z, Huayuan Y. Prevalence of *Toxoplasma gondii* infection in man and animals in Guangdong, People's Republic of China. Vet Parasitol. 1990; 34(4): 357-360
254. Feitosa TF, Vilela VLR, Dantas ES, Souto DVO, Pena HFJ, Athayde ACR, Azevedo SS. [*Toxoplasma gondii* and *Neospora caninum* in domestic cats from the Brazilian semi-arid: seroprevalence and risk factors.](http://ovidsp.tx.ovid.com/sp-3.31.1b/ovidweb.cgi?&S=IIPAFPDDHLDDEIPFNCEKOBGCCILLAA00&Complete+Reference=S.sh.69|505|1)  Arq. Bras. Med. Vet. Zootec. 2014;66(4):1060-1066
255. Jimenez-Coello M, Acosta-Viana KY, Guzman-Marin M, Gutierrez-Ruiz EJ, Rodrigues-Vivas RI, Bolio-Gonzalez MEB, Ortega Pacheco A. The occurrence of *Toxoplasma gondii* antibodies in backyard pigs and cats from an endemic tropical area of Mexico. Tropical and Subtropical Agroecosystems 2013;16(1):89-92
256. Papini R, Sbrana C, Rosa B, Saturni AM, Sorrention AM, Cerretani M, Raffaelli G, Guidi G. Serological survey of *Toxoplasma gondii* infections in stray cats from Italy. Revue de medecine veterinaire 2006;157(4):193-196
257. Ortolani ES, Gennari SM, Pinheiro SR, Rodrigues AAR, Chiebao DP, Soares RM. [Prevalence of anti-*Toxoplasma gondii* antibodies in animals from Krucutu and Morro da Saudade Indian settlements in the municipality of Sao Paulo, Brazil [Portuguese].](http://ovidsp.tx.ovid.com/sp-3.31.1b/ovidweb.cgi?&S=IIPAFPDDHLDDEIPFNCEKOBGCCILLAA00&Complete+Reference=S.sh.69|511|1) Veterinaria e Zootecnia 2005;12(1/2):25-28.
258. Langoni H, da Silva AV, Cabral KG, Cunha ELP, Cutolo AA. [Prevalence of toxoplasmosis in cats from Sao Paulo and Parana States. [Portuguese].](http://ovidsp.tx.ovid.com/sp-3.31.1b/ovidweb.cgi?&S=IIPAFPDDHLDDEIPFNCEKOBGCCILLAA00&Complete+Reference=S.sh.69|515|1)  Brazilian Journal of Veterinary Research and Animal Science 2001; 38(1/6):243-244.
259. Shah AS, Raval SK, Bhadesiya CM, Modi DV, Parikh PV. [Prevalence of zoonotic toxoplasmosis and chlamydophilosis in domestic cats (*Felis catus*) of 3 urban cities of Gujarat state using a modified ELISA based diagnostic kit.](http://ovidsp.tx.ovid.com/sp-3.31.1b/ovidweb.cgi?&S=IIPAFPDDHLDDEIPFNCEKOBGCCILLAA00&Complete+Reference=S.sh.69|520|1)  Lifesciences Leaflets 2016;71:41-47.
260. Arraes-Santos AI, Araújo AC, Guimarães MF, Santos JR, Pena HFJ, Gennari SM, Azevedo SS, Labruna MB, Horta MC. Seroprevalence of anti-*Toxoplasma gondii* and anti-*Neospora* *caninum* antibodies in domestic mammals from two distinct regions in the semi-arid region of Northeastern Brazil. Vet Parasitol Reg Stud Reports. 2016;5:14-18
261. Waap H, Nunes T, Vaz Y, Leitao A. [Serological survey of *Toxoplasma gondii* and *Besnoitia besnoiti* in a wildlife conservation area in southern Portugal.](http://ovidsp.tx.ovid.com/sp-3.31.1b/ovidweb.cgi?&S=IIPAFPDDHLDDEIPFNCEKOBGCCILLAA00&Complete+Reference=S.sh.69|580|1)  Veterinary Parasitology: Regional Studies and Reports 2016;3/4:7-12.
262. Asgari Q, Mohammadpour I, Pirzad R, Kalantari M, Motazedian MH, Naderi Sh. Molecular and Serological Detection of *Toxoplasma gondii* in Stray Cats in Shiraz, South-central, Iran. Iran J Parasitol. 2018;13(3): 430-439
263. Darabus G, Afrenie M, Olariu RT, Ilie MS, Balint A, Hotea I. Epidemiological remarks on *Toxoplasma gondii* infection in Timişoara Zoo. 2011;Sci Parasitol 12(1):33-37
264. Roqueplo C, Halos L, Cabre O, Davoust B. [*Toxoplasma gondii* in wild and domestic animals from New Caledonia.](http://ovidsp.tx.ovid.com/sp-3.31.1b/ovidweb.cgi?&S=IIPAFPDDHLDDEIPFNCEKOBGCCILLAA00&Complete+Reference=S.sh.69|615|1) Parasite 2011;18(4):345-348.
265. Zia-Ali N, Fazaeli A, Khoramizadeh M, Ajzenberg D, Dardé M, Keshavarz-Valian H. Isolation and molecular characterization of *Toxoplasma gondii* strains from different hosts in Iran. Parasitol Res. 2007;101(1):111-5.
266. Hwang J, Gottdenker N, Oh DH, Lee H, Chun MS. Infections by pathogens with different transmission modes in feral cats from urban and rural areas of Korea. J Vet Sci. 2017;18(4):541-545
267. Gregory GG, Munday BL. [Internal parasites of feral cats from the Tasmanian Midlands and King Island.](http://ovidsp.tx.ovid.com/sp-3.31.1b/ovidweb.cgi?&S=IIPAFPDDHLDDEIPFNCEKOBGCCILLAA00&Complete+Reference=S.sh.69|671|1)  Australian Veterinary Journal 1976;52(7):317-320.
268. Suzan G, Ceballos G. [The role of feral mammals on wildlife infectious disease prevalence in two nature reserves within Mexico City limits.](http://ovidsp.tx.ovid.com/sp-3.31.1b/ovidweb.cgi?&S=IIPAFPDDHLDDEIPFNCEKOBGCCILLAA00&Complete+Reference=S.sh.69|678|1)  Journal of Zoo and Wildlife Medicine 2005;36(3):479-484.
