# Supplementary material for: Toxoplasma gondii infection in domestic and wild felids as public health concerns: a systematic review and meta-analysis
Source: Sci Rep. 2021 May 4;11:9509. doi: 10.1038/s41598-021-89031-8 (PMC8097069; doi:10.1038/s41598-021-89031-8)
Supplement: Supplementary file 5 — Supplementary Information 5. [file 41598_2021_89031_MOESM5_ESM.doc]

**Table S3.** Seroprevalence of anti-*Toxoplasma gondii* antibodies in wild felids (sorted by scientific name and publication date)^a^

| **Species** | **Location** | **Status** | **Test** | **Sample size** | **Positive (%)** | **Cut-off titer** | **No. with titers of** | | | | | | | **References** |
| --- | --- | --- | --- | --- | --- | --- | --- | --- | --- | --- | --- | --- | --- | --- |
|  |  |  |  |  |  |  | <10 | 16-20 | 25-40 | 50-64 | 128 | ≥256 | |  |
| **Lion** |  |  |  |  |  |  |  |  |  |  |  |  | |  |
| *Panthera leo* | USA (Florida) | Captive | ELISA | 2 | 2 (100) | 1:64 |  |  |  | 2 |  |  | | Lappin et al. 1991 |
| *Panthera leo* | Southern Africa | Free ranging | ELISA | 66 | 65 (98.0) | N.S. |  |  |  |  |  |  | | Spencer and Morkel 1993 |
| *Panthera leo* | Southern Africa | Free ranging | IFAT | 47 | 43 (91.2) | 1:50 |  |  |  | 37 |  | 6 | | Cheadle et al. 1999 |
| *Panthera leo* | Brazil | Captive | MAT | 27 | 14 (51.8) | 1:20 |  |  | 9 | 4 |  | 1 | | Silva et al. 2001 |
| *Panthera leo* | Botswana | Free ranging | IFAT | 53 | 49 (92.0) | 1:20 |  | 49 |  |  |  |  | | Penzhorn et al. 2002 |
| *Panthera leo* | South Africa | Free ranging | IFAT | 42 | 42 (100) | 1:20 |  | 42 |  |  |  |  | | Penzhorn et al. 2002 |
| *Panthera leo* | Zimbabwe | Free ranging | IFAT | 21 | 21 (100) | 1:20 |  |  |  |  |  |  | | Penzhorn et al. 2002 |
| *Panthera leo* | USA (Various areas) | Captive | IFAT | 10 | 8 (80.0) | 1:50 |  |  |  | 1 | 4 | 3 | | Spencer et al. 2003 |
| *Panthera leo* | Zimbabwe | Free ranging | MAT | 26 | 24 (92.3) | 1:25 |  |  | 24 |  |  |  | | Hove and Mukaratirwa 2005 |
| *Panthera leo* | Thailand | Captive | LAT | 7 | 1 (14.3) | 1:64 |  |  |  | 1 |  |  | | Thiangtum et al. 2006 |
| *Panthera leo* | Brazil | Captive | IFAT | 3 | 3 (100) | N.S. |  |  |  |  |  |  | | Rivetti et al. 2008 |
| *Panthera leo* | USA (Midwestern zoos) | Captive | MAT | 22 | 12 (54.5) | 1:25 |  |  | 12 |  |  |  | | de Camps et al. 2008 |
| *Panthera leo* | Brazil (São Paulo) | Captive^†^ | IFAT | 9 | 5 (55.5) | 1:40 |  |  |  |  |  |  | | Andre et al. 2010 |
| *Panthera leo* | Romania (Timişoara Zoo) | Captive | ELISA | 3 | 3 (100) | N.S. |  |  |  |  |  |  | | Darabus et al. 2011 |
| *Panthera leo* | Mexico (Mexico City) | Captive | MAT | 7 | 7 (100) | 1:25 |  |  |  | 3 |  | 2 | Alvarado-Esquivel et al. 2013 | |
| Panthera leo | China (Henan) | Captive | MAT | 6 | 6 (100) | 1:25 |  |  |  |  |  | 6 | | Yang et al. 2017 |
| Panthera leo | Italy | Captive | IFAT | 14 | 13 (93.0) | N.S. |  |  |  |  |  |  | | Markova et al. 2019 |
| *Panthera leo* | Mexico | Captive | ELISA | 8 | 8 (100) | N.S. |  |  |  |  |  |  | | Gomez-Rios et al. 2019 |
| *Panthera leo* | Germany | Captive | ELISA | 3 | 3 (100) | 1:10 |  |  |  |  |  |  | | Ferreira et al. 2019 |
| *Panthera leo* | Tanzania (Serengeti) | Free ranging | ELISA | 15 | 15 (100) | 1:10 |  |  |  |  |  |  | | Ferreira et al. 2019 |
| **Jaguar** |  |  |  |  |  |  |  |  |  |  |  |  | |  |
| *Panthera onca* | Brazil | Captive | MAT | 212 | 134 (63.2) | 1:20 |  |  |  |  |  |  | | Silva et al. 2001b |
| *Panthera onca* | USA (Various areas) | Captive | IFAT | 2 | 2 (100) | 1:50 |  |  |  | 2 |  |  | | Spencer et al. 2003 |
| *Panthera onca* | Thailand | Captive | LAT | 3 | 1 (33.3) | 1:100 |  |  |  | 1 |  |  | | Thiangtum et al. 2006 |
| *Panthera onca* | Brazil | Captive | MAT | 212 | 135 (64.0) | ≥1:20 |  |  |  |  |  |  | | Silva et al. 2007 |
| *Panthera onca* | USA (Midwestern zoos) | Captive | MAT | 1 | 1 (100) | 1:25 |  |  | 1 |  |  |  | | de Camps et al. 2008 |
| *Panthera onca* | Brazil | Captive | IFAT | 3 | 3 (100) | N.S. |  |  |  |  |  |  | | Rivetti et al. 2008 |
| *Panthera onca* | French Guiana | Free ranging | MAT | 1 | 1 (100) | 1:4000 |  |  |  |  |  | 1 | | Demar et al. 2008 |
| *Panthera onca* | Brazil (São Paulo) | Captive^†^ | IFAT | 13 | 11 (84.6) | 1:40 |  |  |  |  |  |  | | Andre et al., 2010 |
| *Panthera onca* | Mexico | Captive | ELISA | 12 | 12 (100) | N.S. |  |  |  |  |  |  | | Gomez-Rios et al. 2019 |
| **Jaguarundi** |  |  |  |  |  |  |  |  |  |  |  |  | |  |
| *Herpailurus yagouaroundi* | USA (Florida) | Captive | ELISA | 1 | 1 (100) | 1:64 |  |  |  |  |  |  | | Lappin et al. 1991 |

**Table 4.** Continued

| **Species** | | **Location** | **Status** | **Test** | **Sample size** | **Positive (%)** | **Cut-off titer** | **No. with titers of** | | | | | | | **References** |
| --- | --- | --- | --- | --- | --- | --- | --- | --- | --- | --- | --- | --- | --- | --- | --- |
|  |  |  |  |  |  |  |  | <10 | 16-20 | 25-40 | 50-64 | 128 | ≥256 | |  |
| *Herpailurus yagouaroundi* | | Brazil | Captive | MAT | 99 | 45 (45.4) | 1:20 |  | 15 | 29 |  |  | 1 | | Silva et al. 2001b |
| *Herpailurus yagouaroundi* | | Bolivia (Bolivian Chaco) | Free ranging | KELA | 1 | 1 (100) | N.S. |  |  |  |  |  |  | | Fiorello et al. 2006 |
| *Herpailurus yagouaroundi* | | Brazil | Captive | MAT | 99 | 46 (46.4) | ≥1:20 |  |  |  |  |  |  | | Silva et al. 2007 |
| *Herpailurus yagouaroundi* | | Brazil | Captive | IFAT | 1 | 1 (100) | N.S. |  |  |  |  |  |  | | Rivetti et al. 2008 |
| *Herpailurus yagouaroundi* | | Brazil (Southern) | Captive | MAT | 3 | 2 (66.6) | 1:16 |  |  |  |  |  |  | | Ullmann et al. 2010 |
| *Herpailurus yagouaroundi* | | Brazil (São Paulo) | Captive^†^ | IFAT | 25 | 10 (40.0) | 1:40 |  |  |  |  |  |  | | Andre et al. 2010 |
| *Herpailurus jagouaroundi* | | Mexico (Mexico City) | Captive | MAT | 2 | 1 (100) | 1:25 |  |  |  | 1 |  |  | | Alvarado-Esquivel et al. 2013 |
| *Herpailurus yagouaroundi* | | Europe | N.S. | IFAT | 9 | 5 (55.6) | N.S. |  |  |  |  |  |  | | Lucht et al. 2019 |
| *Herpailurus yagouaroundi* | | Mexico | Captive | ELISA | 2 | 2 (100) | N.S. |  |  |  |  |  |  | | Gomez-Rios et al. 2019 |
| **Ocelot** | |  |  |  |  |  |  |  |  |  |  |  |  | |  |
| *Leopardus pardalis* | | Brazil (São Paulo) | Captive | DT | 6 | 3 (50.0) | 1:256 |  |  |  |  |  |  | | Sogorb et al. 1977 |
| *Leopardus pardalis* | | Brazil | Captive | MAT | 168 | 97 (57.7) | 1:20 |  |  | 38 | 58 |  |  | | Silva et al. 2001b |
| *Leopardus pardalis* | | USA (Alabama) | Captive | IFAT | 1 | 1 (100) | 1:50 |  | 25 | 43 |  | 1 |  | | Spencer et al. 2003 |
| *Leopardus pardalis* | | Bolivia (Bolivian Chaco) | Free ranging | KELA | 10 | 10 (100) | N.S. |  |  |  |  |  |  | | Fiorello et al. 2006 |
| *Leopardus pardalis* | | Brazil | Captive | MAT | 168 | 97 (58.0) | ≥1:20 |  |  |  |  |  |  | | Silva et al. 2007 |
| *Leopardus pardalis* | | Brazil | Captive | IFAT | 5 | 4 (80.0) | N.S. |  |  |  |  |  |  | | Rivetti et al. 2008 |
| *Leopardus pardalis* | | Brazil (Southern) | Captive | MAT | 14 | 10 (71.4) | 1:16 |  |  |  |  |  |  | | Ullmann et al. 2010 |
| *Leopardus pardalis* | | Brazil (São Paulo) | Captive^†^ | IFAT | 42 | 28 (66.7) | 1:40 |  |  |  |  |  |  | | Andre et al. 2010 |
| *Leopardus pardalis* | | Mexico (Tamaulipas) | Free ranging | LAT | 26 | 18 (69.2) | ≥1:32 |  |  |  |  |  |  | | Rendon-Franco et al. 2012 |
| *Leopardus pardalis* | Mexico (Mexico City) | | Captive | MAT | 3 | 2 (66.6) | 1:25 |  |  |  |  |  | 1 | Alvarado-Esquivel et al. 2013 | |
| *Leopardus pardalis* | | Mexico | Captive | ELISA | 2 | 2 (100) | N.S. |  |  |  |  |  |  | | Gomez-Rios et al. 2019 |
| **Cougar** | |  |  |  |  |  |  |  |  |  |  |  |  | |  |
| *Puma concolor* | | USA (Florida) | Captive | ELISA | 6 | 5 (83.3) | 1:64 |  |  |  | 5 |  |  | | Lappin et al. 1991 |
| *Puma concolor* | | USA (Florida) | Captive | ELISA | 38 | 3 (7.9) | 1:48 |  |  |  | 5 |  |  | | Roelke et al. 1993 |
| *Puma concolor* | | USA (California) | Free ranging | LAT | 36 | 21 (58.3) | 1:32 |  |  | 21 |  |  |  | | Paul-Murphy et al.1994 |
| *Puma c. vancouverensis* | | Canada (Vancouver) | Free ranging | IHA | 5 | 5 (100) | 1:40 |  |  | 2 | 3 |  |  | | Stephen et al. 1996 |
| *Puma c. vancouverensis* | | Canada (Vancouver) | Free ranging | MAT | 12 | 11 (92.0) | 1:25 |  |  |  | 8 |  | 3 | | Aramini et al. 1998 |
| *Puma concolor* | | China (Shanghai) | Captive | ELISA | 1 | 1 (100) | 1:50 |  |  |  |  |  |  | | Zhang et al. 2000 |
| *Puma concolor* | | Brazil | Captive | MAT | 172 | 83 (48.3) | 1:20 |  |  | 41 | 42 |  |  | | Silva et al. 2001b |
| *Puma concolor* | | USA (California) | Captive | IFAT | 42 | 11 (26.2) | 1:50 |  |  |  | 2 | 4 | 4 | | Spencer et al. 2003 |
| *Puma concolor* | | USA (Various areas) | Captive | IFAT | 5 | 3 (60.0) | 1:50 |  |  |  |  | 3 |  | | Spencer et al. 2003 |
| *Puma concolor* | | Canada | Free ranging | LAT | 23 | 8 (34.8) | 1:64 |  |  |  | 8 |  |  | | Kikuchi et al. 2004 |
| *Puma concolor* | | Canada | Captive | ELISA | 15 | 1 (7.0) | N.S. |  |  |  |  |  |  | | Philippa et al. 2004 |

**Table 4.** Continued

| **Species** | **Location** | **Status** | **Test** | **Sample size** | **Positive (%)** | **Cut-off titer** | **No. with titers of** | | | | | | **References** |
| --- | --- | --- | --- | --- | --- | --- | --- | --- | --- | --- | --- | --- | --- |
|  |  |  |  |  |  |  | <10 | 16-20 | 25-40 | 50-64 | 128 | ≥256 |  |
| *Puma concolor* | Central & South America | Free ranging | LAT | 83 | 27 (32.5) | 1:64 |  |  |  |  |  |  | Kikuchi et al. 2004 |
| *Puma concolor* | Mexico | Free ranging | LAT | 12 | 2 (16.7) | 1:64 |  |  |  | 2 |  |  | Kikuchi et al. 2004 |
| *Puma concolor* | USA | Free ranging | LAT | 320 | 61 (19.1) | 1:64 |  |  |  | 61 |  |  | Kikuchi et al. 2004 |
| *Puma concolor* | Thailand^*^ | Captive | DT | 3 | 0 (0.0) | N.S. |  |  |  |  |  |  | Buddhirongawatr et al. 2006 |
| *Puma concolor* | Brazil | Captive | MAT | 172 | 83 (48.0) | ≥1:20 |  |  |  |  |  |  | Silva et al. 2007 |
| *Puma concolor* | Brazil | Captive | IFAT | 5 | 5 (100) | N.S. |  |  |  |  |  |  | Rivetti et al. 2008 |
| *Puma concolor* | USA (California) | Free ranging | IFAT | 26 | 24 (92.3) | 1:40 |  |  | 4 | 3 | 4 | 13 | Miller et al. 2008 |
| *Puma concolor* | USA (Midwestern zoos) | Captive | MAT | 8 | 5 (62.5) | 1:25 |  |  | 5 |  |  |  | de Camps et al. 2008 |
| *Puma concolor* | Brazil (São Paulo) | Captive^†^ | IFAT | 18 | 14 (77.8) | 1:40 |  |  |  |  |  |  | Andre et al. 2010 |
| *Puma concolor* | USA (California & Colorado) | Free ranging | ELISA | 202 | 151 (75.0) | 1:52 |  |  |  |  |  |  | Bevins et al. 2012 |
| *Puma concolor* | USA (California) | Free ranging | IFAT | 72 | 58 (80.6) | N.S. |  |  |  |  |  |  | VanWormer et al. 2013 |
| *Puma concolor* | Mexico (Mexico City) | Captive | MAT | 4 | 4 (100) | 1:25 |  |  | 1 | 1 | 1 | 1 | Alvarado-Esquivel et al. 2013 |
| *Puma concolor* | Mexico | Captive | ELISA | 2 | 2 (100) | N.S. |  |  |  |  |  |  | Gomez-Rios et al. 2019 |
| **Leopard** |  |  |  |  |  |  |  |  |  |  |  |  |  |
| *Panthera pardus* | USA (Florida) | Captive | ELISA | 3 | 2 (66.6) | 1:64 |  |  |  |  |  |  | Lappin et al. 1991 |
| *Panthera pardus* | Southern Africa | Captive | IFAT | 4 | 3 (75.0) | 1:50 |  |  |  |  |  |  | Cheadle et al. 1999 |
| *Panthera pardus* | China (Shanghai) | Captive | MAT | 1 | 1 (100) | 1:20 |  |  |  |  |  | 1 | Zhang et al. 2000 |
| *P. pardus sakicolor* | China (Shanghai) | Captive | MAT | 1 | 1 (100) | 1:20 |  |  |  |  |  | 1 | Zhang et al. 2000 |
| *Panthera pardus* | Brazil | Captive | MAT | 3 | 3 (100) | 1:20 |  |  |  | 2 |  | 1 | Silva et al. 2001 |
| *Panthera pardus* | Botswana | Free ranging | IFAT | 1 | 1 (100) | 1:20 |  |  |  |  |  |  | Penzhorn et al. 2002 |
| *Panthera pardus* | South Africa | Free ranging | IFAT | 7 | 6 (86.0) | 1:20 |  | 6 |  |  |  |  | Penzhorn et al. 2002 |
| *Panthera pardus* | USA (California) | Captive | IFAT | 1 | 1 (100) | 1:50 |  |  |  |  | 1 |  | Spencer et al. 2003 |
| *Panthera pardus* | Thailand | Captive | LAT | 19 | 3 (15.8) | 1:64 |  |  |  | 3 |  |  | Thiangtum et al. 2006 |
| *Panthera pardus* | Thailand^*^ | Captive | DT | 1 | 1 (100) | 1:8 |  |  |  |  |  |  | Buddhirongawatr et al. 2006 |
| *Panthera pardus* | USA (Midwestern zoos) | Captive | MAT | 1 | 1 (100) | 1:25 |  |  | 1 |  |  |  | de Camps et al. 2008 |
| *Panthera pardus nimr* | United Arab Emirates○ | Captive | MAT | 7 | 6 (85.7) | 1:25 |  |  |  | 2 |  | 2 | Dubey et al. 2010 |
| *Panthera pardus* | Brazil (São Paulo) | Captive^†^ | IFAT | 1 | 1 (100) | 1:40 |  |  |  |  |  |  | Andre et al. 2010 |
| *Panthera pardus* | Mexico (Mexico City) | Captive | MAT | 5 | 5 (100) | 1:25 | 3 |  |  |  |  |  | Alvarado-Esquivel et al. 2013 |
| *Panthera pardus* | China (Jiangxi) | Captive | IHA | 3 | 0 (0.0) | ≥1:64 |  |  |  |  |  |  | Luo et al. 2017 |
| *Panthera pardus* | Mexico | Captive | ELISA | 1 | 1 (100) | N.S. |  |  |  |  |  |  | Gomez-Rios et al. 2019 |
| Panthera p. orientalis | Russia (Southern Primorye) | Free ranging | ELISA | 8 | 0 (0.0) | N.S. |  |  |  |  |  |  | Naidenko et al. 2019 |
| **Tiger** |  |  |  |  |  |  |  |  |  |  |  |  |  |
| *Panthera tigris altaica* | Belgium (Limburg) | Captive | MAT | 1 | 1 (100) | N.S. |  |  |  |  | 1 |  | Dorny and Fransen 1989 |

**Table 4.** Continued

| **Species** | **Location** | **Status** | **Test** | **Sample size** | **Positive (%)** | **Cut-off titer** | **No. with titers of** | | | | | | **References** |
| --- | --- | --- | --- | --- | --- | --- | --- | --- | --- | --- | --- | --- | --- |
|  |  |  |  |  |  |  | <10 | 16-20 | 25-40 | 50-64 | 128 | ≥256 |  |
| *Panthera tigris* | USA (Florida) | Captive | ELISA | 4 | 3 (75.0) | 1:64 |  |  |  | 3 |  |  | Lappin et al. 1991 |
| *Panthera t. amoyensis* | China (Shanghai) | Captive | MAT | 7 | 6 (85.7) | 1:20 |  | 2 | 2 |  |  | 1 | Zhang et al. 2000 |
| *P. tigris tigris* | China (Shanghai) | Captive | MAT | 2 | 2 (100) | 1:20 |  |  |  |  |  | 2 | Zhang et al. 2000 |
| *Panthera tigris*^a^ | Brazil | Captive | MAT | 2 | 2 (100) | 1:20 |  |  | 1 | 1 |  |  | Silva et al. 2001 |
| *Panthera tigris* | USA (Various areas) | Captive | IFAT | 11 | 7 (63.6) | 1:50 |  |  |  | 3 | 1 | 3 | Spencer et al. 2003 |
| *Panthera tigris* | Thailand | Captive | LAT | 18 | 5 (27.8) | 1:64 |  |  |  | 5 |  |  | Thiangtum et al. 2006 |
| *Panthera tigris* | Thailand^*^ | Captive | DT | 6 | 3 (50.0) | 1:16 |  |  |  |  |  |  | Buddhirongawatr et al. 2006 |
| *Panthera tigris* | Brazil | Captive | IFAT | 2 | 2 (100) | 1:100 |  |  |  |  |  |  | Rivetti et al. 2008 |
| *Panthera t. altaica* | USA (Midwestern zoos) | Captive | MAT | 18 | 5 (27.8) | 1:25 |  |  |  |  |  |  | de Camps et al. 2008 |
| *Panthera tigris* | Brazil (São Paulo) | Captive^†^ | IFAT | 6 | 4 (66.7) | 1:40 |  |  |  |  |  |  | Andre et al. 2010 |
| *Panthera t. sumatrae* | Mexico (Mexico City) | Captive | MAT | 2 | 2 (100) | 1:25 |  |  |  |  |  | 1 | Alvarado-Esquivel et al. 2013 |
| *Panthera tigris* | Mexico (Mexico City) | Captive | MAT | 3 | 2 (66.6) | 1:25 |  |  |  |  |  | 2 | Alvarado-Esquivel et al. 2013 |
| Panthera tigris | China (Henan) | Captive | MAT | 3 | 2 (66.7) | 1:25 |  |  |  | 1 |  | 1 | Yang et al. 2017 |
| *Panthera tigris* | Mexico | Captive | ELISA | 9 | 9 (100) | N.S. |  |  |  |  |  |  | Gomez-Rios et al. 2019 |
| *Panthera t. altaica* | Russia (Southern Primorye) | Free ranging | ELISA | 18 | 7 (38.9) | N.S. |  |  |  |  |  |  | Naidenko et al. 2019 |
| **Geoffroy's cat** |  |  |  |  |  |  |  |  |  |  |  |  |  |
| *Leopardus geoffroyi* | Brazil | Captive | MAT | 12 | 9 (75.0) | 1:20 |  |  | 1 | 8 |  |  | Silva et al. 2001b |
| *Leopardus geoffroyi* | Brazil | Captive | MAT | 12 | 10 (83.0) | ≥1:20 |  |  |  |  |  |  | Silva et al. 2007 |
| *Leopardus geoffroyi* | Bolivia (Bolivian Chaco) | Free ranging | KELA | 8 | 2 (25.0) | N.S. |  |  |  |  |  |  | Fiorello et al. 2006 |
| *Leopardus geoffroyi* | Brazil (Southern) | Captive | MAT | 1 | 1 (100) | 1:16 |  |  |  |  |  |  | Ullmann et al. 2010 |
| *Leopardus geoffroyi* | Europe | N.S. | IFAT | 33 | 16 (48.5) | N.S. |  |  |  |  |  |  | Lucht et al. 2019 |
| **Oncilla** |  |  |  |  |  |  |  |  |  |  |  |  |  |
| *Leopardus tigrinus* | Brazil (São Paulo) | Captive | DT | 9 | 6 (66.6) | 1:256 |  |  |  |  |  |  | Sogorb et al. 1977 |
| *Leopardus tigrinus* | Brazil | Captive | MAT | 131 | 68 (51.9) | 1:20 |  | 9 | 26 |  |  |  | Silva et al. 2001b |
| *Leopardus tigrinus* | Brazil | Captive | MAT | 131 | 66 (50.0) | ≥1:20 |  |  |  |  |  |  | Silva et al. 2007 |
| *Leopardus tigrinus* | Brazil (Southern) | Captive | MAT | 22 | 15 (68.1) | 1:16 |  |  |  |  |  |  | Ullmann et al. 2010 |
| *Leopardus tigrinus* | Brazil (São Paulo) | Captive^†^ | IFAT | 35 | 22 (62.8) | 1:40 |  |  |  |  |  |  | Andre et al. 2010 |
| *Leopardus tigrinus* | Mexico (Mexico City) | Captive | MAT | 2 | 0 (0.0) | 1:25 |  |  |  |  |  |  | Alvarado-Esquivel et al. 2013 |
| *Leopardus tigrinus* | Brazil (São Paulo) | Free ranging | MB | 3 | 3 (100) | - |  |  |  |  |  |  | Vitaliano et al. 2014 |
| *Leopardus tigrinus* | Europe | N.S. | IFAT | 9 | 6 (66.7) | N.S. |  |  |  |  |  |  | Lucht et al. 2019 |
| *Leopardus tigrinus* | Mexico | Captive | ELISA | 3 | 3 (100) | N.S. |  |  |  |  |  |  | Gomez-Rios et al. 2019 |
| **Margay** |  |  |  |  |  |  |  |  |  |  |  |  |  |
| *Leopardus wiedii* | Brazil | Captive | MAT | 66 | 36 (54.5) | 1:20 |  |  |  |  |  |  | Silva et al. 2001b |

**Table 4.** Continued

| **Species** | **Location** | **Status** | **Test** | **Sample size** | **Positive (%)** | **Cut-off titer** | **No. with titers of** | | | | | | **References** |
| --- | --- | --- | --- | --- | --- | --- | --- | --- | --- | --- | --- | --- | --- |
|  |  |  |  |  |  |  | <10 | 16-20 | 25-40 | 50-64 | 128 | ≥256 |  |
| *Leopardus wiedii* | Brazil | Captive | MAT | 63 | 34 (54.0) | ≥1:20 |  |  |  |  |  |  | Silva et al. 2007 |
| *Leopardus wiedii* | Brazil (São Paulo) | Captive^†^ | IFAT | 4 | 4 (100) | 1:40 |  |  |  |  |  |  | Andre et al. 2010 |
| *Leopardus wiedii* | Brazil (Southern) | Captive | MAT | 17 | 10 (58.8) | 1:16 |  |  |  |  |  |  | Ullmann et al. 2010 |
| *Leopardus wiedii* | Europe | N.S. | IFAT | 19 | 9 (47.4) | N.S. |  |  |  |  |  |  | Lucht et al. 2019 |
| **Caracal** |  |  |  |  |  |  |  |  |  |  |  |  |  |
| *Caracal caracal* | China (Shanghai) | Captive | ELISA | 1 | 1 (100) | 1:50 |  |  |  |  |  |  | Zhang et al. 2000 |
| *Caracal caracal* | USA (California) | Captive | IFA | 1 | 1 (100) | 1:50 |  |  |  |  | 1 |  | Spencer et al. 2003 |
| *Caracal caracal* | USA (Midwestern zoos) | Captive | MAT | 4 | 2 (50.0) | 1:25 |  |  |  |  |  |  | de Camps et al. 2008 |
| *Caracal caracal* | Brazil (São Paulo) | Captive^†^ | IFAT | 1 | 0 (0.0) | 1:40 |  |  |  |  |  |  | Andre et al. 2010 |
| *Caracal c. schmitsi* | United Arab Emirates ○ | Captive | MAT | 6 | 5 (83.3) | 1:25 |  |  |  |  |  | 1 | Dubey et al. 2010 |
| *Caracal c. algira* | United Arab Emirates ○ | Captive | MAT | 1 | 1 (100) | 1:25 |  |  |  |  |  |  | Dubey et al. 2010 |
| *Caracal caracal* | Mexico | Captive | ELISA | 1 | 1 (100) | N.S. |  |  |  |  |  |  | Gomez-Rios et al. 2019 |
| **Snow leopard** |  |  |  |  |  |  |  |  |  |  |  |  |  |
| *Panthera uncia* | Thailand | Captive | LAT | 1 | 1 (100) | 1:64 |  |  |  | 1 |  |  | Thiangtum et al. 2006 |
| *Panthera uncia* | USA (Midwestern zoos) | Captive | MAT | 14 | 5 (35.7) | 1:25 |  |  |  |  |  |  | de Camps et al. 2008 |
| **Eurasian lynx** |  |  |  |  |  |  |  |  |  |  |  |  |  |
| *Lynx lynx* | Brazil | Captive | MAT | 1 | 1 (100) | 1:20 |  |  |  | 1 |  |  | Silva et al. 2001a |
| *Lynx lynx* | Canada (Quebec) | Free ranging | MAT | 106 | 46 (43.4) | 1:25 |  |  | 12 | 35 |  |  | Labelle et al. 2001 |
| *Lynx lynx* | USA (Alaska) | Free ranging | MAT | 255 | 39 (15.3) | 1:25 |  |  | 10 | 10 | 9 | 10 | Zarnke et al. 2001 |
| *Lynx lynx* | Canada | Captive | ELISA | 5 | 1 (20.0) | N.S. |  |  | 1 |  |  |  | Philippa et al. 2004 |
| *Lynx lynx* | Sweden | Free ranging | MAT | 207 | 155 (75.0) | 1:40 |  |  | 29 | 23 |  | 88 | Ryser-Degiorgis et al. 2006 |
| Lynx lynx | Russia (Southern Primorye) | Free ranging | ELISA | 5 | 1 (20.0) |  |  |  |  |  |  |  | Naidenko et al. 2019 |
| **Bobcat** |  |  |  |  |  |  |  |  |  |  |  |  |  |
| *Lynx rufus* | USA (New Mexico) | Free ranging | DT | 27 | 12 (44.0) | ≥1:32 |  |  |  |  |  |  | Marchiondo et al. 1976 |
| *Lynx rufus* | USA (Arizona) | Free ranging | DT | 1 | 0 (0.0) | ≥1:32 |  |  |  |  |  |  | Marchiondo et al. 1976 |
| *Lynx rufus* | USA (California) | Free ranging | IHA | 86 | 58 (68.0) | N.S. |  |  |  |  |  |  | Franti et al. 1976 |
| *Lynx rufus* | USA (Virginia, Georgia) | Free ranging | IHA | 150 | 27 (18.0) | 1:16 |  |  |  |  |  |  | Oertley and Walls 1980 |
| *Lynx rufus* | USA (Kansas) | Free ranging | DT | 2 | 1 (50.0) | 1:8 | 1 |  |  |  |  |  | Smith and Frenkel 1995 |
| *Lynx rufus* | Canada (Quebec) | Free ranging | MAT | 10 | 4 (40.0) | 1:25 |  |  | 1 | 3 |  |  | Labelle et al. 2001 |
| *Lynx rufus* | USA (Various areas) | Captive | IFA | 3 | 1 (33.3) | 1:50 |  |  |  |  | 1 |  | Spencer et al. 2003 |
| *Lynx rufus* | Mexico | Free ranging | LAT | 6 | 4 (66.6) | 1:64 |  |  |  | 4 |  |  | Kikuchi et al. 2004 |
| *Lynx rufus* | USA (California) | Free ranging | LAT | 52 | 26 (50.0) | 1:64 |  |  |  | 26 |  |  | Kikuchi et al. 2004 |
| *Lynx rufus* | USA (California) | Free ranging | LAT | 25 | 22 (88.0) | 1:64 |  |  | 22 |  |  |  | Riley et al. 2004 |

**Table 4.** Continued

| **Species** | **Location** | **Status** | **Test** | **Sample size** | **Positive (%)** | **Cut-off titer** | **No. with titers of** | | | | | | **References** |
| --- | --- | --- | --- | --- | --- | --- | --- | --- | --- | --- | --- | --- | --- |
|  |  |  |  |  |  |  | <10 | 16-20 | 25-40 | 50-64 | 128 | ≥256 |  |
| *Lynx rufus* | USA (Georgia) | Free ranging | MAT | 6 | 5 (83.3) | 1:25 |  |  |  |  | 5 |  | Dubey et al. 2004 |
| *Lynx rufus* | USA (Pennsylvania) | Free ranging | MAT | 131 | 108 (82.4) | 1:25 |  | 8 |  | 79 |  | 22 | Mucker et al. 2006 |
| *Lynx rufus* | USA (California) | Free ranging | IFA | 3 | 3 (100) | 1:320 |  |  |  |  |  | 3 | Miller et al. 2008 |
| *Lynx rufus* | USA (Colorado) | Free ranging | ELISA | 258 | 111 (43.0) | 1:52 |  |  |  |  |  |  | Bevins et al. 2012 |
| *Lynx rufus* | USA (California) | Free ranging | IFAT | 22 | 16 (72.7) | N.S. |  |  |  |  |  |  | VanWormer et al. 2013 |
| *Lynx rufus* | Mexico (Mexico City) | Captive | MAT | 2 | 2 (100) | 1:25 | 1 |  |  |  |  | 1 | Alvarado-Esquivel et al. 2013 |
| *Lynx rufus* | USA (Minnesota) | Free ranging | MAT | 50 | 29 (58.0) | 1:25 |  | 21 | 8 | 9 | 7 |  | Verma et al. 2016 |
| *Lynx rufus* | Mexico | Captive | ELISA | 2 | 2 (100) | N.S. |  |  |  |  |  |  | Gomez-Rios et al. 2019 |
| **Cheetah** |  |  |  |  |  |  |  |  |  |  |  |  |  |
| *Acinonyx jubatus* | USA (Florida) | Captive | IHA | 16 | 11 (68.7) | 1:64 |  |  |  |  | 6 | 5 | Stover et al. 1990 |
| *Acinonyx jubatus* | Southern Africa | Captive | IFA | 23 | 10 (43.4) | 1:50 |  |  |  | 10 |  |  | Cheadle et al. 1999 |
| *Acinonyx jubatus* | USA (Various areas) | Captive | IFAT | 9 | 7 (77.7) | 1:50 |  |  |  |  | 2 | 5 | Spencer et al. 2003 |
| *Acinonyx jubatus* | Thailand | Captive | LAT | 1 | 1 (100) | 1:64 |  |  |  | 1 |  |  | Thiangtum et al. 2006 |
| *Acinonyx jubatus* | USA (Midwestern zoos) | Captive | MAT | 22 | 6 (27.3) | 1:25 |  |  | 6 |  |  |  | de Camps et al. 2008 |
| *Acinonyx j. soemmerringii* | Qatar (Doha)^¶^ | Captive | MAT | 5 | 5 (100) | 1:25 |  |  |  | 1 |  | 4 | Dubey et al. 2010 |
| *Acinonyx jubatus rex* | Qatar (Doha)^¶^ | Captive | MAT | 1 | 1 (100) | 1:25 |  |  |  |  |  | 1 | Dubey et al. 2010 |
| *Acinonyx j. soemmerringii* | United Arab Emirates○ | Captive | MAT | 34 | 31 (91.1) | 1:25 |  |  |  | 1 |  | 25 | Dubey et al. 2010 |
| **Prionailurus cats** |  |  |  |  |  |  |  |  |  |  |  |  |  |
| *Prionailurus iriomotensis* | Japan | Free ranging | PHA | 16 | 4 (25.0) | 1:256 |  |  |  |  |  |  | Akuzawa et al. 1987 |
| *Prionailurus viverrinus* | Thailand | Captive | LAT | 27 | 6 (22.2) | 1:64 |  |  |  | 6 |  |  | Thiangtum et al. 2006 |
| *Prionailurus bengalensis* | Thailand^*^ | Captive | DT | 2 | 1 (50.0) | 1:8 |  |  |  |  |  |  | Buddhirongawatr et al. 2006 |
| *Prionailurus viverrina* | Thailand^*^ | Captive | DT | 1 | 1 (100) | 1:8 |  |  |  |  |  |  | Buddhirongawatr et al. 2006 |
| *Prionailurus planiceps* | Thailand^*^ | Captive | DT | 2 | 1 (50.0) | 1:8 |  |  |  |  |  |  | Buddhirongawatr et al. 2006 |
| *Prionailurus viverrinus* | USA (Midwestern zoos) | Captive | MAT | 4 | 1 (25.0) | 1:25 |  |  | 1 |  |  |  | de Camps et al. 2008 |
| *Prionailurus bengalensis euptilura* | USA (Midwestern zoos) | Captive | MAT | 1 | 1 (100) | 1:25 |  |  |  |  | 1 |  | de Camps et al. 2008 |
| *Prionailurus viverrinus* | Brazil (São Paulo) | Captive^†^ | IFAT | 1 | 1 (100) | 1:40 |  |  |  |  |  |  | Andre et al. 2010 |
| *Prionailurus viverrinus* | Europe | N.S. | IFAT | 40 | 22 (55.0) | N.S. |  |  |  |  |  |  | Lucht et al. 2019 |
| *Prionailurus bengalensis euptilura* | Russia (Southern Primorye) | Free ranging | ELISA | 23 | 3 (13.0) | N.S. |  |  |  |  |  |  | Naidenko et al. 2019 |
| **Iberian lynx** |  |  |  |  |  |  |  |  |  |  |  |  |  |
| *Lynx pardinus* | Spain | Free ranging | MAT | 27 | 22 (81.5) | 1:25 |  | 1 |  | 3 | 8 | 10 | Sobrino et al. 2007 |
| *Lynx pardinus* | Spain | Free ranging | IHA | 57 | 25 (43.8) | 1:64 |  |  |  | 21 |  |  | Roelke et al. 2008 |
| *Lynx pardinus* | Spain | N.S. | MAT | 26 | 21 (80.7) | 1:25 |  |  |  |  |  |  | Millan et al. 2009 |
| *Lynx pardinus* | Spain | N.S. | MAT | 129 | 81 (62.8) | 1:25 |  |  |  | 7 | 31 | 91 | Garcia-Bocanegra et al. 2009 |

**Table 4.** Continued

| **Species** | **Location** | **Status** | **Test** | **Sample size** | **Positive (%)** | **Cut-off titer** | **No. with titers of** | | | | | | **References** |
| --- | --- | --- | --- | --- | --- | --- | --- | --- | --- | --- | --- | --- | --- |
|  |  |  |  |  |  |  | <10 | 16-20 | 25-40 | 50-64 | 128 | ≥256 |  |
| **Pampas cat** |  |  |  |  |  |  |  |  |  |  |  |  |  |
| *Leopardus colocolo* | Brazil | Captive | MAT | 8 | 1 (12.0) | ≥1:20 |  |  |  |  |  |  | Silva et al. 2007 |
| *Leopardus colocolo* | Brazil | Captive | MAT | 8 | 1 (12.5) | 1:20 |  |  |  |  |  | 1 | Silva et al. 2001b |
| *Leopardus colocolo* | Brazil (São Paulo) | Captive^†^ | IFAT | 3 | 1 (33.3) | 1:40 |  |  |  |  |  |  | Andre et al. 2010 |
| **Serval** |  |  |  |  |  |  |  |  |  |  |  |  |  |
| *Leptailurus serval* | USA (Florida) | Captive | ELISA | 2 | 1 (50.0) | 1:64 |  |  |  |  |  |  | Lappin et al. 1991 |
| *Leptailurus serval* | Brazil | Captive | MAT | 2 | 2 (100) | 1:20 |  |  |  | 2 |  |  | Silva et al. 2001a |
| *Leptailurus serval* | USA (Various areas) | Captive | IFAT | 3 | 1 (33.3) | 1:50 |  |  |  |  | 1 |  | Spencer et al. 2003 |
| *Leptailurus serval* | Brazil (São Paulo) | Captive^†^ | IFAT | 1 | 1 (100) | 1:40 |  |  |  |  |  |  | Andre et al. 2010 |
| **Pallas's cat** |  |  |  |  |  |  |  |  |  |  |  |  |  |
| *Otocolobus manul* | USA (Wisconsin) | Captive | MAT | 3 | 2 (67.0) | 1:64 |  |  |  |  |  |  | Dubey et al. 1988 |
| *Otocolobus manul* | USA (Ohio) | Captive | ELISA | 14 | 11 (78.6) | N.S. |  |  | 13 |  |  |  | Swanson 1999 |
| *Otocolobus manul* | USA (Colorado) | Captive | LAT | 3 | 3 (100) | 1:32 |  |  |  | 15 |  |  | Kenny et al. 2002 |
| *Otocolobus manul* | USA (Oklahoma) | Captive | KELA | 6 | 6 (100) | 1:48 |  |  |  |  |  | 6 | Ketz-Riley et al. 2003 |
| *Otocolobus manul* | Austria | Captive | MAT | 8 | 8 (100) | 1:40 |  |  |  |  |  | 8 | Basso et al. 2005 |
| *Otocolobus manul* | Mongolia | Free ranging | ELISA | 15 | 2 (13.3) | 1:64 |  |  |  |  |  | 2 | Brown et al. 2005 |
| *Otocolobus manul* | USA (North American zoos) | Captive | ELISA | 9 | 9 (100) | 1:64 |  |  |  |  |  | 9 | Brown et al. 2005 |
| *Otocolobus manul* | USA (Midwestern zoos) | Captive | MAT | 5 | 1 (20.0) | 1:25 |  |  |  |  |  |  | de Camps et al. 2008 |
| *Otocolobus manul* | Russia (Daurian Steppe) | Free ranging | EIA | 16 | 2 (12.5) | N.S. |  |  |  |  |  |  | Naidenko et al. 2014 |
| *Otocolobus manul* | Europe | N.S. | IFA | 52 | 47 (90.4) | N.S. |  |  |  |  |  |  | Lucht et al. 2019 |
| **Jungle cat** |  |  |  |  |  |  |  |  |  |  |  |  |  |
| *Felis chaus* | Brazil | Captive | MAT | 2 | 2 (100) | 1:20 |  |  |  | 2 |  |  | Silva et al. 2001a |
| *Felis chaus* | Thailand^*^ | Captive | DT | 4 | 0 (0.0) | N.S. |  |  |  |  |  |  | Buddhirongawatr et al. 2006 |
| **European wildcat** |  |  |  |  |  |  |  |  |  |  |  |  |  |
| *Felis silvestris* | United Kingdom | Free ranging | IHA | 45 | 28 (62.0) | 1:16 |  |  |  |  |  |  | Yamaguchi et al. 1996 |
| *Felis silvestris* | Spain | Free ranging | MAT | 6 | 3 (50.0) | 1:25 |  |  |  |  | 1 | 2 | Sobrino et al. 2007 |
| *Felis sylvestris gordoni* | United Arab Emirates | Captive | MAT | 36 | 31 (86.1) | 1:25 |  |  |  | 2 | 5 | 17 | Pas and Dubey 2008a |
| *Felis sylvestris gordoni* | Qatar (Doha)^¶^ | Captive | MAT | 1 | 1 (100) | 1:25 |  |  |  |  |  | 1 | Dubey et al. 2010 |
| *Felis sylvestris gordoni* | United Arab Emirates ○ | Captive | MAT | 5 | 5 (100) | 1:25 |  |  |  |  |  | 3 | Dubey et al. 2010 |
| *Felis silvestris* | Romania (Timişoara Zoo) | Captive | ELISA | 2 | 2 (100) | N.S. |  |  |  |  |  |  | Darabus et al. 2011 |
| *Felis silvestris* | Portugal (Southern) | Free ranging | MAT | 6 | 5 (83.3) | 1:20 |  |  |  |  |  |  | Waap et al. 2016 |
| **Sand cat** |  |  |  |  |  |  |  |  |  |  |  |  |  |
| *Felis margarita* | United Arab Emirates | Captive | MAT | 6 | 6 (100) | 1:25 |  |  |  |  | 1 | 5 | Pas and Dubey 2008b |

**Table 4.** Continued

| **Species** | **Location** | **Status** | **Test** | **Sample size** | **Positive (%)** | **Cut-off titer** | **No. with titers of** | | | | | | **References** |
| --- | --- | --- | --- | --- | --- | --- | --- | --- | --- | --- | --- | --- | --- |
|  |  |  |  |  |  |  | <10 | 16-20 | 25-40 | 50-64 | 128 | ≥256 |  |
| *Felis margarita* | United Arab Emirates | Captive | LAT | 4 | 3 (75.0) | 1:64 |  |  |  |  |  | 3 | Pas and Dubey 2008b |
| *Felis margarita harrisoni* | Qatar (Doha)^¶^ | Captive | MAT | 20 | 14 (70.0) | 1:25 |  |  |  |  |  | 13 | Dubey et al. 2010 |
| *Felis margarita* | Europe | N.S. | IFAT | 87 | 47 (54.0) | N.S. |  |  |  |  |  |  | Lucht et al. 2019 |
| **Asian golden cat** |  |  |  |  |  |  |  |  |  |  |  |  |  |
| *Catopuma temmincki* | China (Shanghai) | Captive | MAT | 6 | 5 (83.3) | 1:20 |  |  | 2 |  |  | 3 | Zhang et al. 2000 |
| *Catopuma temmincki* | Thailand | Captive | LAT | 8 | 1 (12.5) | 1:64 |  |  |  |  |  |  | Thiangtum et al. 2006 |
| *Catopuma temminckii* | Europe | N.S. | IFA | 2 | 1 (50.0) | N.S. |  |  |  |  |  |  | Lucht et al. 2019 |
| **Canadian lynx** |  |  |  |  |  |  |  |  |  |  |  |  |  |
| *Lynx canadiensis* | Canada (Quebec) | Free ranging | MAT | 106 | 47 (44.3) | 1:25 |  |  |  | 41 |  |  | Labelle et al. 2001 |
| *Lynx canadiensis* | USA (California) | Captive | IFA | 1 | 1 (100) | 1:50 |  |  |  | 1 |  |  | Spencer et al. 2003 |
| *Lynx canadiensis* | Canada (Quebec) | Free ranging | MAT | 84 | 12 (14.0) | 1:50 |  |  |  |  |  |  | Simon et al. 2013 |
| **Clouded leopard** |  |  |  |  |  |  |  |  |  |  |  |  |  |
| *Neofelis nebulosa* | USA (California) | Captive | IFA | 2 | 1 (50.0) | 1:50 |  |  |  | 1 |  |  | Spencer et al. 2003 |
| *Neofelis nebulosa* | Thailand | Captive | LAT | 16 | 2 (12.5) | 1:64 |  |  |  |  | 2 |  | Thiangtum et al. 2006 |
| *Neofelis nebulosa* | Thailand^*^ | Captive | DT | 2 | 2 (100) | 1:4 |  |  |  |  |  |  | Buddhirongawatr et al. 2006 |
| *Neofelis nebulosa* | USA (Midwestern zoos) | Captive | MAT | 7 | 1 (14.3) | 1:25 |  |  | 1 |  |  |  | de Camps et al. 2008 |
| **Masked palm civet** |  |  |  |  |  |  |  |  |  |  |  |  |  |
| *Paguma larvata* | China (Henan) | Farm | MAT | 10 | 0 (0.0) | 1:25 |  |  |  |  |  |  | Yang et al. 2017 |
| **Common genet** |  |  |  |  |  |  |  |  |  |  |  |  |  |
| *Genetta genetta* | Brazil (Sao Paulo) | Captive^†^ | IFAT | 1 | 1 (100) | 1:40 |  |  |  |  |  |  | Andre et al. 2010 |

^a^ Felid scientific names listed according to International Union for Conservation of Nature (IUCN) classifications; ^*^ Khao Kheow Open Zoo and Khao Pratab Chang Breeding Center; N.S., Not specified; PHA, passive hemagglutination assay; ^†^ Brazilian zoos of São Paulo, Mato Grosso states and Federal District; ^¶^ Wabra Wildlife Preservation

○ Breeding Centre for Endangered Arabian Wildlife; KELA, kinetic enzyme-linked immunosorbent assay; EIA, enzyme immunoassay; MB, Mouse bioassay

**References**

1. Lappin, M.R., Jacobson, E.R., Kollias, G.V., Powell, C.C., Stover, J., 1991. Comparison of serologic assays for the diagnosis of toxoplasmosis in nondomestic felids. Journal of Zoo and Wildlife Medicine 22, 169–174.
2. Spencer, J.A., Morkel, P., 1993. Serological survey of sera from lions in Etosha National Park. South African Journal of Wildlife Research 23, 60–61.
3. Cheadle, M.A., Spencer, J.A., Blackburn, B.L., 1999. Seroprevalences of Neospora caninum and *Toxoplasma gondii* in nondomestic felids from southern Africa. Journal of Zoo and Wildlife Medicine 30, 248–251.
4. Silva, J.C.R., Ogassawara, S., Marvulo, M.F.V., Ferreira-Neto, J.S., Dubey, J.P., 2001a. Toxoplasma gondii antibodies in exotic wild felids from Brazilian zoos. Journal of Zoo and Wildlife Medicine 32, 349–351. Silva, J.C.R., Ogassawara, S., Adania, C.H., Ferreira, F., Gennari, S.M., Dubey, J.P., Ferreira-Neto, J.S., 2001b. Seroprevalence of Toxoplasma gondii in captive neotropical felids from Brazil. Veterinary Parasitology 102, 217–224.
5. Penzhorn, B.L., Stylianides, E., van Vuuren, M., Alexander, K., Meltzer, D.G.A., Mukarati, N., 2002. Seroprevalence of Toxoplasma gondii in free-ranging lion and leopard populations in southern Africa. South African Journal of Wildlife Research 32, 163–165.
6. Spencer, J.A., Higginbotham, M.J., Blagburn, B.L., 2003. Seroprevalence of Neospora caninum and Toxoplasma gondii in captive and free-ranging nondomestic felids in the United States. Journal of Zoo and Wildlife Medicine 34, 246–249.
7. Hove, T., Mukaratirwa, S., 2005. Seroprevalence of *Toxoplasma gondii* in farm-reared ostriches and wild game species from Zimbabwe. Acta Tropica 94, 49–53.
8. Thiangtum, K., Nimsuphun, B., Pinyopanuwat, N., Chimnoi, W., Tunwattana, W., Tongthainan, D., Jittapalapong, S., Rukkwamsuk, T., Maruyama, S., 2006. Seroprevalence of Toxoplasma gondii in captive felids in Thailand. Veterinary Parasitology 136, 351–355.
9. Rivetti Junior AV, Caxito FA, Resende M, Lobato ZI. Avaliaçao sorologica para *Toxoplasma gondii* pela imunofluorescência indireta e detecçto do virus da imunodeficiência felina pela nested PCR em felinos selvagens. Arq Bras Med Vet Zootec. 2008;60:1281–3.
10. de Camps S, Dubey JP, Saville WJ. Seroepidemiology of *Toxoplasma gondii* in zoo animals in selected zoos in the midwestern United States. J Parasitol. 2008;94:648–53.
11. Andre MR, Adania CH, Teixeira RH, Silva KF, Jusi MM, Machado ST, et al. Antibodies to *Toxoplasma gondii* and *Neospora caninum* in captive neotropical and exotic wild canids and felids. J Parasitol. 2010;96:1007–9
12. Alvarado-Esquivel C, Gayosso-Dominguez EA, Villena I, Dubey JP. Seroprevalence of *Toxoplasma gondii* infection in captive mammals in three zoos in Mexico City, Mexico. J Zoo Wildl Med. 2013;44:803–6.
13. Silva JCR, Marvulo MF, Dias RA, Ferreira F, Amaku M, Adania CH, et al. Risk factors associated with sero-positivity to *Toxoplasma gondii* in captive neotropical felids from Brazil. Prev Vet Med. 2007;78:286–95.
14. Demar, M., Ajzenberg, D., Serrurier, B., Darde, M.L., Carme, B., 2008. Case report: atypical *Toxoplasma* *gondii* strain from a free-living jaguar (Panthera onca) in French Guiana. American Journal of Tropical Medicine and Hygiene 78, 195–197.
15. Gomez-Rios A, Ortega-Pacheco A, Gutierrez-Blanco E, Acosta-Viana KY, Guzman-Marin E, Guiris-Andrade MD, Hernandez-Cortazar IB, Lopez-Alonso R, Cruz-Alda E, Jimenez-Coello M. *Toxoplasma gondii* in Captive Wild Felids of Mexico: Its Frequency and Capability to Eliminate Oocysts. Vector Borne and zoonotic diseases. 2019……………………..
16. Fiorello, C.V., Robbins, R.G., Maffei, L., Wade, S.E., 2006. Parasites of free-ranging small canids and felids in the Bolivian Chaco. Journal of Zoo and Wildlife Medicine 37, 130–134.
17. Ullmann LS, da Silva RC, de Moraes W, Cubas ZS, dos Santos LC, Hoffmann JL, et al. Serological survey of *Toxoplasma gondii* in captive neotropical felids from southern Brazil. Vet Parasitol. 2010;172:144-6.
18. Lücht M, Stagegaard J, Conraths FJ, Schares G. *Toxoplasma gondii* in small exotic felids from zoos in Europe and the Middle East: serological prevalence and risk factors. 2019; 12:449.
19. Sogorb F, Jamra LF, Guimaraes EC. Toxoplasmosis in animals of Sao Paulo, Brazil. Rev Inst Med Trop Sao Paulo. 1977;13:191-4.
20. Roelke, M.E., Forrester, D.J., Jacobson, E.R., Kollias, G.V., Scott, F.W., Barr, M.C., Evermann, J.F., Pirtle, E.C., 1993. Seroprevalence of infectious-disease agents in free-ranging Florida panthers (*Felis concolor coryi*). Journal of Wildlife Diseases 29, 36-49.
21. Paul-Murphy, J., Work, T., Hunter, D., McFie, E., Fjelline, D., 1994. Serologic survey and serum biochemical reference ranges of the free-ranging mountain lion (*Felis concolor*) in California. Journal of Wildlife Diseases 30, 205-215.
22. Stephen, C., Haines, D., Bollinger, T., Atkinson, K., Schwantje, H., 1996. Serological evidence of *Toxoplasma* infection in cougars on Vancouver Island, British Columbia. Canadian Veterinary Journal 37, 241.
23. Aramini, J.J., Stephen, C., Dubey, J.P., 1998. *Toxoplasma gondii* in Vancouver Island cougars (*Felis concolor vancouverensis*): serology and oocyst shedding. Journal of Parasitology 84, 438-440.
24. Zhang SY, Wei MX, Zhou ZY, Yu JY, Shi XQ. Prevalence of antibodies to *Toxoplasma gondii* in the sera of rare wildlife in the Shanghai Zoological Garden, People’s Republic of China. Parasitol Int. 2000;49:171-4.
25. Kikuchi, Y., Chomel, B.B., Kasten, R.W., Martenson, J.S., Swift, P.K., O’Brien, S.J., 2004. Seroprevalence of *Toxoplasma gondii* in American free-ranging or captive pumas (*Felis concolor*) and bobcats (*Lynx rufus*). Veterinary Parasitology 120, 1-9.
26. Buddhirongawatr R, Tungsudjai S, Chaichoune K, Sangloung C, Tantawiwattananon N, Phonaknguen R, et al. Detection of *Toxolasma* *gondii* in captive wild felids. Southeast Asian J Trop Med Public Health. 2006;37(Suppl. 3):15–7.
27. Miller, M.A., Miller, W.A., Conrad, P.A., James, E.R., Melli, A.C., Leutenegger, C.M., Dabritz, H.A., Packham, A.E., Paradies, D., Harris, M., Ames, J., Jessup, D.A., Worcester, K., Grigg, M.E., 2008. Type X *Toxoplasma gondii* in a wild mussel and terrestrial carnivores from coastal California: new linkages between terrestrial mammals, runoff and toxoplasmosis of sea otters. International Journal for Parasitology 38, 1319–1328.
28. Bevins SN, Carver S, Boydston EE, Lyren LM, Alldredge M, Logan KA, Riley SPD, Fisher RN, Vickers TW, Boyce W, Salman M, Lappin MR, Crooks KR, VandeWoude S. Three Pathogens in Sympatric Populations of Pumas, Bobcats, and Domestic Cats: Implications for Infectious Disease Transmission. PLoS ONE 2012;7(2): e31403
29. Brown, AS, Lappin MR, Brown JL, Munkhtsog B, Swanson WF. Exploring the ecologic basis for extreme susceptibility of Pallas cats (*Otocolobus manul*) to fatal toxoplasmosis. Journal of Wildlife Diseases, 2005; 41(4):691-700
30. Sobrino, R., Cabezon, O., Millan, J., Pabon, M., Arnal, M.C., Luco, D.F., Gortazar, C., Dubey, J.P., Almeria, S., 2007. Seroprevalence of *Toxoplasma* *gondii* antibodies in wild carnivores from Spain. Veterinary Parasitology 148, 187–192.
31. Dubey JP, Pas A, Rajendran C, Kwok OCH, Ferreira LR, Martins J, Hebel C, Hammer S, Su C. Toxoplasmosis in Sand cats (Felis margarita) and other animals in the Breeding Centre for Endangered Arabian Wildlife in the United Arab Emirates and Al Wabra Wildlife Preservation, the State of Qatar. Veterinary Parasitology 2010;172:195-203.
32. Luo H, Li K, Zhang H, Gan P, Shahzad M, Wu X, Lan Y, Wang J. Seroprevalence of Toxoplasma gondii infection in zoo and domestic animals in Jiangxi Province, China. Parasite 2017, 24, 7
33. Yang YR, Feng YJ, Lu YY, Dong H, Li TY, Jiang YB, Zhu XQ, Zhang LX. Antibody Detection, Isolation, Genotyping, and Virulence of *Toxoplasma gondii* in Captive Felids from China. Front. Microbiol. 20178:1414.
34. Rendon-Franco E, Caso-Aguilar A, Jimenez-Sanchez NJ, Hernandez-Jauregui DMB, Sandoval-Sanchez AL, Zepeda-Lopez HM. Prevalence of Anti-*Toxoplasma gondii* Antibody in Free-ranging Ocelots (*Leopardus pardalis*) from Tamaulipas, Mexico. Journal of Wildlife Diseases, 2012;48(3):829-831.
35. Philippa, J.D.W., Leighton, F.A., Daoust, P.Y., Nielsen, O., Pagliarulo, M., Schwantje, H., Shury, T., van Herwijnen, R., Martina, B.E.E., Kuiken, T., van de Bildt, M.W.G., Osterhaus, A.D.M.E., 2004. Antibodies to selected pathogens in free-ranging terrestrial carnivores and marine mammals in Canada. Veterinary Record 155, 135–140.
36. Dorny, P., Fransen, A.J., 1989. Toxoplasmosis in a Siberian tiger (Panthera tigris altaica). Veterinary Record 125, 647.
37. Vitaliano SN, Soares HS, Minervino AHH, Santos ALQ, Werther K, Marvulo MFV, Siqueira DB, Pena HFJ, Soares RM, Su C, Gennari SM. Genetic characterization of *Toxoplasma gondii* from Brazilian wildlife revealed abundant new genotypes. International Journal for Parasitology: Parasites and Wildlife 2014;3:276-283
38. Labelle, P., Dubey, J.P., Mikaelian, I., Blanchette, N., Lafond, R., St-Onge, S., Martineau, D., 2001. Seroprevalence of antibodies to *Toxoplasma* *gondii* in lynx (*Lynx canadensis*) and bobcats (Lynx rufus) from Quebec, Canada. Journal of Parasitology 87, 1194-1196.
39. Akuzawa, M., Mochizuki, M., Yasuda, N., 1987. Hematological and parasitological study of the Iriomote cat (*Prionailurus iriomotensis*). Canadian Journal of Zoology 65, 946–949.
40. Zarnke R.L, Dubey JP, Ver Hoef JM, McNay ME, Kwok OCH. Serologic survey for *Toxoplasma gondii* in lynx (*Felis lynx*) from interior Alaska. Journal of Wildlife Diseases 2001;37:36-38.
41. Roelke, M.E., Johnson, W.E., Millan, J., Palomares, F., Revilla, E., Rodriquez, A., Calzada, J., Ferreras, P., Leon-Vizcaino, L., Delibes, M., O’Brien, S.J., 2008. Exposure to disease agents in the endangered Iberian lynx (*Lynx pardinus*). European Journal of Wildlife Research 54, 171–178.
42. Ryser-Degiorgis, M.P., Jakubek, E.B., af Segerstad, C.H., Brojer, C., Morner, T., Jansson, D.S., Lunden, A., Uggla, A., 2006. Serological survey of *Toxoplasma gondii* infection in free-ranging Eurasian lynx (*Lynx lynx*) from Sweden. Journal of Wildlife Diseases 42, 182–187.
43. Marchiondo, A.A., Duszynski, D.W., Maupin, G.O., 1976. Prevalence of antibodies to *Toxoplasma gondii* in wild and domestic animals of New Mexico, Arizona and Colorado. Journal of Wildlife Diseases 12, 226–232.
44. Smith, D.D., Frenkel, J.K., 1995. Prevalence of antibodies to Toxoplasma gondii in wild mammals of Missouri and east central Kansas: biologic and ecologic considerations of transmission. Journal of Wildlife Diseases 31, 15–21.
45. Riley, S.P.D., Foley, J., Chomel, B., 2004. Exposure to feline and canine pathogens in bobcats and gray foxes in urban and rural zones of a national park in California. Journal of Wildlife Diseases 40, 11–22.
46. Dubey, J.P., Graham, D.H., de Young, R.W., Dahl, E., Eberhard, M.L., Nace, E.K., Won, K., Bishop, H., Punkosdy, G., Sreekumar, C., Vianna, M.C.B., Shen, S.K., Kwok, O.C.H., Sumners, J.A., Demarais, S., Humphreys, J.G., Lehmann, T., 2004a. Molecular and biologic characteristics of *Toxoplasma gondii* isolates from wildlife in the United States. Journal of Parasitology 90, 67–71.
47. Mucker, E.M., Dubey, J.P., Lovallo, M.J., Humphreys, J.G., 2006. Seroprevalence of antibodies to *Toxoplasma gondii* in the Pennsylvania bobcat (*Lynx rufus rufus*). Journal of Wildlife Diseases 42, 188–191.
48. Stover, J., Jacobson, E.R., Lukas, J., Lappin, M.R., Buergelt, C.D., 1990. *Toxoplasma gondii* in a collection of nondomestic ruminants. Journal of Zoo and Wildlife Medicine 21, 295–301.
49. Millan, J., Candela, M.G., Palomares, F., Cubero, M.J., Rodriguez, A., Barral, M., de la Fuente, J., Almeria, S., Leon-Vizcaino, L., 2009. Disease threats to the endangered Ilerian lynx (*Lynx pardinus*). Veterinary Journal 182, 114–124.
50. Garcia-Bocanegra, I., J. P. Dubey, F. Martinez et al., 2009. Factors affecting seroprevalence of *Toxoplasma gondii* in the endangered Iberian *lynx* (*Lynx pardinus*). *Vet. Parasitol.* ……………………………
51. Dubey, J.P., Gendron-Fitzpatrick, A.P., Lenhard, A.L., Bowman, D., 1988. Fatal toxoplasmosis and enteroepithelial stages of *Toxoplasma gondii* in a Pallas cat (*Felis manul*). Journal of Protozoology 35, 528–530.
52. Swanson, W.F., 1999. Toxoplasmosis and neonatal mortality in Pallas’ cats: a survey of North American zoological institutions. Proceedings American Association of Zoo Veterinarians 1999, 347–350.
53. Kenny, D.E., Lappin, M.R., Knightly, F., Baier, J., Brewer, M., Getzy, D.M., 2002. Toxoplasmosis in Pallas’ cats (*Otocolobus felis manul*) at the Denver Zoological Gardens. Journal of Zoo and Wildlife Medicine 33, 131–138.
54. Ketz-Riley, C.J., Ritchey, J.W., Hoover, J.P., Johnson, C.M., Barrie, M.T., 2003. Immunodeficiency associated with multiple concurrent infections in captive Pallas’ cats (*Otocolobus manul*). Journal of Zoo and Wildlife Medicine 34, 239– 245.
55. Basso, W., Edelhofer, R., Zenker, W., Mostl, K., Kubber-Heiss, A., Prosl, H., 2005. Toxoplasmosis in Pallas’ cats (*Otocolobus manul*) raised in captivity. Parasitology 130, 293–299.
56. Naidenko SV, Pavlova EV, Kirilyuk VE. Detection of seasonal weight loss and a serologic survey of potential pathogens in wild Pallasʼ cats (*Felis* [*Otocolobus*] *manul*) of the Daurian Steppe, Russia. J Wildl Dis. 2014;50:188-94.
57. Yamaguchi, N., MacDonald, D.W., Passanisi, W.C., Harbour, D.A., Hopper, C.D., 1996. Parasite prevalence in free-ranging farm cats, *Felis silvestris catus*. Epidemiologic Infection 116, 217-223.
58. Pas, A., Dubey, J.P., 2008a. Seroprevalence of antibodies to *Toxoplasma gondii* in Gordon’s wild cat (*Felis silvestris gordoni*) in the Middle East. Journal of Parasitology 94, 1169.
59. Pas, A., Dubey, J.P., 2008. Fatal toxoplasmosis in sand cats (*Felis margarita*). Journal of Zoo Wildlife Medicine 39, 362-369.
60. Vanwormer E, Conrad PA, Miller MA, Melli AC, Carpenter TE, Mazet JA*. Toxoplasma gondii*, source to sea: higher contribution of domestic felids to terrestrial parasite loading despite lower infection prevalence. Ecohealth. 2013;10(3):277-89.
61. Verma SK, Minicucci L, Murphy D, Carstensen M, Humpal C, Wolf P, Calero-Bernal R, Cerqueira-Cézar CK, Kwok OC, Su C, Hill D, Dubey JP. Antibody Detection and Molecular Characterization of *Toxoplasma gondii* from Bobcats (*Lynx rufus*), Domestic Cats (*Felis catus*), and Wildlife from Minnesota, USA. J Eukaryot Microbiol. 2016;63(5):567-71
62. Simon A, Poulin MB, Rousseau AN, Dubey JP, Ogden NH. [Spatiotemporal dynamics of *Toxoplasma gondii* infection in Canadian lynx (*Lynx canadensis*) in western Quebec, Canada.](http://ovidsp.tx.ovid.com/sp-3.31.1b/ovidweb.cgi?&S=IIPAFPDDHLDDEIPFNCEKOBGCCILLAA00&Complete+Reference=S.sh.69|120|1)  Journal of Wildlife Diseases 2013;49(1):39-48
63. Oertley KD, Walls KW. [Prevalence of antibodies to *Toxoplasma gondii* among bobcats of West Virginia and Georgia.](http://ovidsp.tx.ovid.com/sp-3.31.1b/ovidweb.cgi?&S=IIPAFPDDHLDDEIPFNCEKOBGCCILLAA00&Complete+Reference=S.sh.69|224|1) Journal of the American Veterinary Medical Association 1980;177(9):852-853
64. Franti CE, Riemann HP, Behymer DE, Suther D, Howarth JA, Ruppanner R. Prevalence of *Toxoplasma gondii* antibodies in wild and domestic animals in northern California. J Am Vet Med Assoc. 1976;169(9):901-6
65. Naidenko SV, Hernandez-Blanco JA, Erofeeva MN, Litvinov MN, Rozhnov VV. Serum prevalence to non-viral pathogens in wild felids of Southern Primorye, Russia. Nature Conservation Research 2019; 4(1)……………………..
66. Waap H, Nunes T, Vaz Y, Leitao A. [Serological survey of *Toxoplasma gondii* and *Besnoitia besnoiti* in a wildlife conservation area in southern Portugal.](http://ovidsp.tx.ovid.com/sp-3.31.1b/ovidweb.cgi?&S=IIPAFPDDHLDDEIPFNCEKOBGCCILLAA00&Complete+Reference=S.sh.69|580|1)  Veterinary Parasitology: Regional Studies and Reports 2016;3/4:7-12.
67. Darabus G, Afrenie M, Olariu RT, Ilie MS, Balint A, Hotea I. Epidemiological remarks on *Toxoplasma gondii* infection in Timişoara Zoo. 2011;Sci Parasitol 12(1):33-37
68. Ferreira SCM, Torelli F, Klein S, Fyumagwa R, Karesh WB, Hofer H, Seeber F, East M. Evidence of high exposure to *Toxoplasma gondii* in free-ranging and captive African carnivores. Int J Parasitol Parasites Wildl. 2019; 8:111-117
69. Marková J., Machačová T., Bártová E., Sedlák K., Budíková M., Silvestre P., Laricchiuta P., Russo M., Veneziano V. Toxoplasma gondii, Neospora caninum and Encephalitozoon cuniculi in animals from captivity (zoo and circus animals) J Eukaryot Microbiol. 2019;66(3):442-446
