# Supplementary material for: Toxoplasma gondii infection in domestic and wild felids as public health concerns: a systematic review and meta-analysis
Source: Sci Rep. 2021 May 4;11:9509. doi: 10.1038/s41598-021-89031-8 (PMC8097069; doi:10.1038/s41598-021-89031-8)
Supplement: Supplementary file 6 — Supplementary Information 6. [file 41598_2021_89031_MOESM6_ESM.doc]

**Table S4.** Characteristics of the eligible studies based on detection of *T. gondii*-like oocyst and *T. gondii* oocyst DNA in domestic cat feces (sorted by continent and publication date)

| **Location** | **Period** | **Stray/Pet** | **Age groups** | **Sample size** | **Positive (%)** | **Method** | **Symp.** | **Author and publication date** |
| --- | --- | --- | --- | --- | --- | --- | --- | --- |
| **AFRICA** |  |  |  |  |  |  |  |  |
| Nigeria (Niger Delta) | 1983 | Pet^¶^ | N.S. | 200 | 44 (22.0) | Microscopy | S | Arene 1984 |
| Nigeria | 1989 | N.S. | N.S. | 52 | 0 (0.0) | Microscopy | N.S. | Umeche 1990 |
| Egypt (Giza) | 2008-2009 | Stray | N.S. | 154 | 1 (0.65) | Mouse bioassay | N.S. | Al Kappany et al. 2010 |
| Ethiopia (Addis Ababa) | 2011 | Stray | 2 m - 8 y | 36 | 7 (19.4) | Mouse bioassay | N.S. | Dubey et al. 2013 |
| Kenya (Thika) | 2015 | Pet | ≥1 y | 103 | 8 (7.8) | PCR | N.S. | Njuguna et al. 2017 |
| Mauritania (Nouakchott) | 2015 | Stray & pet | ≤2 y; >2 y | 100 | 23 (23.0) | Microscopy | N.S. | Salem et al. 2017 |
| **ASIA** |  |  |  |  |  |  |  |  |
| Japan (Kanto) | 1971 | Stray | N.S. | 90 | 1 (1.1) | Mouse bioassay | N.S. | Werner and Walton 1973 |
| Japan (Western area) | 1988-1990 | Stray | N.S. | 335 | 1 (0.3) | Microscopy | A | Oikawa et al. 1990e |
| Taiwan | 1989 | Pet¶ | N.S. | 96 | 0 (0.0) | Microscopy | S | Lin et al. 1990 |
| India (Maharashtra) | 1991 | Stray | N.S. | 9 | 1 (11.0) | Microscopy | N.S. | Shastri and Ratnaparkhi 1992 |
| Singapore | 1991 | Stray | N.S. | 722 | 0 (0.0) | Microscopy | N.S. | Chong et al. 1993 |
| Bangladesh (Mymensingh) | 1995 | Stray | N.S. | 24 | 1 (4.16) | Microscopy | A | Samad et al. 1997 |
| Iran (Khorasan) | 1996-1997 | Stray | N.S. | 82 | 1 (1.2) | Micro & bioassay | N.S. | Razmi 1999 |
| Japan (Chiba) | 1998-1999 | Unwanted^b^ | N.S. | 326 | 1 (0.3) | Microscopy | A | Hata et al. 2000 |
| Turkey (Van) | 2002 | Pet | N.S. | 62 | 0 (0.0) | Microscopy | N.S. | Tutuncu et al. 2003 |
| China (Guangzhou) | 2006 | Pet^f^ | N.S. | 26 | 0 (0.0) | Mouse bioassay | A | Dubey et al. 2007 |
| Iran (Kashan) | 2004-2005 | Stray | N.S. | 50 | 0 (0.0) | Microscopy | A | Hooshyar et al. 2007 |
| Israel (Jerusalem) | 2006 | Stray | 4 w; <6 y | 122 | 11 (9.0) | PCR | A | Salant et al. 2007 |
| Turkey (Nigde) | 2003 | Stray | N.S. | 72 | 0 (0.0) | Microscopy | A | Karatepe et al. 2008 |
| Iran (Sari) | 2004 | Stray | N.S. | 100 | 0 (0.0) | Microscopy | A | Sharif et al. 2009 |
| Iran (Zanjan) | 2007-2008 | Stray | N.S. | 100 | 42 (42.0) | Microscopy | A | Esmaeilzadehet al. 2009 |
| Iran (Urmia) | 2008-2010 | Stray & pet | N.S. | 130 | 3 (2.3) | Microscopy | A & S | Raeghi et al. 2011 |
| Iran (Ahvaz) | 2006-2009 | Pet | <6m; 6m-3y; >3y | 198 | 0 (0.0) | Microscopy | N.S. | Mosallanejad et al. 2011 |
| China (Beijing) | 2009-2010 | Stray | N.S. | 23 | 0 (0.0) | Microscopy | A | Qian et al. 2012 |
| Kuwait | 2012 | Stray | <6; ≥6m | 240 | 5 (2.1) | Microscopy | N.S. | Abdou et al. 2013e |
| India (Jammu) | 2009-2010 | Stray | N.S. | 100 | 88 (88.0) | Microscopy | N.S. | Borkataki et al. 2013 |
| Iran (Ahvaz) | 2012 | Stray | N.S. | 141 | 2 (1.4) | Microscopy | N.S. | Khademvatan et al. 2014 |
| Qatar (Doha) | 2008-2010 | Stray | N.S. | 4,652 | 424 (9.1) | Microscopy | N.S. | Abu-Madi and Behnke 2014 |
| China (various areas) | 2013-2014 | Stray & pet | ≤12 m; >12 m | 360 | 11 (3.06) | Microscopy | N.S. | Yang et al. 2015 |
| Korea (Seoul) | 2013 | Stray | <3; >3m | 300 | 14 (4.7) | Nested-PCR | N.S. | Jung et al. 2015 |

**Table 1.** Continued

| **Location** | **Period** | **Stray/Pet** | **Age groups** | **Sample size** | **Positive (%)** | **Method** | **Symp.** | | | | | **Author and publication date** |  |
| --- | --- | --- | --- | --- | --- | --- | --- | --- | --- | --- | --- | --- | --- |
| Iran (Ahvaz) | 2012 | Stray | N.S. | 486 | 35 (7.2) | PCR-RFLP | | N.S. | | | Tavalla et al. 2017 | | |
| Iran (Kerman) | 2011-2012 | Stray | N.S. | 100 | 16 (16.0) | Microscopy | | | N.S. | Beigi et al. 2017 | | |  |
| Korea (nine major cities) | 2016 | Stray & pet | 1-12 y | 150 | 0 (0.0) | PCR | | | N.S. | Kim et al. 2017 | | |  |
| Iran (Shiraz) | 2017 | Stray | N.S. | 29 | 0 (0.0) | Microscopy | | | A & S | Asgari et al. 2018 | | |  |
| Japan (Tokachi) | 2013-2014 | Pet¶ | <1; 1-2; 2-3; >3 y | 351 | 1 (0.28) | Microscopy | | | S | Salman et al. 2018 | | |  |
| Pakistan (Lahore) | 2013-2014 | Pet | 1-6 m; >6 m | 417 | 11 (2.3) | PCR | | | N.S. | Nabi et al. 2018 | | |  |
| Iran (Khorasan Razavi) | 2016 | Stray | <6m; 6m-3y; >3y | 156 | 4 (2.56) | Microscopy | | | N.S. | Khodaverdi and Razmi 2019 | | |  |
| Saudi Arabia (Riyadh) | 2017 | Stray & pet | N.S. | 200 | 24 (12.0) | Microscopy | | | N.S. | Mohammed et al. 2019 | | |  |
| **AUSTRALIA** |  |  |  |  |  |  | | |  |  | | |  |
| New Zealand (North Island) | 1974-1976 | Stray | Mixed ages | 508 | 5 (98.0) | Microscopy | | | N.S. | McKenna and Charleston 1980 | | |  |
| Australia (Queensland) | 1979 | Stray | N.S. | 400 | 2 (0.5) | Microscopy | | | A | Wilson-Hanson and Prescott 1982 | | |  |
| New Zealand (Hastings) | 1984-1986 | Stray | N.S. | 63 | 0 (0.0) | Microscopy | | | A | Langham and Charleston 1990 | | |  |
| Australia (Western) | 1987 | N.S. | N.S. | 34 | 5 (14.7) | Microscopy | | | N.S. | Thompson et al. 1993 | | |  |
| **EUROPE** |  |  |  |  |  |  | | |  |  | | |  |
| Czec Republic (Prague) | 1975 | N.S. | N.S. | 382 | 0 (0.0) | Microscopy | | | N.S. | Vokoun and Slezakova 1977 | | |  |
| Germany (Hamburg) | 1977 | Pet¶ | N.S. | 308 | 4 (1.3) | Microscopy | | | S | Potters 1978 | | |  |
| Germany (South) | 1978-1979 | N.S. | N.S. | 694 | 4 (0.6) | Microscopy | | | N.S. | Boch and Walter 1979 | | |  |
| Scotland | 1987 | Stray | N.S. | 100 | 2 (2.0) | Microscopy | | | N.S. | McColm et al. 1981 | | |  |
| Belgium (Antwerp) | 1984 | Pet | 1m-14y | 150 | 4 (2.7) | Microscopy | | | N.S. | Beeck et al. 1985 | | |  |
| Czec Republic (Brno) | 1981-1984 | Stray | 16 d-1.5 y | 620 | 8 (1.29) | Microscopy | | | N.S. | Svobodova & Svoboda 1986 | | |  |
| France | 1984 | Stray | >1y; <1y | 300 | 18 (6.0) | Microscopy | | | N.S. | Pop et al. 1986 | | |  |
| UK (South-west) | 1986 | N.S. | N.S. | 51 | 0 (0.0) | Microscopy | | | N.S. | Gethings et al. 1987 | | |  |
| Germany | 1988 | N.S. | N.S. | 264 | 0 (0.0) | Microscopy | | | N.S. | Knaus and Fehler 1989 | | |  |
| Belgium | 1980-1990 | Stray | N.S. | 30 | 0 (0.0) | Microscopy | | | A | Vanparijs et al. 1991 | | |  |
| Germany (Lubeck) | 1989 | Pet | N.S. | 704 | 1 (0.2) | Mouse bioassay | | | N.S. | Unbehauen 1991 | | |  |
| Germany | 1990 | N.S. | N.S. | 70 | 12 (17.1) | Microscopy | | | N.S. | Beelitz et al. 1992 | | |  |
| Germany | 1991 | N.S. | N.S. | 2,472 | 26 (1.0) | Microscopy | | | N.S. | Epe et al. 1993 | | |  |
| Germany | 1993 | Stray | N.S. | 111 | 4 (3.6) | Microscopy | | | N.S. | Raschka et al. 1994 | | |  |
| Austria | 1994 | N.S. | N.S. | 1,368 | 27 (2.0) | Microscopy | | | N.S. | Edelhofer and Aspock 1996 | | |  |
| Czec Republic | 1995-1997 | Pet¶ | 3m-16 y | 390 | 0 (0.0) | Microscopy | | | A | Svobodova et al. 1998 | | |  |
| Germany (Hannover) | 1996-1997 | Stray | N.S. | 932 | 2 (0.2) | Microscopy | | | N.S. | Mundhenke and Daugschies 1999 | | |  |
| Germany (Freiburg) | 1999-2002 | Pet¶ | <1; 1-5; 5-10; >10 y | 2,473 | 22 (0.9) | Microscopy | | | S | Barutzki and Schaper 2003 | | |  |
| Germany | 2003 | N.S. | N.S. | 441 | 3 (7.0) | Microscopy | | | N.S. | Epe et al. 2004 | | |  |

**Table 1.** Continued

| **Location** | **Period** | **Stray/Pet** | **Age groups** | **Sample size** | **Positive (%)** | **Method** | **Symp.** | | **Author and publication date** |
| --- | --- | --- | --- | --- | --- | --- | --- | --- | --- |
| Spain (La Rioja, Madrid) | 2003 | Stray & pet | >6 m; <6 m | 382 | 0 (0.0) | Microscopy | A | Miró et al. 2004 | |
| France (Lyon) | 1993-2004 | Stray^d^ | N.S. | 322 | 0 (0.0) | Microscopy | A | Afonso et al. 2006 | |
| Austria | 2004-2006 | Pet† | N.S. | 994 | 1 (0.1) | Microscopy | S | Schares et al. 2008 | |
| Finland | 2004-2006 | Pet† | N.S. | 5 | 0 (0.0) | Microscopy | S | Schares et al. 2008 | |
| France | 2004-2006 | Pet† | N.S. | 858 | 2 (0.2) | Microscopy | S | Schares et al. 2008 | |
| Germany | 2004-2006 | Pet† | N.S. | 20,317 | 22 (0.11) | Microscopy | S | Schares et al. 2008 | |
| Hungary | 2004-2006 | Pet† | N.S. | 29 | 0 (0.0) | Microscopy | S | Schares et al. 2008 | |
| Italy | 2004-2006 | Pet† | N.S. | 257 | 0 (0.0) | Microscopy | S | Schares et al. 2008 | |
| Luxembourg | 2004-2006 | Pet† | N.S. | 159 | 0 (0.0) | Microscopy | S | Schares et al. 2008 | |
| Monaco | 2004-2006 | Pet† | N.S. | 5 | 0 (0.0) | Microscopy | S | Schares et al. 2008 | |
| Norway | 2004-2006 | Pet† | N.S. | 8 | 0 (0.0) | Microscopy | S | Schares et al. 2008 | |
| Netherlands | 2004-2006 | Pet† | N.S. | 966 | 0 (0.0) | Microscopy | S | Schares et al. 2008 | |
| Portugal | 2004-2006 | Pet† | N.S. | 11 | 0 (0.0) | Microscopy | S | Schares et al. 2008 | |
| Spain | 2006 | N.S. | N.S. | 592 | 0 (0.0) | Microscopy | N.S. | Montoya et al. 2008 | |
| Switzerland | 2004-2006 | Pet† | N.S. | 5 | 1 (20.0) | Microscopy | S | Schares et al. 2008 | |
| Sweden | 2004-2006 | Pet† | N.S. | 17 | 0 (0.0) | Microscopy | S | Schares et al. 2008 | |
| UK | 2004-2006 | Pet† | N.S. | 26 | 0 (0.0) | Microscopy | S | Schares et al. 2008 | |
| Italy (Florence) | 2009 | Stray | 6 m - 3 y | 50 | 8 (16.0) | Nested-PCR | A | Mancianti et al. 2010 | |
| Romania (Transylvania) | 2008 | Pet | N.S. | 440 | 5 (1.2) | Microscopy | N.S. | Mircean 2010 | |
| Romania (Arad) | 2010 | N.S. | N.S. | 36 | 0 (0.0) | Microscopy | N.S. | Hotea et al. 2011 | |
| Switzerland (Bern, Olten) | 2007-2008 | Stray & pet | <2 y; 2-10 y; >10 y | 252 | 1 (0.4) | PCR | A | Berger-Schoch et al. 2011 | |
| Finland (Helsinki) | 2008-2009 | Pet^d^ | Adult | 131 | 1 (76.3) | PCR | N.S. | Jokelainen et al. 2012 | |
| Germany (Lower Saxony) | 2010 | Stray & Foster | N.S. | 837 | 1 (0.1) | Microscopy | A | Becker et al. 2012 | |
| Italy (Milan) | 2011 | Stray | N.S. | 136 | 0 (0.0) | Microscopy | N.S. | Spada et al. 2013 | |
| Lativa (various areas) | 2011-2012 | Pet† | <1; 1-6; >7y | 80 | 2 (2.5) | Microscopy | N.S. | Deksne et al. 2013 | |
| Romania (Western) | 2009 | Stray & pet | N.S. | 605 | 1 (0.2) | Microscopy | N.S. | Hotea et al. 2013 | |
| Netherlands | 2012 | Animal shelters | N.S. | 305 | 1 (0.3) | Microscopy | A | Robben et al. 2014 | |
| Portugal (Lisbon) | 2007-2008 | Pet | 6-12; 12-24m | 45 | 16 (35.5) | PCR | N.S. | Esteves et al. 2014 | |
| UK | 2010-2014 | Pet | N.S. | 1,151 | 12 (1.0) | RT-PCR | S | Paris et al. 2014 | |
| Italy | 2013 | Pet | N.S. | 146 | 11 (7.5) | PCR | A | Mancianti et al. 2015 | |
| Greece (Crete Island) | 2011-2015 | Household | N.S. | 264 | 1 (0.4) | Microscopy | N.S. | Kostopoulou et al. 2017 | |
| Italy (Perugia) | 2014-2015 | Pet | N.S. | 78 | 16 (20.5) | PCR & Microscopy | S | Veronesi et al. 2017 | |

**Table 1.** Continued

| **Location** | **Period** | **Stray/Pet** | **Age groups** | **Sample size** | **Positive (%)** | **Methods** | **Symp.** | **Author and publication date** |
| --- | --- | --- | --- | --- | --- | --- | --- | --- |
| Sweden | 2015 | Outdoor | N.S. | 205 | 1 (0.5) | Microscopy | N.S. | Grandi et al. 2017 |
| Italy (Umbria) | 2014-2015 | Pet | 8 m-3 y | 77 | 13 (16.9) | PCR | S | Santoro et al. 2017 |
| Poland (Silesian) | 2017 | N.S. | 0.5-12y | 41 | 1 (2.4) | RT-PCR | N.S. | Sroka et al. 2018 |
| Spain (central) | 2014-2017 | Stray | ≤1; >1y | 356 | 0 (0.0) | Microscopy | A | Montoya et al. 2018 |
| **NORTH AMERICA** |  |  |  |  |  |  |  |  |
| USA (Kansas) | 1972 | Stray | Old; kitten | 510 | 0 (0.0) | Microscopy | A | Dubey 1973^e^ |
| USA (Ohio) | 1974 | Stray | N.S. | 1,000 | 10 (1.0) | Microscopy | N.S. | Christie et al. 1976 |
| USA (Ohio) | 1974-1975 | Stray | ≥3 m | 1,000 | 7 (0.7) | Microscopy | N.S. | Dubey et al. 1977 |
| USA (Washington) | 1981 | Stray & pet | N.S. | 73 | 0 (0.0) | Microscopy | S | Ladiges et al. 1982 |
| USA (Maryland) | 1984 | Stray | N.S. | 174 | 1 (0.5) | Microscopy | N.S. | Childs and Seegar 1986 |
| USA (Illinois) | 1992-1993 | Stray^g^ | N.S. | 274 | 5 (1.8) | Microscopy | A | Dubey et al. 1995 |
| Canada (Victoria) | 1997 | Pet^‡^ | N.S. | 26 | 0 (0.0) | Mouse bioassay | A | Aramini et al. 1999 |
| USA (Colorado) | 1993-1995 | Pet | <1; 1-3; 4-6; >10 y | 206 | 0 (0.0) | Microscopy | S | Hill et al. 2000 |
| USA (NY) | 1998-1999 | Pet | 1-12 m | 263 | 3 (1.1) | Microscopy | A | Spain et al. 2001 |
| USA (Connecticut) | 2002 | Pet^h^ | N.S. | 450 | 3 (0.7) | Microscopy | S | Rembiesa and Richardson 2003 |
| USA (California) | 2005 | Stray & pet | N.S. | 326 | 3 (0.9) | Microscopy | N.S. | Dabritz et al. 2007 |
| USA (Midwestern) | 2003-2005 | Stray^i^ | N.S. | 34 | 0 (0.0) | Microscopy | N.S. | de Camps et al. 2008 |
| USA (New York) | N.S. | Stray | N.S. | 1,322 | 11 (8.0) | Microscopy | N.S. | Lucio-Forster and Bowman 2011 |
| Canada (Prince Edward) | 2009 | Stray | Juveniles; adults | 78 | 1 (1.3) | Microscopy | N.S. | Stojanovic and Foley 2011 |
| USA (California) | 2006-2009 | Feral | Juveniles; adults | 452 | 10 (2.2) | Microscopy | N.S. | VanWormer et al. 2013 |
| USA (Virginia) | 2012 | Stray & pet | N.S. | 49 | 3 (6.1) | PCR Sequencing | N.S. | Lilly and Worthman 2013 |
| **CENTRAL/SOUTH AME.** |  |  |  |  |  |  |  |  |
| Hawaii | 1972 | Stray | N.S. | 1,604 | 12 (0.7) | Mouse bioassay | N.S. | Wallace 1973e |
| Mexico | 1976 | N.S. | N.S. | 200 | 14 (7.0) | Micro & bioassay | N.S. | de Aluja and Aguilar 1977 |
| Costa Rica (various areas) | 1979 | Stray & pet | N.S. | 237 | 55 (23.0) | Mouse bioassay | N.S. | Ruiz and Frenkel 1980e |
| Mexico | 1989 | N.S. | N.S. | 200 | 0 (0.0) | Microscopy | N.S. | Guevara Collazo et al. 1990 |
| Argentina | 1990 | N.S. | N.S. | 50 | 1 (2.0) | Micro & bioassay | N.S. | Venturini et al. 1992 |
| Panama (Panama city) | 1994 | Stray | N.S. | 383 | 2 (0.5) | Mouse bioassay | A | Frenkel et al. 1995e |
| Colombia | 1997 | N.S. | N.S. | 18 | 12 (66.6) | Microscopy | N.S. | Londono et al. 1998 |
| Brazil (São Paulo) | 2003 | Stray | 3-8 m; >1 y | 237 | 3 (1.2) | Mouse bioassay | A | Pena et al. 2006 |
| Colombia (Bogota) | 2005 | Stray | N.S. | 143 | 0 (0.0) | Microscopy | A | Dubey et al. 2006 |
| Puerto Rico (Mona Island) | 2004-2005 | Stray | Adult; kitten | 6 | 0 (0.0) | Microscopy | A | Dubey et al. 2007 |
| Peru (Lima) | 2013 | Pet | <1; 1-7; >7y | 50 | 0 (0.0) | Microscopy | S | Cerro et al. 2014 |
| Brazil (Goias) | 2015-2016 | Stray & pet | N.S. | 149 | 53 (35.5) | Microscopy | N.S. | Lima et al. 2018 |

†Samples from veterinary laboratory; ^‡^ Unwanted in animal protection/care center; ^¶^ Samples from small animal hospitals and clinics; ^d^ Faeces of stray cats in the park or ground; ^e^ Only abstract available; ^f^ Bought from Changban Free Market, Guangzhou, Guangdong Province, PRC, ^g^ Cats from pig farms; ^h^ The participating animal hospitals and shelter; ^i^ Zoo animals; N.S. = Not stated; A: Asymptomatic; S: Symptomatic (defined as the presence of diarrhoea)

**References**

1. Umeche, N. Studie kokcidii u kocek v oblasti Calabar v Nigerii. Veterinarstvi 1990; 40, 516-517.
2. Al-Kappany YM, Rajendran C, Ferreira LR, Kwok OC, Abu-Elwafa SA, Hilali M, Dubey JP. Seroprevalence of anti-*Toxoplasma gondii* antibodies in Egyptian sheep and goats. J Parasitol. 2010;96(6):1115-8
3. Dubey JP, Choudhary S, Tilahun G, Tiao N, Gebreyes WA, Zou X, Su C. Genetic diversity of *Toxoplasma gondii* isolates from Ethiopian feral cats. Vet Parasitol. 2013;196(1-2):206-8
4. Njuguna AN, Kagira JM, Karanja SM, Ngotho M, Mutharia L, Maina NW. Prevalence of *Toxoplasma gondii* and Other Gastrointestinal Parasites in Domestic Cats from Households in Thika Region, Kenya. 2017, ID 7615810
5. Ould Ahmed Salem CB, Mamadou DF, Hafid J. Study of prevalence of *Toxoplasma gondii* oocysts in the cats of Nouakchott. Bull Soc Pathol Exot. 2017;110(5):315-317
6. Samad, M.A., Dey, B.C., Chodhury, N.S., Akhter, S., Khan, M.R., 1997. Sero-epidemiological studies on *Toxoplasma gondii* infection in man and animals in Bangladesh. Southeast Asian J. Trop. Med. Public Health 28 (2), 339-343.
7. Dubey, J. P., Zhu, X. Q., Sundar, N., Zhang, H., Kwok, O. C., Su, C. Genetic and biologic characterization of *Toxoplasma gondii* isolates of cats from China. *Vet. Parasitol.* 2007;145, 352-356.
8. Qian W., Wang H., Su C., Shan D., Cui X., Yang N., et al. Isolation and characterization of Toxoplasma gondii strains from stray cats revealed a single genotype in Beijing, China. Vet. Parasitol. 2012;187, 408–413
9. Yang Y, Ying Y, Verma SK, Cassinelli AB, Kwok OC, Liang H, Pradhan AK, Zhu XQ, Su C, Dubey JP. Isolation and genetic characterization of viable *Toxoplasma gondii* from tissues and feces of cats from the central region of China. Vet Parasitol. 2015;211(3-4):283-8
10. Shastri, U.V., Ratnaparkhi, M.R., 1992. *Toxoplasma* and other intestinal coccidia in cats in Maharashtra (Parbhani). Indian Veterinary Journal 69, 14-16.
11. Hooshyar, H., Rostamkhani, P., Talari, S., Arbabi, M. Toxoplasma gondii infection in stray cats. Iranian Journal of Parasitology 2007;2, 18-22
12. Sharif, M., Daryani, A., Nasrolahei, M., Ziapour, S.P. Prevalence of Toxoplasma gondii antibodies in stray cats in Sari, northern Iran. Tropical Animal Health and Production 2009;41, 183-187
13. Mosallanejad B., Avizeh R., Razi Jalali MH., Pourmehdi M. A study on seroprevalence and coproantigen detection of *Toxoplasma gondii* in companion cats in Ahvaz area, southwestern Iran. Iran J Vet Res. 2011; 12(2):139-144.
14. Salant, H., Markovics, A., Spira, D.T., Hamburger, J., 2007. The development of a molecular approach for coprodiagnosis of *Toxoplasma* *gondii*. Veterinary Parasitology 146, 214-220.
15. Oikawa, H., Omata, Y., Kanda, M., Mikazuki, K., Yano, K., Nakabayashi, T., 1990. Survey on *Toxoplasma* infection in stray cats in western area of Japan during a two-year period. Japanese Journal of Parasitology 39, 462-467.
16. Nabi, H., Rashid, M.I., Islam, S., Bajwa, A.A., Gul, R., Shehzad, W., et al., 2018. Prevalence of *Toxoplasma gondii* oocysts through Copro-PCR in cats at Pet Center (UVAS), Lahore, Pakistan. J. Pak. Med. Assoc. 68, 115-118.
17. Chong, L.H., Singh, M., Chua, S.B., Fong, W.E., 1993. Feline toxoplasmosis in Singapore. Singapore Veterinary Journal 17, 79-87.
18. Lin, D.S., Lai, S.S., Bowman, D.D., Jacobson, R.H., Barr, M.C., Giovengo, S.L., 1990. Feline immunodeficiency virus, feline leukemia virus, *Toxoplasma gondii*, and intestinal parasitic infections in Taiwanese cats. British Veterinary Journal 146, 468-475.
19. Karatepe, B., Babur, C., Karatepe, M., Kilic, S., Dundar, B., 2008. Prevalence of *Toxoplasma gondii* and intestinal parasites in stray cats from Nigde, Turkey. Italian Journal of Animal Science 7, 113-118.
20. Langham, N.P.E., Charleston, W.A.G., 1990. An investigation of the potential for spread of Sarcocystis spp. and other parasites by feral cats. New Zealand Journal of Agriculture Research 33, 429-435.
21. Edelhofer, R., Aspock, H., 1996. Infektionsquellen und Infektionswege aus der Sicht des Toxoplasmose-Screenings der Schwangeren in Osterreich. Mitteilungen Osterreichischen Gesellschaft fur Tropenmedizin und Parasitolologie 18, 59–70.
22. Schares, G., Vrhovec, M.G., Pantchev, N., Herrmann, D.C., Conraths, F.J., 2008a. Occurrence of *Toxoplasma gondii* and *Hammondia* *hammondi* oocysts in the faeces of cats from Germany and other European countries. Veterinary Parasitology 152, 34-45
23. Vanparijs, O., Hermans, L., van der Flaes, L., 1991. Helminth and protozoan parasites in dogs and cats in Belgium. Veterinary Parasitology 38, 67-73.
24. Svobodova, V., Knotek, Z., Svoboda, M., 1998. Prevalence of IgG and IgM antibodies specific to *Toxoplasma gondii* in cats. Veterinary Parasitology 80, 173-176.
25. Afonso, E., Thulliez, P., Gilot-Fromont, E., 2006. Transmission of *Toxoplasma gondii* in an urban population of domestic cats (*Felis catus*). International Journal for Parasitology 36, 1373-1382.
26. Knaus, B.U., Fehler, K., 1989. *Toxoplasma gondii*-Infektionen und Oozystenausscheidung bei Hauskatzen und ihre Bedeutung fur die Epidemiologie und Epizootiologie der Toxoplasmose. Angewandte Parastiologie 30, 155-160.
27. Beelitz, P., Gobel, E., Gothe, R., 1992. Fauna und Befallshaufigkeit von Endoparasiten bei Katzenwelpen und ihren Muttern unterschiedlicher Haltung in Suddeutschland. Tierarztliche Praxis 20, 297-300.
28. Epe, C., Ising-Volmer, S., Stoye, M., 1993. Ergebnisse parasitologischer Kotuntersuchungen von Equiden, Hunden, Katzen un Igeln der Jahre 1984-1991. Deutsche Tierarztliche Wochenschrift 100, 426-428
29. Barutzki, D., Schaper, R., 2003. Endoparasites in dogs and cats in Germany 1999– 2002. Parasitology Research 90, S148-S150
30. Epe, C., Coati, N., Schnieder, T., 2004. Ergebnisse parasitologischer Kotuntersuchungen von Pferden, Wiederkauern, Schweinen, Hunden, Katzen, Igeln un Kaninchen in den Jahren 1998-2002. Deutsche Tierarztliche Wochenschrift 111, 243-247.
31. Veronesi, F., Santoro, A., Milardi, G.L., Diaferia, M., Morganti, G., Ranucci, D., Gabrielli, S., 2017. Detection of *Toxoplasma gondii* in faeces of privately owned cats using two PCR assays targeting the B1 gene and the 529-bp repetitive element. Parasitol. Res. 116, 1063–1069
32. Miro, G., Montoya, A., Jimenez, S., Frisuelos, C., Mateo, M., Fuentes, I., 2004. Prevalence of antibodies to *Toxoplasma gondii* and intestinal parasites in stray, farm and household cats in Spain. Veterinary Parasitology 126, 249–255.
33. Montoya, A., Miro, G., Mateo, M., Ramirez, C., Fuentes, I., 2008. Molecular characterization of *Toxoplasma gondii* isolates from cats from Spain. Journal of Parasitology 94, 1044-1046.
34. Berger-Schoch, A.E., Herrmann, D.C., Schares, G., Muller, N., Bernet, D., Gottstein, B., Frey, C.F., 2011. Prevalence and genotypes of *Toxoplasma gondii* in feline faeces (oocysts) and meat from sheep, cattle and pigs in Switzerland. Vet. Parasitol. 177, 290-297.
35. Aramini, J.J., Stephen, C., Dubey, J.P., Engelstoft, C., Schwantje, H., Ribble, C.S., 1999. Potential contamination of drinking water with *Toxoplasma gondii* oocysts. Epidemiol. Infect. 122, 305–315.
36. Hill, S.L., Cheney, J.M., Taton-Allen, G.F., Reif, J.S., Bruns, C., Lappin, M.R., 2000. Prevalence of enteric zoonotic organisms in cats. Journal of the American Veterinary Medical Association 216, 687–692.
37. Spain, C.V., Scarlett, J.M., Wade, S.E., McDonough, P., 2001. Prevalence of enteric zoonotic agents in cats less than 1 year old in central New York state. Journal of Veterinary Internal Medicine 15, 33–38
38. Rembiesa, C., Richardson, D.J., 2003. Helminth parasites of the house cat, Felis catus, in Connecticut, U.S.A. Comparative Parasitology 70, 115–119.
39. Dabritz, H.A., Miller, M.A., Atwill, E.R., Gardner, I.A., Leutenegger, C.M., Melli, A.C., Conrad, P.A., 2007b. Detection of *Toxoplasma gondii*-like oocysts in cat feces and estimates of the environmental oocyst burden. Journal of the American Veterinary Medical Association 231, 1676–1684.
40. de Camps, S., Dubey, J.P., Saville, W.J.A., 2008. Seroepidemiology of *Toxoplasma gondii* in zoo animals in selected zoos in the midwestern United States. Journal of Parasitology 94, 648–653
41. Lilly, E.L.,Wortham, C.D., 2013. High prevalence of *Toxoplasma gondii* oocyst shedding in stray and pet cats (*Felis catus*) in Virginia, United States. Parasit. Vectors 6, 4.
42. Ruiz, A., Frenkel, J.K., 1980. *Toxoplasma gondii* in Costa Rican cats. Am. J. Trop. Med. Hyg. 29, 1150–1160.
43. Wallace, G.D., 1973. Intermediate and transport hosts in natural history of *Toxoplasma gondii*. Am. J. Trop. Med. Hyg. 22, 456–464.
44. Frenkel, J.K., Hassanein, K.M., Hassanein, R.S., Brown, E., Thulliez, P., Quinteronunez, R., 1995. Transmission of *Toxoplasma gondii* in Panama City, Panama - a 5-year prospective cohort study of children, cats, rodents, birds, and soil. Am. J. Trop. Med. Hyg. 53, 458–468.
45. Venturini, L., Venturini, M.C., Omata, Y., 1992. Diagnostico de toxoplasmosis durante el periodo patente en un gato domestico. Veterinaria Argentina 9, 528– 531.
46. Pena, H.F.J., Soares, R.M., Amaku, M., Dubey, J.P., Gennari, S.M., 2006. *Toxoplasma gondii* infection in cats from Sao Paulo state, Brazil: seroprevalence, oocyst shedding, isolation in mice, and biologic and molecular characterization. Research in Veterinary Science 81, 58–67
47. Londono, M.T.M., Chamorro, N.L., Infante, M.S., Carlos, J., Osorio, C., 1998. Infeccion por *Toxoplasma gondii* en gatos de dos barrios del sur de Armenia y su importancia en la toxoplasmosis humana. Colbaquin Actualidades Clinicas y Biotecnologicas 12, 18–23
48. de Aluja, A.S., Aguilar, P., 1977. Estudio sobre la frecuencia del ooquiste de *Toxoplasma gondii* en el gato domestico del distrito federal. Gaceta Medica de Mexico 113, 455–459
49. Guevara Collazo, G.D., Torres Montoya, J.T., Waissman, J.C., 1990. Situacion de los ooquistes de *Toxoplasma gondii* en heces de gatos del Distrito Federal, Mexico. Veterinaria Mexico 21, 45-48
50. Ladiges WC. et al. Prevalence of *Toxoplasma gondii* antibodies and oocysts in pound-source cats. [J Am Vet Med Assoc.](https://www.ncbi.nlm.nih.gov/pubmed/7096176) 1982;180(11):1334-5
51. [Tavalla](https://www.sciencedirect.com/science/article/abs/pii/S2468045117300652#!) M, [Asgarian](https://www.sciencedirect.com/science/article/abs/pii/S2468045117300652#!) F, Kazemi F. Prevalence and genetic diversity of *Toxoplasma gondii* oocysts in cats of southwest of Iran. Infection, Disease & Health 2017; 22(4):1-7
52. Raeghi S, Sedighi S, Sedighi S. Prevalence of *Toxoplasma gondii* antibodies in cats in Urmia, Northwest of Iran. The Journal of Animal & Plant Sciences, 2011;21(2):132-134
53. Hata H, Aosai F, Norose K, Kobayashi M, Mun HS, Chen M, Ito I, Isegawa N, Ishikawa M, Mori S, Yano A. Prevalence of *Toxoplasma gondii* and other intestinal parasites in cats in Chiba Preference Japan. Jpn. J. Trop. Med. Hyg., 2000; 28(4):365-368
54. Salman D, Pumidonming W, Oohashi E, Igarashi M. Prevalence of *Toxoplasma gondii* and other intestinal parasites in cats in Tokachi sub-prefecture, Japan. J Vet Med Sci. 2018;29;80(6):960-967
55. Jung BK, Lee SE, Lim H, Cho J, Kim DG, Song H, Kim MJ, Shin EH, Chai JY. *Toxoplasma gondii* B1 Gene Detection in Feces of Stray Cats around Seoul, Korea and Genotype Analysis of Two Laboratory-Passaged Isolates. Korean J Parasitol. 2015;53(3):259-63
56. Kim SE, Choi R, Kang SW, Hyun C. Prevalence of *Toxoplasma gondii* infection in household and feral cats in Korea. J Parasit Dis. 2017;41(3):823-825
57. ME Abdou, Al-Batel MK, El-Azazy OME, Attia MS, Majeed QAH. Enteric Protozoan Parasites in Stray Cats in Kuwait with Special References to Toxoplasmosis and Risk Factors Affecting Its Occurrence. J. Egypt. Soc. Parasitol., 2013;43(2):303-314
58. Abu-Madi MA, Behnke JM. Feline patent *Toxoplasma*-like coccidiosis among feral cats (*Felis catus*) in Doha city, Qatar and its immediate surroundings. Acta Parasitol. 2014;59(3):390-7
59. Jokelainen P, Simola O, Rantanen E, Näreaho A, Lohi H, Sukura A. Feline toxoplasmosis in Finland: cross-sectional epidemiological study and case series study. J Vet Diagn Invest. 2012;24(6):1115-24.
60. Mancianti F, Nardoni S, Ariti G, Parlanti D, Giuliani G, Papini RA. Cross-sectional survey of *Toxoplasma gondii* infection in colony cats from urban Florence (Italy). J Feline Med Surg. 2010;12(4):351-4
61. Santoro A, Veronesi F, Milardi GL, Ranucci D, Branciari R, Diaferia M, Gabrielli S. Sequence variation in the B1 gene among *Toxoplasma gondii* isolates from swine and cats in Italy. Res Vet Sci. 2017;115:353-355
62. Deksne G, Petrusēviča A, Kirjušina M. Seroprevalence and factors associated with *Toxoplasma gondii* infection in domestic cats from urban areas in Latvia. J Parasitol. 2013;99(1):48-50
63. Esteves F, Aguiar D, Rosado J, Costa ML, de Sousa B, Antunes F, Matos O*. Toxoplasma gondii* prevalence in cats from Lisbon and in pigs from centre and south of Portugal. Vet Parasitol. 2014;200(1-2):8-12.
64. Sroka J, Karamon J, Dutkiewicz J, Wójcik Fatla A, Zając V, Cencek T. Prevalence of *Toxoplasma* *gondii* infection in cats in southwestern Poland. Ann Agric Environ Med. 2018;25(3):576-580
65. Montoya A, García M, Gálvez R, Checa R, Marino V, Sarquis J, Barrera JP, Rupérez C, Caballero L, Chicharro C, Cruz I, Miró G. Implications of zoonotic and vector-borne parasites to free-roaming cats in central Spain. Vet Parasitol. 2018;251:125-130
66. Dubey JP. Feline toxoplasmosis and coccidiosis: a survey of domiciled and stray cats. J Am Vet Med Assoc. 1973;162(10):873-7
67. Dubey JP, Christie E, Pappas PW. Characterization of *Toxoplasma gondii* from the feces of naturally infected cats. J Infect Dis. 1977;136(3):432-5
68. Dubey JP, Weigel RM, Siegel AM, Thulliez P, Kitron UD, Mitchell MA, Mannelli A, Mateus-Pinilla NE, Shen SK, Kwok OC, et al. Sources and reservoirs of Toxoplasma gondii infection on 47 swine farms in Illinois. J Parasitol. 1995;81(5):723-9
69. Dubey JP, López-Torres HY, Sundar N, Velmurugan GV, Ajzenberg D, Kwok OC, Hill R, Dardé ML, Su C. Mouse-virulent *Toxoplasma gondii* isolated from feral cats on Mona Island, Puerto Rico. J Parasitol. 2007;93(6):1365-9
70. Dubey JP, Su C, Cortés JA, Sundar N, Gomez-Marin JE, Polo LJ, Zambrano L, Mora LE, Lora F, Jimenez J, Kwok OC, Shen SK, Zhang X, Nieto A, Thulliez P. Prevalence of *Toxoplasma gondii* in cats from Colombia, South America and genetic characterization of *T. gondii* isolates. Vet Parasitol. 2006;141(1-2):42-7
71. Cerro L, Rubio A, Pinedo R, Mendes-de-Almeida F, Brener B, Labarthe N. Seroprevalence of *Toxoplasma gondii* in cats (*Felis catus*, Linnaeus 1758) living in Lima, Peru. Rev Bras Parasitol Vet. 2014;23(1):90-3.
72. Stojanovic V, Foley P. Infectious disease prevalence in a feral cat population on Prince Edward Island, Canada. Can Vet J 2011;52:979-982
73. Tutuncu M, Akkan HA, Karaca M, Agaoglu Z, Berktas M. Prevalence of toxoplasmosis in Van cats in Turkey. The Indian veterinary journal 80(8):730-732
74. Svobodova V, Svoboda M. Incidence of *Toxoplasma gondii* oocysts in cat feces. Veterinární medicína 1986;31(10):621-8
75. McColm AA, Hutchison WM, Siim JC. The prevalence of *Toxoplasma gondii* in meat animals and cats in central Scotland. Annals of Tropical Medicine & Parasitology. 1981; 75(2):157-164
76. Werner JK, Walton BC. [Prevalence of naturally occurring *Toxoplasma gondii* infections in cats from U.S. military installations in Japan.](http://ovidsp.tx.ovid.com/sp-3.31.1b/ovidweb.cgi?&S=IIPAFPDDHLDDEIPFNCEKOBGCCILLAA00&Complete+Reference=S.sh.69|35|1)  J Parasitol. 1972;58(6):1148-1150.
77. Beeck LV, Henry MC, Dorny P, Meirvenne NV. [Prevalence of *Toxoplasma* *gondii* and *Toxocara* *cati* infections in cats in the Antwerp urban area.](http://ovidsp.tx.ovid.com/sp-3.31.1b/ovidweb.cgi?&S=IIPAFPDDHLDDEIPFNCEKOBGCCILLAA00&Complete+Reference=S.sh.69|37|1)  Annales de Medecine Veterinaire; 1985;129(6):433-440
78. Pop A, Cerbu A, Pop A, Andreescu N. The seasonal prevalence of the *Toxoplasma gondii* infections in stray cats from urban area studied by parasitological methods. Arch Roum Pathol Exp Microbiol. 1986;45(1):57-63.
79. Arene FOI. The prevalence and public health significance of *Toxoplasma gondii* in domestic cats in the Niger Delta. Public Health. 1984;98(6):333-5.
80. Vanwormer E, Conrad PA, Miller MA, Melli AC, Carpenter TE, Mazet JA*. Toxoplasma gondii*, source to sea: higher contribution of domestic felids to terrestrial parasite loading despite lower infection prevalence. Eco health. 2013;10(3):277-89
81. Spada E, Proverbio D, Pepa A, della Domenichini G, Giorgi GB, de Traldi G, Ferro E. [Prevalence of faecal-borne parasites in colony stray cats in northern Italy.](http://ovidsp.tx.ovid.com/sp-3.31.1b/ovidweb.cgi?&S=IIPAFPDDHLDDEIPFNCEKOBGCCILLAA00&Complete+Reference=S.sh.69|64|1)  Journal of Feline Medicine and Surgery 2013;15(8):672-677
82. Borkataki S, Katoch R, Goswami P, Godara R, Khajuria, JK, Yadav A, Kaur R. [Prevalence of parasitic infections of stray cats in Jammu, India.](http://ovidsp.tx.ovid.com/sp-3.31.1b/ovidweb.cgi?&S=IIPAFPDDHLDDEIPFNCEKOBGCCILLAA00&Complete+Reference=S.sh.69|65|1) Sokoto Journal of Veterinary Sciences 2013;11(1):1-6
83. Becker AC, Rohen M, Epe C, Schnieder T. [Prevalence of endoparasites in stray and fostered dogs and cats in Northern Germany.](http://ovidsp.tx.ovid.com/sp-3.31.1b/ovidweb.cgi?&S=IIPAFPDDHLDDEIPFNCEKOBGCCILLAA00&Complete+Reference=S.sh.69|66|1) Parasitology Research 2012;111(2):849-857
84. Grandi G, Comin A, Ibrahim O, Schaper R, Forshell U, Lind EO. [Prevalence of helminth and coccidian parasites in Swedish outdoor cats and the first report of *Aelurostrongylus abstrusus* in Sweden: a coprological investigation.](http://ovidsp.tx.ovid.com/sp-3.31.1b/ovidweb.cgi?&S=IIPAFPDDHLDDEIPFNCEKOBGCCILLAA00&Complete+Reference=S.sh.69|68|1)  Acta Veterinaria Scandinavica 2017;59(19)
85. Hotea I, Oprescu I, Ilie MS, Imre K, Imre M, Darabus G. [Seroprevalence of *Toxoplasma gondii* infection in cats and sheep in Arad County.](http://ovidsp.tx.ovid.com/sp-3.31.1b/ovidweb.cgi?&S=IIPAFPDDHLDDEIPFNCEKOBGCCILLAA00&Complete+Reference=S.sh.69|80|1)  Medicina Veterinara 2011;44(1):63-68
86. Boch J, Walter D. [Four species of coccidia (*Isospora, Toxoplasma, Sarcocystis*) of cats in South Germany.](http://ovidsp.tx.ovid.com/sp-3.31.1b/ovidweb.cgi?&S=IIPAFPDDHLDDEIPFNCEKOBGCCILLAA00&Complete+Reference=S.sh.69|85|1) Tierarztliche Umschau 1979;34(11):749-752.
87. Mundhenke H, Daugschies A. [Studies on the prevalence of endoparasites in cats in Hannover and surroundings.](http://ovidsp.tx.ovid.com/sp-3.31.1b/ovidweb.cgi?&S=IIPAFPDDHLDDEIPFNCEKOBGCCILLAA00&Complete+Reference=S.sh.69|89|1) Wiener Tierarztliche Monatsschrift 1999;86(2):43-48.
88. Unbehauen I. [Prevalence of intestinal parasites in cats in the Lubeck area.](http://ovidsp.tx.ovid.com/sp-3.31.1b/ovidweb.cgi?&S=IIPAFPDDHLDDEIPFNCEKOBGCCILLAA00&Complete+Reference=S.sh.69|90|1) [Germany] 1991:106 pp. 28 pp. of ref. [Thesis]
89. Christie E, Dubey JP. Pappas PW. [Prevalence of Sarcocystis infection and other intestinal parasitisms in cats from a humane shelter in Ohio.](http://ovidsp.tx.ovid.com/sp-3.31.1b/ovidweb.cgi?&S=IIPAFPDDHLDDEIPFNCEKOBGCCILLAA00&Complete+Reference=S.sh.69|91|1)  Journal of the American Veterinary Medical Association 1976;168(5):421-422.
90. Lucio-Forster A, Bowman DD, [Prevalence of fecal-borne parasites detected by centrifugal flotation in feline samples from two shelters in upstate New York.](http://ovidsp.tx.ovid.com/sp-3.31.1b/ovidweb.cgi?&S=IIPAFPDDHLDDEIPFNCEKOBGCCILLAA00&Complete+Reference=S.sh.69|137|1)  Journal of Feline Medicine and Surgery 2011;13(4):300-303.
91. Potters U. [Investigations on the frequency of coccidial oocysts and sporocysts (Eimeriidae, Toxoplasmidae, Sarcocystidae) in the faeces of carnivores. [German]](http://ovidsp.tx.ovid.com/sp-3.31.1b/ovidweb.cgi?&S=IIPAFPDDHLDDEIPFNCEKOBGCCILLAA00&Complete+Reference=S.sh.69|150|1)  1978. :86 pp. [Thesis]
92. Childs JE, Seegar WS. [Epidemiologic observations on infection with *Toxoplasma gondii* in three species of urban mammals from Baltimore, Maryland, USA.](http://ovidsp.tx.ovid.com/sp-3.31.1b/ovidweb.cgi?&S=IIPAFPDDHLDDEIPFNCEKOBGCCILLAA00&Complete+Reference=S.sh.69|154|1)  International Journal of Zoonoses 1986;13(4):249-261.
93. Vokoun P, Slezakova J. [Parasites of the gastrointestinal tract of city dogs and cats.](http://ovidsp.tx.ovid.com/sp-3.31.1b/ovidweb.cgi?&S=IIPAFPDDHLDDEIPFNCEKOBGCCILLAA00&Complete+Reference=S.sh.69|155|1) Veterinarni Medicina 1977;22(6):367-376.
94. Raschka C, Haupt W, Ribbeck R. [Endoparasites of stray cats.](http://ovidsp.tx.ovid.com/sp-3.31.1b/ovidweb.cgi?&S=IIPAFPDDHLDDEIPFNCEKOBGCCILLAA00&Complete+Reference=S.sh.69|157|1) Monatshefte fur Veterinarmedizin 1994;49(7):307-315.
95. Razmi GR. [Prevalence of feline coccidia in Khorasan Province of Iran.](http://ovidsp.tx.ovid.com/sp-3.31.1b/ovidweb.cgi?&S=IIPAFPDDHLDDEIPFNCEKOBGCCILLAA00&Complete+Reference=S.sh.69|161|1)  Journal of Protozoology Research 1999;9(3):88-90.
96. Mircean V, Titilincu A, Vacile C. Prevalence of endoparasites in household cat (*Felis catus*) populations from Transylvania (Romania) and association with risk factors. Vet Parasitol. 2010;171(1-2):163-6
97. Hotea I, Ilie MS, Imre M, Sorescu D, Colibar O, Tirziu E, Seres M, Darabus G. [Prevalence of *Toxoplasma gondii* and intestinal parasites in stray and household cats in Western Romania.](http://ovidsp.tx.ovid.com/sp-3.31.1b/ovidweb.cgi?&S=IIPAFPDDHLDDEIPFNCEKOBGCCILLAA00&Complete+Reference=S.sh.69|374|1)  Medicina Veterinara 2013;46(3):85-90.
98. Gethings PM, Stephens GL, Wills JM, Howard P, Balfour AH, Wright AI, Morgan KL. [Prevalence of *Chlamydia, Toxoplasma, Toxocara* and ringworm in farm cats in south-west England.](http://ovidsp.tx.ovid.com/sp-3.31.1b/ovidweb.cgi?&S=IIPAFPDDHLDDEIPFNCEKOBGCCILLAA00&Complete+Reference=S.sh.69|390|1)  Veterinary Record 1987;121(10):213-216.
99. Khodaverdi M, Razmi Gh. A serological and parasitological study of *Toxoplasma gondii* infection in stray cats of Mashhad, Khorasan Razavi province, Iran. Veterinary Research Forum 2019;10(2):119-123
100. Mohammed OB, Omar OI, Elamin EA, Bushara HO, Omer SA, Alagaili AN. Seroprevalence of *Toxoplasma gondii* in household and stray cats of Riyadh, Saudi Arabia. Vet Ital. 2019;55(3):241-245.
101. Beigi PK, Nourollahi Fard SR, Akhtardanesh B. Prevalence of Zoonotic and Other Intestinal Protozoan Parasites in Stray Cats (*Felis domesticus*) of Kerman, South-East of Iran. Istanbul Universitesi Veteriner Fakultesi Dergisi 2017;43(1):23-27
102. Esmaeilzadeh M, Shamsfard M, Kazemi A, Khalafi SA, Altome SA. Prevalence of Protozoa and Gastrointestinal Helminthes in Stray Cats in Zanjan Province, North-West of Iran. Iranian J Parasitol. 2009;4(3):71-75
103. McKenna PB, Charleston WAG. Coccidia (Protozoa: Sporozoasida) of cats and dogs. I. Identity and prevalence in cats. New Zealand Veterinary Journal 1980;28(5):86-88
104. Asgari Q, Mohammadpour I, Pirzad R, Kalantari M, Motazedian MH, Naderi Sh. Molecular and Serological Detection of *Toxoplasma gondii* in Stray Cats in Shiraz, South-central, Iran. Iran J Parasitol. 2018;13(3): 430-439
105. Lima JAS. Rezende HHA. Rocha TMDD. Castro AM. [Analysis of the accuracy of different laboratory methods for the diagnosis of intestinal parasites from stray and domiciled cats (*Felis catus domesticus*) in Goiania, Goias, Brazil.](http://ovidsp.tx.ovid.com/sp-3.31.1b/ovidweb.cgi?&S=IIPAFPDDHLDDEIPFNCEKOBGCCILLAA00&Complete+Reference=S.sh.69|642|1)  Brazilian Journal of Veterinary Parasitology 2018;27(1):94-97.
106. Kostopoulou D, Claerebout E, Arvanitis D, Ligda P, Voutzourakis N, Casaert S, Sotiraki S. [Abundance, zoonotic potential and risk factors of intestinal parasitism amongst dog and cat populations: the scenario of Crete, Greece.](http://ovidsp.tx.ovid.com/sp-3.31.1b/ovidweb.cgi?&S=IIPAFPDDHLDDEIPFNCEKOBGCCILLAA00&Complete+Reference=S.sh.69|644|1)  Parasites and Vectors 2017;10(43)
107. Mancianti F, Nardoni S, Mugnaini L, Zambernardi L, Guerrini A, Gazzola V, Papini RA. A retrospective molecular study of select intestinal protozoa in healthy pet cats from Italy. J Feline Med Surg. 2015;17(2):163-7
108. Paris JK, Wills S, Balzer HJ, Shaw DJ, Gunn-Moore DA. Enteropathogen co-infection in UK cats with diarrhoea. BMC Vet Res. 2014;10:13.
109. Khademvatan S, Abdizadeh R, Rahim F, Hashemitabar M, Ghasemi M, Tavalla M. [Stray cats gastrointestinal parasites and its association with public health in Ahvaz City, South Western of Iran. Jundishapur Journal of Microbiology 2014;7(8):e11079.](http://ovidsp.tx.ovid.com/sp-3.31.1b/ovidweb.cgi?&S=IIPAFPDDHLDDEIPFNCEKOBGCCILLAA00&Complete+Reference=S.sh.69|652|1)
110. Robben SRM, Nobel WE, le Dopfer D, Hendrikx WML, Boersema JH, Fransen F, Eysker ME. [Infections with helminths and/or protozoa in cats in animal shelters in the Netherlands. [Dutch].](http://ovidsp.tx.ovid.com/sp-3.31.1b/ovidweb.cgi?&S=IIPAFPDDHLDDEIPFNCEKOBGCCILLAA00&Complete+Reference=S.sh.69|660|1)  Tijdschrift voor Diergeneeskunde 2004;129(1):2-6.
111. Thompson RCA, Meloni BP, Hopkins RM, Deplazes P, Reynoldson JA. [Observations on the endo- and ectoparasites affecting dogs and cats in Aboriginal communities in the north-west of Western Australia.](http://ovidsp.tx.ovid.com/sp-3.31.1b/ovidweb.cgi?&S=IIPAFPDDHLDDEIPFNCEKOBGCCILLAA00&Complete+Reference=S.sh.69|664|1)  Australian Veterinary Journal 1993;70(7):268-270.
112. Wilson-Hanson SL, Prescott CW. [A survey for parasites in cats.](http://ovidsp.tx.ovid.com/sp-3.31.1b/ovidweb.cgi?&S=IIPAFPDDHLDDEIPFNCEKOBGCCILLAA00&Complete+Reference=S.sh.69|668|1)  Australian Veterinary Journal 1982;59(6):194.
