# Supplementary material for: Toxoplasma gondii infection in domestic and wild felids as public health concerns: a systematic review and meta-analysis
Source: Sci Rep. 2021 May 4;11:9509. doi: 10.1038/s41598-021-89031-8 (PMC8097069; doi:10.1038/s41598-021-89031-8)
Supplement: Supplementary file 7 — Supplementary Information 7. [file 41598_2021_89031_MOESM7_ESM.doc]

**Table S5.** Characteristics of the eligible studies based on detection of *T. gondii*-like oocyst and *T. gondii* oocyst DNA in wild felids feces

| **Species** | **Location** | **Period** | **Status** | **Sample size** | **Positive (%)** | **Method** | **Ref.** |
| --- | --- | --- | --- | --- | --- | --- | --- |
| **Jaguar** |  |  |  |  |  |  |  |
| *Panthera onca*^a^ | Central America^*^ | 1985 | Free ranging | 25 | 1 (4.0) | Microscopy | Patton et al. 1986 |
| *Panthera onca* | Czech Republic | 1995-1996 | Captive^**^ | 88 | 0 (0.0) | Microscopy | Lukesova and Literak 1998 |
| *Panthera onca* | Mexico^f^ | 2015-2016 | Captive | 9 | 1 (11.1) | Nested PCR | Gomez-Rios et al. 2019 |
| **Jaguarundi** |  |  |  |  |  |  |  |
| *Herpailurus yagouaroundi* | Central America^*^ | 1985 | Free ranging | 2 | 0 (0.0) | Microscopy | Patton et al. 1986 |
| *Herpailurus yagouaroundi* | Czech Republic | 1995-1996 | Captive^**^ | 13 | 0 (0.0) | Microscopy | Lukesova and Literak 1998 |
| *Herpailurus yagouaroundi* | Mexico^f^ | 2015-2016 | Captive | 1 | 0 (0.0) | Nested PCR | Gomez-Rios et al. 2019 |
| **Ocelot** |  |  |  |  |  |  |  |
| *Leopardus pardalis* | Central America^*^ | 1985 | Free ranging | 8 | 2 (25.0) | Microscopy | Patton et al. 1986 |
| *Leopardus pardalis* | Czech Republic | 1995-1996 | Captive^**^ | 91 | 0 (0.0) | Microscopy | Lukesova and Literak 1998 |
| *Leopardus pardalis* | Mexico^f^ | 2015-2016 | Captive | 2 | 1 (50.0) | Nested PCR | Gomez-Rios et al. 2019 |
| **Cougar** |  |  |  |  |  |  |  |
| *Puma concolor* | USA | 1975 | Free ranging | 5 | 0 (0.0) | Mouse bioassay | Marchiondo et al. 1976 |
| *Puma concolor* | Central America^*^ | 1985 | Free ranging | 4 | 0 (0.0) | Microscopy | Patton et al. 1986 |
| *Puma concolor* | Czech Republic | 1995-1996 | Captive^**^ | 110 | 0 (0.0) | Microscopy | Lukesova and Literak 1998 |
| *Puma concolor* | Canada^†^ | 1995 | Free ranging | 23 | 2 (8.7)^¶^ | Mouse bioassay | Aramini et al. 1998 |
| *Puma concolor* | USA (California) | 2006-2009 | Free ranging | 51 | 2 (3.9) | Microscopy | VanWormer et al. 2013 |
| *Puma concolor* | Mexico^f^ | 2015-2016 | Capture | 3 | 1 (33.3) | Nested PCR | Gomez-Rios et al. 2019 |
| **Prionailurus cats** |  |  |  |  |  |  |  |
| *Prionailurus iriomotensis* | Japan^b^ | 1983-1985 | Free ranging | 45^d^ | 2 (4.4) | Mouse bioassay | Akuzawa et al. 1987 |
| *Prionailurus bengalensis* | Thailand^‡^ | 1987-1989 | Free ranging | 3 | 0 (0.0) | Microscopy | Patton and Rabinowitz 1994 |
| *Prionailurus bengalensis* | Czech Republic | 1995-1996 | Captive^**^ | 1 | 0 (0.0) | Microscopy | Lukesova and Literak 1998 |
| *Prionailurus bengalensis euptilura* | Czech Republic | 1995-1996 | Captive^**^ | 133 | 5 (4.0) | Microscopy | Lukesova and Literak 1998 |
| *Prionailurus viverrinus* | Czech Republic | 1995-1996 | Captive^**^ | 22 | 0 (0.0) | Microscopy | Lukesova and Literak 1998 |
| **Leopard** |  |  |  |  |  |  |  |
| *Panthera pardus* | Thailand^‡^ | 1987-1989 | Free ranging | 54 | 1 (1.9) | Microscopy | Patton and Rabinowitz 1994 |
| *Panthera pardus* | Czech Republic | 1995-1996 | Captive^**^ | 257 | 0 (0.0) | Microscopy | Lukesova and Literak 1998 |
| *Panthera pardus* | Mexico^f^ | 2015-2016 | Captive | 1 | 1 (100) | Nested PCR | Gomez-Rios et al. 2019 |
| **Tiger** |  |  |  |  |  |  |  |
| *Panthera tigris altaica* | Belgium | 1988 | Captive | 1 | 1 (100) | Mouse bioassay | Dorny and Fransen 1989 |
| *Panthera tigris* | Thailand^‡^ | 1987-1989 | Free ranging | 19 | 0 (0.0) | Microscopy | Patton and Rabinowitz 1994 |
| *Panthera tigris* | Czech Republic | 1995-1996 | Captive^**^ | 188 | 0 (0.0) | Microscopy | Lukesova and Literak 1998 |
| *Panthera tigris* | Mexico^f^ | 2015-2016 | Captive | 8 | 1 (12.5) | Nested PCR | Gomez-Rios et al. 2019 |

**Table 2.** Continued

| **Species** | | | **Location** | | **Period** | | **Status** | | **Sample size** | | **Positive (%)** | | **Method** | **Ref.** | |
| --- | --- | --- | --- | --- | --- | --- | --- | --- | --- | --- | --- | --- | --- | --- | --- |
| **European wildcat** | | |  | |  | |  | |  | |  | |  |  | |
| *Felis silvestris* | | | Czech Republic | | 1995-1996 | | Captive^**^ | | 175 | | 14 (8.0) | | Microscopy | Lukesova and Literak 1998 | |
| **Jungle cat** | | |  | |  | |  | |  | |  | |  |  | |
| *Felis chaus* | | | Czech Republic | | 1995-1996 | | Captive^**^ | | 121 | | 0 (0.0) | | Microscopy | Lukesova and Literak 1998 | |
| **Eurasian lynx** | | |  | |  | |  | |  | |  | |  |  | |
| *Lynx lynx* | | | Czech Republic | | 1995-1996 | | Captive^**^ | | 309 | | 0 (0.0) | | Microscopy | Lukesova and Literak 1998 | |
| *Lynx lynx* | | | Sweden | | 1996-1998 | | Free ranging | | 207 | | 0 (0.0) | | Microscopy | Ryser-Degiorgis et al. 2006 | |
| **Canadian lynx** | | |  | |  | |  | |  | |  | |  |  | |
| *Lynx canadensis* | | | Canada (Quebec) | | 2009-2010 | | Free ranging | | 84 | | 0 (0.0) | | Microscopy | Simon et al. 2013 | |
| **Bobcat** | | |  | |  | |  | |  | |  | |  |  | |
| *Felis rufus* | | | USA | | 1975 | | Free ranging | | 9 | | 3 (33.3) | | Mouse bioassay | Marchiondo et al. 1976 | |
| *Felis rufus* | | | USA (Virginia) | | 1977-1978 | | Free ranging | | 150 | | 0 (0.0) | | Microscopy | Oertley and Walls 1980 | |
| *Felis rufus* | | | Czech Republic | | 1995-1996 | | Captive^**^ | | 301 | | 0 (0.0) | | Microscopy | Lukesova and Literak 1998 | |
| *Felis rufus* | | | USA (California) | | 2006-2009 | | Free ranging | | 16 | | 2 (12.5) | | Microscopy | VanWormer et al. 2013 | |
| *Felis rufus* | | | Mexico^f^ | | 2015-2016 | | Captive | | 1 | | 0 (0.0) | | Nested PCR | Gomez-Rios et al. 2019 | |
| **Caracal** | | |  | |  | |  | |  | |  | |  |  | |
| *Caracal caracal* | | | Czech Republic | | 1995-1996 | | Captive^**^ | | 106 | | 0 (0.0) | | Microscopy | Lukesova and Literak 1998 | |
| *Caracal caracal* | | | Mexico^f^ | | 2015-2016 | | Captive | | 1 | | 0 (0.0) | | Nested PCR | Gomez-Rios et al. 2019 | |
| **Serval** |  |  | |  | |  | |  | |  | |  | | |  |
| *Leptailurus serval* | | | Czech Republic | | 1995-1996 | | Captive^**^ | | 146 | | 0 (0.0) | | Microscopy | Lukesova and Literak 1998 | |
| **Geoffroy's cat** | | |  | |  | |  | |  | |  | |  |  | |
| *Leopardus geoffroyi* | | | Czech Republic | | 1995-1996 | | Captive^**^ | | 39 | | 4 (10.2) | | Microscopy | Lukesova and Literak 1998 | |
| **Snow leopard** | | |  | |  | |  | |  | |  | |  |  | |
| *Panthera uncia* | | | Czech Republic | | 1995-1996 | | Captive^**^ | | 76 | | 0 (0.0) | | Microscopy | Lukesova and Literak 1998 | |
| **Pallas's cat** | | |  | |  | |  | |  | |  | |  |  | |
| *Otocolobus manul* | | | Mongolia | | 2000-2001 | | Free ranging | | 15 | | 0 (0.0) | | PCR | Brown et al. 2005 | |
| **Cheetah** | | |  | |  | |  | |  | |  | |  |  | |
| *Acinonyx jubatus* | | | Czech Republic | | 1995-1996 | | Captive^**^ | | 15 | | 0 (0.0) | | Microscopy | Lukesova and Literak 1998 | |
| **Lion** | | |  | |  | |  | |  | |  | |  |  | |
| *Panthera leo* | | | Czech Republic | | 1995-1996 | | Captive^**^ | | 96 | | 0 (0.0) | | Microscopy | Lukesova and Literak 1998 | |
| *Panthera leo* | | | Zimbabwe | | 2011 | | Captive | | 30 | | 5 (16.7) | | Microscopy | Mukarati et al. 2013 | |
| *Panthera leo* | | | Mexico^f^ | | 2015-2016 | | Captive | | 6 | | 0 (0.0) | | Nested PCR | Gomez-Rios et al. 2019 | |
| **Geoffrey’s cat** (*Leopardus geoffroyi*)  **Pampas cat** (*Leopardus colocolo*)  **Jaguarundi** (*Puma yagouaroundi*) | | | Argentina | | 1977 | | Free ranging | | 73^e^ | | 27 (37.0) | | Pig/mouse bioassy | Pizzi et al. 1978 | |

^a^ Feline scientific names listed according to International Union for Conservation of Nature (IUCN) classifications; ^b^ Iriomote-jima Island; ^c^ The number and species of felids with confirmed shedding of *T. gondii* is unknown as *T. gondii*-like oocysts were pooled for bioassay; ^*^Cockscomb Basin of Belize; ^**^ Czech zoos; ^¶^ 16 fecal samples (1 positive) collected from dead mountain lions, 7 fecal samples (1 positive) collected in the environment from an unknown number of mountain lions. Overlap may exist between the sampled mountain lions and environmentally collected feces; † Vancouver Island, British Columbia; ‡ Huai Kha Wildlife Sanctuary; ^d^ 45 fecal samples collected in the environment from an unknown number of Iriomote cats; ^e^ The number and species of felids with confirmed shedding of *T. gondii* is unknown as *T. gondii*-like oocysts were pooled for bioassay; ^f^ Mexican zoos in Quintana Roo, Yucatan, Chiapas, and Morelos.

**References**

1. Patton, S., Rabinowitz, A., Randolph, S., Johnson, S.S., 1986. A coprological survey of parasites of wild neotropical felidae. Journal of Parasitology 72, 517–520.
2. Lukesova D., Literak I. 1998. Shedding of Toxoplasma gondii oocysts by Felidae in zoos in the Czech Republic. Veterinary Parasitology 74, 1–7.
3. Gomez-Rios A, Ortega-Pacheco A, Gutierrez-Blanco E, Acosta-Viana KY, Guzman-Marin E, Guiris-Andrade MD, Hernandez-Cortazar IB, Lopez-Alonso R, Cruz-Alda E, Jimenez-Coello M. *Toxoplasma gondii* in Captive Wild Felids of Mexico: Its Frequency and Capability to Eliminate Oocysts. Vector Borne and zoonotic diseases. 2019
4. Marchiondo, A.A., Duszynski, D.W., Maupin, G.O., 1976. Prevalence of antibodies to *Toxoplasma gondii* in wild and domestic animals of New Mexico, Arizona and Colorado. Journal of Wildlife Diseases 12, 226–232.
5. Aramini, J.J., Stephen, C., Dubey, J.P., 1998. *Toxoplasma gondii* in Vancouver Island cougars (*Felis concolor vancouverensis*): serology and oocyst shedding. Journal of Parasitology 84, 438–440.
6. Akuzawa, M., Mochizuki, M., Yasuda, N., 1987. Hematological and parasitological study of the Iriomote cat (*Prionailurus iriomotensis*). Canadian Journal of Zoology 65, 946–949
7. Patton S, Rabinowitz AR. Parasites of wild felidae in Thailand: a coprological study. Journal of Wildlife Diseases 1994;30:472–5.
8. Dorny, P., Fransen, A.J., 1989. Toxoplasmosis in a Siberian tiger (*Panthera tigris altaica*). Veterinary Record 125, 647.
9. Brown, AS, Lappin MR, Brown JL, Munkhtsog B, Swanson WF. Exploring the ecologic basis for extreme susceptibility of Pallas cats (*Otocolobus manul*) to fatal toxoplasmosis. Journal of Wildlife Diseases, 2005; 41(4):691-700
10. Pizzi, H.L., Rico, C.M., Pessat, O.A.N., 1978. Hallazgo del ciclo ontogenico selvatico del *Toxoplasma gondii* en felidos salvajes (*Oncifelis geofroyi*, *Felis colocolo*) de la Provincia de Cordoba. Revista Militar de Veterinaria 25, 293–300.
11. Vanwormer E, Conrad PA, Miller MA, Melli AC, Carpenter TE, Mazet JA*. Toxoplasma gondii*, source to sea: higher contribution of domestic felids to terrestrial parasite loading despite lower infection prevalence. Ecohealth. 2013;10(3):277-89
12. Simon A, Poulin MB, Rousseau AN, Dubey JP, Ogden NH. [Spatiotemporal dynamics of *Toxoplasma gondii* infection in Canadian lynx (*Lynx canadensis*) in western Quebec, Canada.](http://ovidsp.tx.ovid.com/sp-3.31.1b/ovidweb.cgi?&S=IIPAFPDDHLDDEIPFNCEKOBGCCILLAA00&Complete+Reference=S.sh.69|120|1)  Journal of Wildlife Diseases 2013;49(1):39-48
13. Oertley KD, Walls KW. [Prevalence of antibodies to *Toxoplasma gondii* among bobcats of West Virginia and Georgia.](http://ovidsp.tx.ovid.com/sp-3.31.1b/ovidweb.cgi?&S=IIPAFPDDHLDDEIPFNCEKOBGCCILLAA00&Complete+Reference=S.sh.69|224|1)  Journal of the American Veterinary Medical Association 1980;177(9):852-853
14. Mukarati NL, Vassilev GD, Tagwireyi WM, Tavengwa M. [Occurrence, prevalence and intensity of internal parasite infections of African lions (*Panthera leo*) in enclosures at a recreation park in Zimbabwe.](http://ovidsp.tx.ovid.com/sp-3.31.1b/ovidweb.cgi?&S=IIPAFPDDHLDDEIPFNCEKOBGCCILLAA00&Complete+Reference=S.sh.69|259|1)  Journal of Zoo and Wildlife Medicine 2013;44(3):686-693.
15. Ryser-Degiorgis, M.P., Jakubek, E.B., af Segerstad, C.H., Brojer, C., Morner, T., Jansson, D.S., Lunden, A., Uggla, A., 2006. Serological survey of *Toxoplasma gondii* infection in free-ranging Eurasian lynx (*Lynx lynx*) from Sweden. Journal of Wildlife Diseases 42, 182–187.
