# Supplementary material for: Toxoplasma gondii infection in domestic and wild felids as public health concerns: a systematic review and meta-analysis
Source: Sci Rep. 2021 May 4;11:9509. doi: 10.1038/s41598-021-89031-8 (PMC8097069; doi:10.1038/s41598-021-89031-8)
Supplement: Supplementary file 8 — Supplementary Information 8. [file 41598_2021_89031_MOESM8_ESM.doc]

**Table S6.** Summary of studies focused on detection of soil contamination with *Toxoplasma*-like oocysts (or *T. gondii*-DNA) (sorted by year of publication)

| **Location** | **Period** | **Source** | **Sample size** | **Positive (%)** | **Method** | **Ref.** |
| --- | --- | --- | --- | --- | --- | --- |
| Panama (Panama City) | N.S. | N.S. | 924 | 10 (1.1) | Mouse bioassay | Frenkel et al. 1995 |
| Poland (Northern) | 2006-2007 | SP, PG, PP, RD | 101 | 18 (17.8) | Conventional PCR | Lass et al. 2009 |
| Brazil (São Paulo) | 2008 | PS, PG | 31 | 10 (32.3) | Mouse bioassay | dos Santos et al. 2010 |
| China (Hubei) | 2009-2010 | PF | 95 | 36 (37.9) | LAMP | Du et al. 2012 |
| China (Wuhan) | 2009-2010 | PP | 252 | 58 (23.0) | LAMP | Du et al. 2012 |
| Iran (Tehran) | 2008-2009 | PP | 150 | 13 (8.7) | Conventional PCR | Tavalla et al. 2013 |
| Pakistan (Multan) | 2011 | PG, PP, SP | 250 | 12 (4.8) | Conventional PCR | Ajmal et al. 2013 |
| China (Northwestern) | 2013 | PP | 268 | 34 (12.7) | Semi-nested PCR | Wang et al. 2014 |
| France (Northern) | 2011 | CF, G, F | 243 | 71 (29.2) | Real time-PCR | Gotteland et al. 2014 |
| Iran (Arak) | 2013 | PP | 60 | 3 (5.0) | Conventional PCR | Solymane et al. 2014 |
| China (Harbin) | 2014-2015 | PP, PS | 9,420 | 2,853 (30.3) | Real time-PCR | Gao et al. 2016 |
| China (Nanjing) | 2013-2014 | CF | 700 | 7 (1.0) | Conventional PCR | Liu et al. 2017 |
| France (Ardennes) | 2014 | DF | 558 | 278 (49.8) | Real time-PCR | Simon et al. 2017 |
| Iran (Ahvaz) | 2011-2012 | PP | 200 | 18 (9.0) | Conventional PCR | Saki et al. 2017 |

PCR, Polymerase chain reaction; LAMP, Loop-mediated isothermal amplification; IOM, Inoculated orally into mice; SP, Sand pits; PP, Public parks; PG, Playgrounds; RD, Rubbish dumps; CF, Crop field; G, Grassland; F, Forest; PS, Public school; CF, Chicken farm; PF, Pig farm; DF, Dairy farm

**References**

1. Solymane H, Eslamirad Z, Bayat M, Hajihossein R. Molecular Detection of *Toxoplasma gondii* Oocytes in the Soil from the Public Parks of the Arak City, Iran. Res Mol Med. 2014; 2 (1): 35-38
2. Tavalla M, Oormazdi H, Akhlaghi L, Shojaee S, Razmjou E, Hadighi R, Meamar AR. Genotyping of *Toxoplasma gondii* Isolates from Soil Samples in Tehran, Iran. Iranian J Parasitol. 2013; 8(2):227-233
3. Saki J, Khademvatan S, Yousefi E, Tavalla M, Abdizadeh R. Detection and genotyping of *Toxoplasma gondii* isolated from soil in Ahvaz, southwest of Iran. J Parasit Dis. 2017;41(1):202-205
4. dos Santosa TR, Nunes CM, Luvizotto MCR, de Moura AB, Lopes WDZ, da Costa AJ, Bresciani KDS. Detection of *Toxoplasma gondii* oocysts in environmental samples from public schools. Veterinary Parasitology 2010;171:53-57
5. Du F, Zhang Q, Yu Q, Hu M, Zhou Y, Zhao J. Soil contamination of *Toxoplasma gondii* oocysts in pig farms in central China. Veterinary Parasitology 2012;187:53-56
6. Du F, Feng HL,. Nie H, Tu P, Zhang QL, Hu M, Zhou YQ, Zhao JL. Survey on the contamination of *Toxoplasma gondii* oocysts in the soil of public parks of Wuhan, China. Veterinary Parasitology 2012;184:141-146
7. Wang M, Meng P, Ye Q, Pu YH, Yang XY, Luo JX, Zhang NZ, Zhang DL. Detection of *Toxoplasma gondii* oocysts in soils in Northwestern China using a new semi-nested PCR assay. BMC veterinary research 2014;10(1):238.
8. Gao X, Wang H, Wang H, Qin H, Xiao J. Land use and soil contamination with *Toxoplasma gondii* oocysts in urban areas. Science of the Total Environment 2016;15(568):1086-91.
9. Liu XC, He Y, Han DG, Zhang ZC, Li K, Wang S, Xu LX, Yan RF, Li XR. Detection of *Toxoplasma gondii* in chicken and soil of chicken farms in Nanjing region, China. Infectious Diseases of Poverty 2017; 6:62
10. Gotteland C, Gilot-Fromont E, Aubert D, Poulle ML, Dupuis E, Dardé ML, Forin-Wiart MA, Rabilloud M, Riche B, Villen I. Spatial distribution of *Toxoplasma gondii* oocysts in soil in arural area: Influence of cats and land use. Veterinary Parasitology 2014;205:629-637
11. Simon JA, Kurdzielewicz S, Jeanniot E, Dupuis E, Marnef F, Aubert D, Villena I, Poulle ML. Spatial distribution of soil contaminated with *Toxoplasma gondii* oocysts in relation to the distribution and use of domestic cat defecation sites on dairy farms. International Journal for Parasitology, 2017; ;47(6):357-67.
12. Frenkel, J.K., Hassanein, K.M., Hassanein, R.S., Brown, E., Thulliez, P., Quintero- Nunez, R., 1995. Transmission of *Toxoplasma gondii* in Panama City, Panama: a five-year prospective cohort study of children, cats, rodents, birds, and soil. American Journal of Tropical Medicine and Hygiene 53, 458–468.
13. Lass A, Pietkiewicz H, Modzelewska E, Dumètre A, Szostakowska B, Myjak P. Detection of *Toxoplasma gondii* oocysts in environmental soil samples using molecular methods. Eur J Clin Microbiol Infect Dis. 2009;28(6):599-605
14. Ajmal A, Maqbool A, Qamar, MF, Ashraf K, Anjum AA. [Detection of *Toxoplasma gondii* in environmental matrices (water, soil, fruits and vegetables).](http://ovidsp.tx.ovid.com/sp-3.31.1b/ovidweb.cgi?&S=IIPAFPDDHLDDEIPFNCEKOBGCCILLAA00&Complete+Reference=S.sh.69|167|1)  African Journal of Microbiology Research 2013;7(16):1505-1511
